# Negative cooperativity upon hydrogen bond-stabilized O_2_ adsorption in a redox-active metal–organic framework

**DOI:** 10.1038/s41467-020-16897-z

**Published:** 2020-06-18

**Authors:** Julia Oktawiec, Henry Z. H. Jiang, Jenny G. Vitillo, Douglas A. Reed, Lucy E. Darago, Benjamin A. Trump, Varinia Bernales, Harriet Li, Kristen A. Colwell, Hiroyasu Furukawa, Craig M. Brown, Laura Gagliardi, Jeffrey R. Long

**Affiliations:** 1 Department of Chemistry, University of California, Berkeley, CA 94720 USA; 2 Department of Chemistry and Supercomputing Institute, University of Minnesota, Minneapolis, MN 55455 USA; 3 National Institute of Standards and Technology, Center for Neutron Research, Gaithersburg, MD 20899 USA; 4 Department of Aeronautics and Astronautics, Massachusetts Institute of Technology, Cambridge, MA 02139 USA; 5 Department of Chemical and Biomolecular Engineering, University of California, Berkeley, CA 94720 USA; 6 Materials Sciences Division, Lawrence Berkeley National Laboratory, Berkeley, CA 94720 USA; 7 Chemical and Biomolecular Engineering, University of Delaware, Newark, DE 19716 USA

**Keywords:** Inorganic chemistry, Materials chemistry, Metal-organic frameworks

## Abstract

The design of stable adsorbents capable of selectively capturing dioxygen with a high reversible capacity is a crucial goal in functional materials development. Drawing inspiration from biological O_2_ carriers, we demonstrate that coupling metal-based electron transfer with secondary coordination sphere effects in the metal–organic framework Co_2_(OH)_2_(bbta) (H_2_bbta = 1*H*,5*H*-benzo(1,2-*d:*4,5-*d*′)bistriazole) leads to strong and reversible adsorption of O_2_. In particular, moderate-strength hydrogen bonding stabilizes a cobalt(III)-superoxo species formed upon O_2_ adsorption. Notably, O_2_-binding in this material weakens as a function of loading, as a result of negative cooperativity arising from electronic effects within the extended framework lattice. This unprecedented behavior extends the tunable properties that can be used to design metal–organic frameworks for adsorption-based applications.

## Abstract (web summary)

Oxygen capture is attractive for catalysis, sensing, and separations, but engineering stable and selective adsorbents is challenging. Here the authors combine metal-based electron transfer with secondary coordination sphere effects in a metal-organic framework, leading to strong and reversible O_2_ adsorption that also exhibits negative cooperativity.

## Introduction

Allosteric binding of substrates to biomolecules is effortlessly accomplished in nature, but engineering synthetic materials to display similarly responsive behavior remains an ongoing challenge. In addition to non-cooperative substrate binding (e.g., O_2_ in myoglobin), biomolecules can exhibit cooperativity arising from structural changes that strengthen or weaken interactions with substrates. Positive cooperativity can lead to enhanced response to ligands under certain conditions (e.g., O_2_ binding in hemoglobin), while negative cooperativity generates a weakened response to successive binding events and enables biomolecules to maintain molecule sensitivity over greater concentration ranges than observed for non-cooperative or positively cooperative proteins (e.g., insulin binding to the insulin receptor)^1–4^. The ability to reproduce similar performance in synthetic materials could be advantageous for a multitude of potential applications, including catalysis^5,6^, sensing^7^, and gas separations^8,9^.

**Fig. 1:**
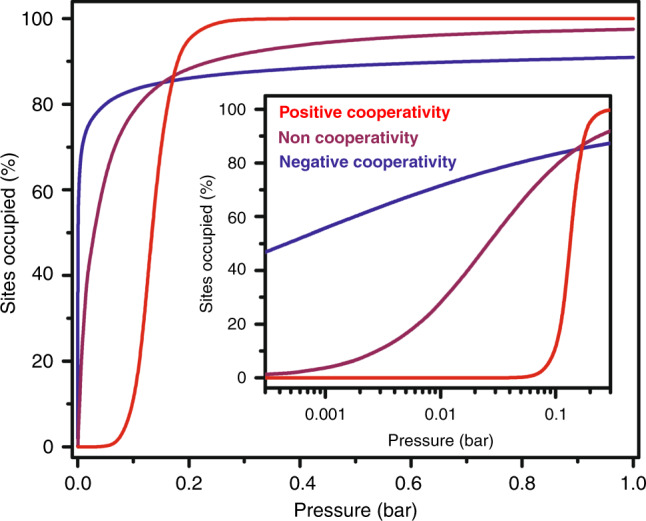
Illustration of adsorption isotherm variability with cooperativity. Isotherms of positively cooperative (red line), noncooperative (purple line), and negatively cooperative (blue line) systems exhibit significant differences in shape, due to the nature of the adsorbate-adsorbent interactions. In particular, negatively cooperative systems can reach half occupancy at significantly lower pressures than noncooperative and positively cooperative systems but generally require much higher pressures to reach saturation. The inset graph displays the low pressure regime (<0.2 bar) on a logarithmic scale to highlight differences in isotherm slope.

Metal–organic frameworks provide an intriguing platform for the design and study of cooperative adsorption, owing to their crystallinity, chemical versatility, and extended network structure that can readily propagate minor structural changes^10,11^. Indeed, some of us have recently shown that cooperative CO_2_ uptake in alkyldiamine-appended M_2_(dobpdc) (M = Mg, Mn, Fe, Co, Ni, Zn; H_4_(dobpdc) = 4,4′-dioxidobiphenyl-3,3′-dicarboxylic acid) occurs as a result of the extended formation of ammonium carbamate chains down the framework pores^8^, while the propagation of an iron(II) spin transition upon CO binding gives rise to its cooperative adsorption in the frameworks Fe_2_Cl_2_(bbta) (H_2_bbta = 1*H*,5*H*-benzo(1,2-*d*:4,5-*d*′)bistriazole) and Fe_2_Cl_2_(btdd) (H_2_btdd = bis(1*H*-1,2,3-triazolo[4,5-*b*],[4′,5′-*i*])dibenzo[1,4]dioxin)^9^. The adsorption behavior of these materials stands in stark contrast to the non-cooperative, Langmuir-type uptake that is typical of frameworks featuring isolated binding sites (Fig. 1). As a result, these cooperative adsorbents exhibit exceptionally high selectivities and capacities and can be regenerated with far lower energy costs than traditional adsorbents, rendering them of interest for industrial separation applications.

**Fig. 2:**
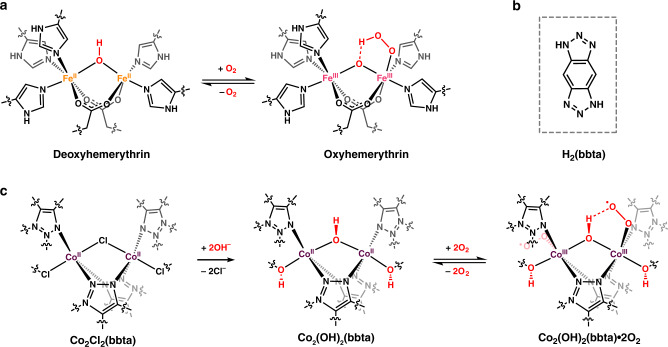
Hydrogen bond stabilization of dioxygen binding in hemerythrin. **a** Illustration of O_2_ binding in the metalloprotein hemerythrin, which shows the critical role that the bridging hydroxo group of the active site plays in the stabilization of the resulting iron(III)-hydroperoxo species. **b** The structure of the H_2_bbta ligand, 1*H*,5*H*-benzo(1,2-*d:*4,5-*d*′)bistriazole), used in this work. **c** Illustration of post-synthetic modification of Co_2_Cl_2_(bbta) by anion exchange with OH^−^ (left) to form Co_2_(OH)_2_(bbta) (middle)^33^. The bridging hydroxo groups in this structure provide stabilization to bound O_2_ (right) in a manner analogous to oxyhemerythrin.

The development of a metal–organic framework capable of cooperative O_2_ adsorption could be advantageous for a number of applications in industry, including energy-efficient O_2_ production^11,12^, O_2_ storage^13–15^, and catalysis^16,17^. Iron-^18,19^, chromium-^20,21^, manganese-^22^, copper-^23^, and cobalt-based^24,25^ frameworks have previously been shown to bind O_2_ via reversible reduction upon metal coordination, but the poor chemical stability of many of these materials to O_2_ limits their cyclable capacity under ambient conditions. In the search for improved, cooperative adsorbents for O_2_ capture, the active sites of proteins such as hemoglobin, hemerythrin, and hemocyanin can serve as sources of inspiration. These sites feature reducing transition metal centers that bind O_2_ via electron transfer to reversibly generate metal-superoxo or metal-peroxo species^26,27^, and crucially these species are often stabilized through directed hydrogen-bonding interactions^28^. For example, histidine residues in hemoglobin hydrogen bond to the iron-superoxo formed upon O_2_ uptake^28,29^, while in hemerythrin a bridging hydroxo group transfers a proton to iron-bound dioxygen to form a hydroperoxo species (Fig. 2a)^30^. These hydrogen bonding interactions are thought to play a key role in reversible O_2_ binding in these proteins, and may also be advantageous for the development of metal–organic frameworks for selective and reversible O_2_ capture. Here, we report the detailed characterization of O_2_ adsorption in the frameworks Co_2_X_2_(bbta) (X^−^ = Cl^−^, OH^−^)^31–34^ and show that Co_2_(OH)_2_(bbta) is able to capture O_2_ with high affinity by leveraging both electron transfer and hydrogen bonding interactions (Fig. 2c). Remarkably, as a result of the close metal contacts within Co_2_(OH)_2_(bbta), O_2_ interacts more weakly with the cobalt(II) centers upon increased loading, providing the first clear example of an adsorbent material that exhibits negative cooperativity upon gas binding.

## Results

### Design, synthesis, and gas adsorption properties

**Fig. 3:**
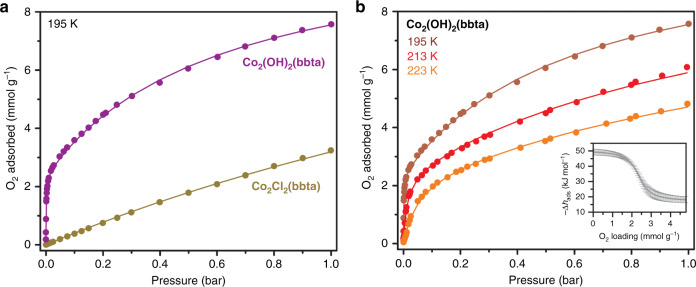
Oxygen adsorption data. **a** Oxygen adsorption isotherms obtained at 195 K for Co_2_Cl_2_(bbta) (gold circles) and Co_2_(OH)_2_(bbta) (purple circles). **b** Oxygen adsorption isotherms obtained at 195, 213, and 223 K for Co_2_(OH)_2_(bbta) (maroon, red, and orange symbols respectively). Symbols represent data points and lines represent fits using a dual-site Langmuir model. The inset depicts the differential enthalpy of adsorption for O_2_ adsorption in Co_2_(OH)_2_(bbta) (gray line, with error bars), determined using the Clausius–Clapeyron equation as described in the Methods. Source data are provided as a source data file.

In our search for a cooperative, O_2_-selective metal–organic framework, we initially investigated the known framework Co_2_Cl_2_(bbta), which features a high density of coordinatively-unsaturated cobalt(II) ions^31,32^, as well as basic nitrogen-donor ligands that should enhance the reducing potential of the metal centers^24^. However, we found that Co_2_Cl_2_(bbta) exhibits only a weak interaction with oxygen (Fig. 3a), indicating that the ligand environment is not basic enough to facilitate metal-centered O_2_ reduction, and we thus turned our attention to the recently reported derivative Co_2_(OH)_2_(bbta) (Fig. 2c)^33^. Synthesis of this material following a significantly modified procedure (see the Supporting Methods) led to a crystalline compound with a Brunauer–Emmett–Teller surface area of 1470 m^2^ g^−1^ (Supplementary Figs. 2–4).

At 195 K, the O_2_ adsorption isotherm for Co_2_(OH)_2_(bbta) exhibits a sharp rise at low pressures (Fig. 3a), and the material achieves a capacity of ∼3 mmol g^−1^ (0.47 mmol O_2_ per mmol Co) at just 25 mbar. At 1 bar, the capacity is 7.57 mmol g^−1^ (19.5 wt % or 1.17 mmol O_2_ per mmol Co), significantly higher than that of Co_2_Cl_2_(bbta) under the same conditions (3.24 mmol g^−1^, or 0.63 mmol O_2_ per mmol Co) and higher than any other cobalt(II)-based framework studied for O_2_ adsorption^24,25^. To quantify the strength of the metal–O_2_ interactions in Co_2_(OH)_2_(bbta), we determined the differential enthalpy of O_2_ adsorption (Δ*h*_ads_) from the Clausius–Clapeyron equation, using parameters extracted from a dual-site Langmuir fit of adsorption isotherms collected at 195, 213, and 223 K (Fig. 3b and Methods). At low loadings, O_2_ occupies a primary binding site with Δ*h*_ads_ = −49 ± 2 kJ mol^−1^, which is more than three times the differential enthalpy of O_2_ adsorption in Co_2_Cl_2_(bbta), and with a saturation capacity of 2.46 ± 0.02 mmol g^−1^ (approximately 0.38 mmol O_2_ per mmol Co; Fig. 3b). Consistent with these results, density functional theory (DFT) calculations performed on various model systems indicate that Co_2_(OH)_2_(bbta) has a much higher affinity for O_2_ than Co_2_Cl_2_(bbta) (Supplementary Tables 43 and 47). In tandem with the comparatively high adsorption site density in Co_2_(OH)_2_(bbta) per formula weight^24,25^, this large enthalpy of adsorption explains the high uptake capacity of the material. The primary binding site also exhibits a large differential entropy of adsorption (Δ*s*_ads_) of 186 ± 7 J mol^−1^ K^−1^, leading to a significant adsorption temperature dependence that accounts for the low uptake at 298 K (Fig. 3b; Supplementary Figs. 6 and 15). With increasing loading, O_2_ occupies a secondary binding site with Δ*h*_ads_ = −18 ± 2 kJ mol^−1^. Despite the high differential enthalpy observed at low loadings, O_2_ adsorption was found to be fully reversible under all temperatures and pressures studied. Importantly, the capacity and differential enthalpy of O_2_ adsorption in Co_2_(OH)_2_(bbta) are substantially greater than those values measured and determined for N_2_ (Supplementary Figs. 10, 11, and 14). Ideal adsorbed solution theory^35^ was used to calculate the O_2_/N_2_ selectivities for a mixture containing 21% O_2_ and 79% N_2_, representative of air (Supplementary Table 5). At 195 K, the calculated selectivity is 42, similar to that reported for the framework Co-BTTri (Co_3_[(Co_4_Cl)_3_(BTTri)_8_]_2_; H_3_BTTri = 1,3,5-tri(1*H*-1,2,3-triazol-5-yl)benzene) under the same conditions (41)^24^, and this value decreases with increasing temperature to 14 at 223 K. These selectivities correspond, respectively, to 91% and 79% pure adsorbed O_2_ on Co_2_(OH)_2_(bbta) under equilibrium conditions. Notably, Co_2_(OH)_2_(bbta) also exhibits an O_2_ working capacity of 1.5 mmol g^−1^ (4.6 wt %) upon lowering the pressure from 0.21 to 0.05 bar at 195 K, which is higher than the values reported for Co-BTTri and Fe_2_(dobdc)^24^. Together, these results suggest that Co_2_(OH)_2_(bbta) could potentially be useful for air separation in a vacuum-swing adsorption process.

### Structural, spectroscopic, and magnetic characterization

**Fig. 4:**
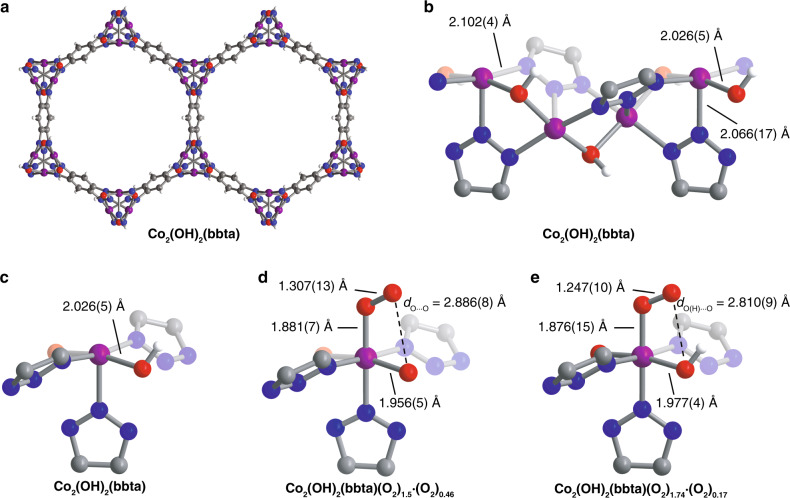
Structures of activated and O_2_-dosed Co_2_(OH)_2_(bbta). **a**–**c** Structures of activated Co_2_(OH)_2_(bbta) as determined by Rietveld refinement against neutron powder diffraction data obtained at 8 K, showing **a** the hexagonal framework channels with the view down the c axis, **b** a truncated view of the coordinatively-unsaturated metal centers lining the pores, and **c** a single coordinatively-unsaturated cobalt(II) center. **d** Structure of Co_2_(OH)_2_(bbta) dosed with 1 bar of O_2_, as determined from Rietveld refinement against powder X-ray diffraction data obtained at 195 K. Hydrogen atom positions could not be determined from the measurement. The 0.46 equivalents of O_2_ found within the pores are not shown for clarity. **e** Structure of Co_2_(OH)_2_(bbta)(O_2_)_1.74_·(O_2_)_0.17_ determined from Rietveld refinement against neutron powder diffraction data of Co_2_(OH)_2_(bbta) dosed with 1 equivalent of O_2_ per cobalt at 8 K. The 0.17 equivalents of O_2_ found within the pores are not shown for clarity. In **b**, **c**, and **e**, only one orientation is shown for the half-occupancy hydrogen atom of the hydroxo group. Slight deviations in the bond distances derived from the neutron and X-ray diffraction data are likely a result of differences in experimental conditions, as well as the improved scattering of the O atoms in neutron diffraction. Purple, red, blue, gray, and white spheres represent Co, O, N, C, and H atoms, respectively.

The solid-state structure of Co_2_(OH)_2_(bbta) was determined from Rietveld refinement against neutron powder diffraction data collected on the desolvated material (obtained under vacuum at 6 K; see Fig. 4a). The framework features hexagonal channels lined with rows of coordinatively-unsaturated cobalt(II) centers that are bridged by a combination of hydroxo and triazolate groups (Fig. 4b,c). The Co–N bond lengths (2.066(17) and 2.102(4) Å) are within the range expected for nitrogen-donor ligands bound to high-spin cobalt(II)^36^. Analysis of in situ X-ray powder diffraction data for a sample of Co_2_(OH)_2_(bbta) dosed with increasing pressures of O_2_ at 195 K revealed a gradual shift in the allowed reflections to higher angles, indicative of a unit cell contraction with increased O_2_ loading (Supplementary Fig. 23). For example, dosing with 1 bar of O_2_ resulted in an 8.6% decrease in the unit cell volume (Supplementary Tables 9 and 10) relative to the activated framework, concomitant with a decrease in the cobalt–ligand bond distances, from 2.072(6) to 1.956(5) Å (*d*_Co–OH_) and from 2.115(8)/2.007(13) Å to 1.932(10)/1.956(14) Å (*d*_Co–N_). Rietveld refinements against this variable-pressure data yielded structural models consistent with the presence of end-on cobalt(III)-superoxo species under all conditions (Fig. 4d; ∠Co–O–O = 119.9(4)°, 75(1)% occupancy)^26^. This metrical data is supported by values determined from periodic DFT calculations (*d*_O–O_ = 1.30 Å and and *d*_Co–O_ = 1.87 Å for Co_2_(OH)_2_(bbta)·2O_2_; see Supplementary Information Section 16). While there is evidence for physisorption of O_2_ along the pore walls at high pressures, the superoxo site occupancy also grows with increasing pressure at 195 K, a change associated with an increasing unit cell volume contraction (Supplementary Fig. 25).

**Fig. 5:**
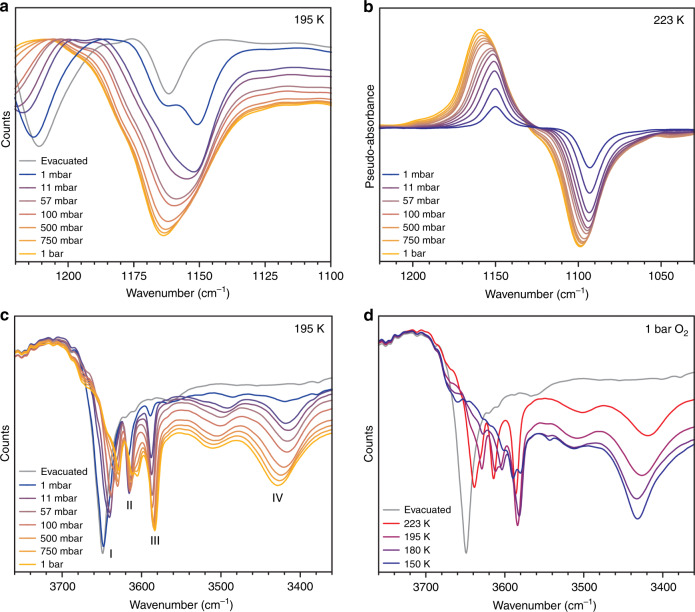
Spectroscopic data for evacuated and O_2_-dosed Co_2_(OH)_2_(bbta). **a** Raw DRIFTS data obtained at 195 K by dosing Co_2_(OH)_2_(bbta) with variable pressures of ^16^O_2_. **b** DRIFTS spectra obtained at 223 K for Co_2_(OH)_2_(bbta) dosed with increasing pressures of ^16^O_2_, subtracted from the spectra of Co_2_(OH)_2_(bbta) dosed with corresponding pressures of ^18^O_2_. The ν(O–O) band of the adsorbed superoxo species appears as a positive peak for ^16^O_2_ and a negative peak for ^18^O_2_. **c** Raw DRIFTS spectra obtained at 195 K for desolvated and ^16^O_2_-dosed Co_2_(OH)_2_(bbta). The broad ν(OH) band from 3416–3427 cm^−1^ (IV) is assigned to a cobalt(III)-hydroxo hydrogen bonded to the proximal oxygen atom of an adsorbed superoxo species. Sharper peaks (I–III) correspond to additional framework hydroxo groups, as indicated in Supplementary Fig. 54. **d** Raw DRIFTS spectra obtained at 223 K for desolvated Co_2_(OH)_2_(bbta) and for Co_2_(OH)_2_(bbta) dosed with 1 bar of ^16^O_2_ obtained at 223, 195, 180, and 150 K.

In situ diffuse reflectance infrared Fourier transform spectroscopy (DRIFTS) was used to further investigate the nature of the Co–O_2_ interaction upon O_2_ adsorption in Co_2_(OH)_2_(bbta). Dosing the framework with 1 mbar of ^16^O_2_ at 195 K afforded a new peak in the spectrum at 1151 cm^−1^ (Fig. 5a), which can be assigned to the *ν*(O–O) band of a superoxide (O_2_^−^) species bound end-on to cobalt(III) (1200–1100 cm^−1^)^26^. This peak shifts by 57 cm^−1^ to 1094 cm^−1^ when the material is dosed with 1 mbar of ^18^O_2_ (Fig. 5b), close to the isomer shift of 69.1 cm^−1^ predicted from the single harmonic oscillator model for O_2_ (Supplementary Figs. 49 and 50)^37^. Additionally, the superoxo overtone shifts from 2288 to 2182 cm^−1^ (Supplementary Fig. 46). Consistent with our experimental results, Kohn-Sham DFT calculations predicted the formation of superoxo species exhibiting an O–O stretching frequency shift of ∼400 cm^–1^ with respect to the gas phase value (Supplementary Information Section 17). As observed in the isotherm data (Supplementary Fig. 5), O_2_ adsorption was found to be fully reversible upon evacuation of the sample at 195 K or heating at ambient pressure above 250 K.

Variable-temperature d.c. magnetic susceptibility data were collected on samples of Co_2_(OH)_2_(bbta) before and after exposure to O_2_ (*H*_d.c._ = 0.1 T). At 300 K, the molar magnetic susceptibility times temperature (*χ*_M_*T*) for the desolvated material is 4.84 emu K mol^−1^. This value is larger than that predicted for two high-spin *S* = 3/2 cobalt(II) centers (3.75 emu K mol^−1^), likely due to contributions from orbital angular momentum (Supplementary Fig. 63). With decreasing temperature, *χ*_M_*T* decreases gradually, reaching 0.274 emu K mol^−1^ at 2 K. Interestingly, the material also exhibits a negative Curie-Weiss constant (*θ* = −81.0 K; Supplementary Fig. 65) and multireference methods and Kohn-Sham DFT calculations on di- and tetracobalt cluster models of Co_2_(OH)_2_(bbta), predict that an antiferromagnetic ground state for the high-spin cobalt ions is slightly more stable (4 kJ mol^−1^, Supplementary Table 41) than a ferromagnetic ground state (Supplementary Information Section 15). The *χ*_M_*T* curves for Co_2_(OH)_2_(bbta)·O_2_ and Co_2_(OH)_2_(bbta)·2O_2_ exhibit a clear sigmoidal shape (Supplementary Fig. 64), which is reminiscent of that observed for spin crossover materials^38^ and indicates temperature-dependent framework oxidation. At 100 K, the *χ*_M_*T* value for Co_2_(OH)_2_(bbta)·2O_2_ (1 O_2_ per Co) is 2.28 emu K mol^−1^, very close to the spin-only value of 2.25 emu K mol^−1^ expected for one cobalt(III) ion (*S* = 0), one cobalt(II) ion (*S* = 3/2), and one superoxide (*S* = 1/2), in the absence of magnetic exchange. DFT calculations at the PBE0-D3 level suggest that the spin density of cobalt changes from 2.67 to 0.07 upon O_2_ adsorption, while the spin density of O_2_ changes from 1.8 to 0.79 (Supplementary Table 43). Together with the diffraction and DRIFTS data, these results indicate that adsorbed O_2_ is reduced by cobalt(II), yielding a superoxo-bound cobalt(III) species.

### Hydrogen bond stabilization of the superoxo species

In the X-ray powder diffraction derived structure of Co_2_(OH)_2_(bbta) dosed with 1 bar of O_2_ at 195 K, the terminal superoxo oxygen and the hydroxo oxygen are separated by only 2.886(8) Å, within the range for a moderate-strength O(H) · · · O hydrogen bonding interaction (Fig. 4c)^39^. Refinement against neutron powder diffraction data collected on a sample dosed with 1 equiv. of O_2_ per Co and cooled to 8 K notably revealed a hydrogen atom interacting with the terminal superoxo oxygen (∠O–H · · · O = 108°, Fig. 4d)^39,40^. Exclusion of this hydrogen atom from the refinement resulted in significantly worse goodness-of-fit parameters and a clear negative peak in the Fourier difference map (Supplementary Figs. 38 and 39). Notably, such a secondary coordination sphere effect upon O_2_-binding has not previously been observed in a metal–organic framework^41–44^.

The presence of hydrogen bonding is supported by a comparison of the infrared spectra for desolvated and O_2_-dosed Co_2_(OH)_2_(bbta) (Fig. 5c,d). A single *ν*(OH) band is present at 3648 cm^−1^ for the desolvated material (I, Fig. 4c, gray curve), while dosing with 1 mbar of O_2_ leads to additional sharp bands at 3614 and 3586 cm^−1^ (II and III) and a broad band at ∼3420 cm^−1^ (IV). These three peaks redshift with increasing O_2_ pressure up to 1 bar, and band III dominates at high pressures. The sharp features are assigned to –OH moieties that are not participating in hydrogen bonding but are affected by the redox changes of adjacent cobalt centers upon O_2_ binding (Supplementary Fig. 54). Peak IV redshifts by >200 cm^−1^ relative to other *ν*(OH) resonances observed upon oxidation, and its energy is consistent with those reported for *ν*(OH) bands associated with O(H) · · · O interactions between 2.8 and 2.9 Å^40^. Thus, we assign this feature to an –OH adduct hydrogen bonding with a metal-bound superoxo. At higher O_2_ occupancies (Fig. 4c) or lower temperatures (Fig. 4d), the broad band dominates and the sharp features at ∼3610 and ∼3580 cm^−1^ diminish. This trend is expected as O_2_ loading increases and free –OH groups increasingly become engaged in hydrogen bonding. Interestingly, we do not observe full O_2_ occupancy under any of the conditions measured here.

Computed infrared spectra of the hydrogen-bonded cobalt(III)-superoxo in Co_2_(OH)_2_(bbta) successfully simulate the multiplicity of hydroxo peaks observed at low pressures (Supplementary Table 47 and Supplementary Fig. 87). Calculations examining three possible –OH group geometries indicate that the formation of a hydrogen bond to the cobalt(III)-superoxo lowers the O_2_ adsorption energy by more than 20 kJ mol^−1^ (Supplementary Table 43). Significantly, this value is similar in magnitude to the calculated decrease in O_2_ binding energy upon changing the framework from Co_2_Cl_2_(bbta) to Co_2_(OH)_2_(bbta) (∼30 kJ mol^−1^). Stabilization afforded by hydrogen bonding thus appears to be a critical factor leading to the high differential enthalpy of O_2_ adsorption in Co_2_(OH)_2_(bbta). This interaction likely also facilitates stable, reversible O_2_ binding, despite the strong cobalt(II)–O_2_ interaction.

### Negative cooperativity of O_2_ binding

Adsorbate binding at coordinatively unsaturated metal sites is typically much stronger than physisorptive interactions^45,46^. However, in the case of Co_2_(OH)_2_(bbta), the O_2_ occupancy determined from X-ray powder diffraction data at 195 K (75(1)%) is much greater than the saturation capacity of the strong site determined through the dual-site Langmuir model (2.46 ± 0.02 mmol g^−1^, 38% occupancy). Additionally, our analysis of the variable-pressure DRIFTS spectra (Fig. 5a,b) revealed an increase in integrated area that is greater than predicted for the strong binding site, based on analysis of adsorption data (Fig. 57)^47^. These results indicate that the Langmuir model does not accurately describe the O_2_ binding to cobalt at all pressures. Significant IR peak asymmetry at higher loadings could be modeled as two Lorentzian peaks from 1149–1152 cm^−1^ and 1164–1167 cm^−1^, with the higher energy band dominating at higher loadings (Supplementary Figs. 58–62). The same asymmetry is also clearly visible in spectra collected using ^18^O_2_, confirming that it originates from changes in the bound superoxo species (Fig. 5b). The shift of the band to higher energies further suggests a greater amount of electron transfer from the cobalt to adsorbed O_2_ at lower pressures, resulting in a weaker O–O bond. Based on these data, we hypothesize that isolated cobalt(III)-superoxo species dominate at lower pressures, while at higher pressures an increase in the number of adjacent cobalt(III)-superoxo neighbors reduces the driving force for subsequent O_2_ binding and reduction.

**Fig. 6:**
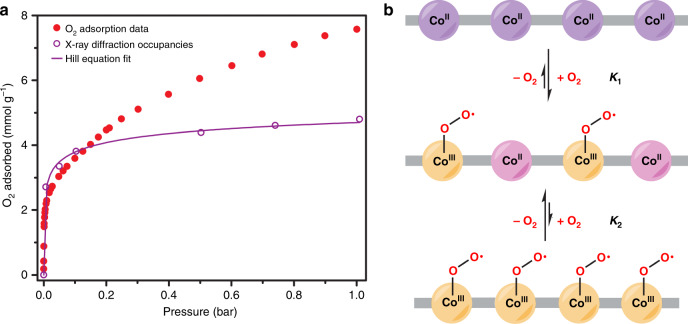
Negative cooperativity of O_2_ binding. **a** Oxygen adsorption isotherm obtained at 195 K for Co_2_(OH)_2_(bbta) (filled red circles) plotted alongside values obtained for superoxo occupancies under similar conditions with powder X-ray diffraction data, multiplied by the theoretical capacity of 6.45 mmol g^−1^ (open purple circles). Modeling these occupancy values using the Hill equation resulted in excellent agreement (solid purple line; Supplementary Information Sections 8). At low pressures, the projected capacity values from the diffraction occupancies are very slightly higher than the experimental adsorption capacities, which may be due to adsorption at defects (e.g., surface sites) that are not reflected in the occupancies. At higher pressures, the significant deviation of the gas adsorption from the projected occupancy capacities is due to physisorption within the pore. Source data are provided as a source data file. **b** Simplified illustration of a chain of cobalt centers in Co_2_(OH)_2_(bbta) running along the c-axis in the activated material (top), following oxidation to generate cobalt(III)-superoxo species (yellow spheres) and unreacted, less electron-rich cobalt(II) ions (pink spheres) (middle), and the fully oxidized cobalt(III)-superoxo chain (bottom). From analysis of the variable-temperature O_2_ adsorption data, the equilibrium constant for partial O_2_ loading (*K*_1_) is estimated to be much larger than the equilibrium constant to achieve 100% occupancy (*K*_2_). As a result, the partially oxidized material dominates under all conditions studied here.

Together with the adsorption and in situ diffraction data, these findings indicate that the interaction of O_2_ with Co_2_(OH)_2_(bbta) changes drastically as a function of O_2_ pressure at 195 K, resulting in incomplete occupation of the open metal sites, even at 1 bar. This attenuated affinity at higher pressures can be attributed to structural and electronic changes that disfavor further superoxo formation following each O_2_ binding event, and is remarkably reminiscent of negative cooperativity in biological systems^2,3,48^. In particular, the initial binding of an O_2_ molecule to a cobalt(II) center creates a comparatively electron-poor cobalt(III) center. Ligand-sharing cobalt(II) centers consequently become less electron donating, and higher pressures of O_2_ are required to facilitate O_2_ adsorption via electron transfer (Fig. 6). Computations further suggest that the reduced availability of hydroxo groups for hydrogen bonding may decrease the O_2_ binding energy at high coverage (Supplementary Information Sections 16 and 17).

X-ray powder diffraction occupancy results were modeled using the Hill equation, yielding a Hill coefficient of *n* = 0.262, consistent with a negatively cooperative adsorption mechanism (Fig. 6 and Supplementary Information Sections 8; generally *n* « 1 for negatively cooperative systems)^49^. The absence of saturation in our material, defined here as the adsorption of less than one O_2_ per cobalt center, was further modeled using a Markov chain simulation (Methods and Supplementary Information Sections 14). In this simplified scenario, addition of O_2_ to a chain of cobalt atoms is disfavored if the gas is already adsorbed at an adjacent site. With this model, the simulated occupancy of cobalt approaches 42%, in agreement with the saturation capacity of the strong binding site at low pressures (∼38%). Thus, negative cooperativity between the open cobalt sites appears integral to the adsorption behavior of O_2_ in Co_2_(OH)_2_(bbta). Negatively cooperative systems display a weakened response to increasing equivalents of ligand, enabling them to maintain a sensitivity to substrate concentration through much greater ranges than experienced in non-cooperative or positively cooperative systems^1–3^. As a result, an adsorbent exhibiting negatively cooperative guest binding would continue to adsorb even at high pressures, leading to significant working capacities over a large range of conditions, as is demonstrated here for Co_2_(OH)_2_(bbta).

## Discussion

The foregoing results show that the framework Co_2_(OH)_2_(bbta) adsorbs O_2_ at low temperatures via reversible reduction to form cobalt(III)-superoxo species. At low pressures, Co_2_(OH)_2_(bbta) exhibits higher capacities and stronger binding of O_2_ than any other reported cobalt(II) metal–organic framework and may therefore be a promising candidate for O_2_-selective air separations. Crucially, the highly basic hydroxo ligands in this material increase the reducing potential of the cobalt(II) sites and also engage in secondary-sphere hydrogen bonding interactions with bound superoxo groups, affording stabilization analogous to that observed in biological O_2_ carriers and biomimetic O_2_-binding molecules^28,29,41,50–52^. Unexpectedly, this allosteric behavior also leads to a negative cooperativity for O_2_ binding, which is unprecedented in an extended framework material, though one previous example involving a molecular complex within a zeolite was found to display a Hill coefficient of 0.51^53^. This behavior can be contrasted to the positive cooperativity observed of CO binding in the isostructural framework Fe_2_Cl_2_(bbta)^9^, demonstrating how precise atomic control in the synthesis and modification of these materials can yield complex and emergent biomimetic properties.

## Methods

### General considerations

The ligand H_2_(bbta) was synthesized according to a previously published procedure^33^. All other reagents were obtained from commercial vendors and used as received. Ultra-high purity grade (99.999% purity, Praxair) He, N_2_, and O_2_ were used for all adsorption, spectroscopy, magnetometry, neutron, and X-ray diffraction measurements. After desolvation, all metal–organic framework samples were stored and handled under an N_2_ atmosphere using standard glove box techniques. Elemental analyses (C, H, and N) were obtained from the Microanalytical Laboratory at the University of California, Berkeley; Co and Cl analyses were obtained from Galbraith Laboratories, Inc. Certain commercial equipment, instruments, or materials are identified in this paper to foster understanding. Such identification does not imply recommendation or endorsement by the National Institute of Standards and Technology, nor does it imply that the materials or equipment identified are necessarily the best available for the purpose.

### Synthesis of Co_2_(OH)_2_(bbta)

Co_2_Cl_2_(bbta) was synthesized and desolvated according to a previously reported procedure^33^. Desolvated Co_2_Cl_2_(bbta) (100.0 mg, 0.3226 mmol) was soaked in a solution of 1 M (M = mol L^−1^) aqueous ammonia (100 mL) at room temperature for 24 h. The suspension was filtered and soaked in aqueous ammonia two more times before being filtered and soaked in water at room temperature for 3 days. The water was replaced every 24 h until the supernatant pH was no longer basic, as tested by pH paper. The suspension was then filtered, rinsed with methanol, and the resulting yellow-orange powder was heated at 160 °C under vacuum for 12 h. Anal. Calcd for Co_2_O_2_C_6_N_6_H_4_ (%): C, 23.25; H, 1.30; N, 27.11; Co, 27.03*; Cl, 0; Cl/Co = 0. Found (%): C, 24.08; H, 1.00; N, 27.38; Co, 26.6(4); Cl, <0.01; Cl/Co = < 0.00037. *For Co_2_(OH)_2_(bbta)·7(H_2_O)

### Gas adsorption

Gas adsorption isotherms for pressures in the range 0–1.2 bar were measured by a volumetric method using a Micromeritics ASAP 2020 instrument. A typical sample of ca. 50 mg of material was transferred to a pre-weighed analysis tube, which was capped with a transeal and evacuated by heating under dynamic vacuum until an outgas rate of less than 2.6 μbar min^−1^ was achieved. The evacuated analysis tube containing the degassed sample was then carefully transferred to an electronic balance and weighed again to determine the mass of sample. The tube was then transferred back to the analysis port of the gas adsorption instrument. The outgas rate was again confirmed to be less than 2.6 μbar min^−1^. For all isotherms, warm and cold free space correction measurements were performed using ultra-high purity He gas; N_2_ isotherms at 77 K were measured in liquid nitrogen baths, using UHP-grade gas sources and all room temperature measurements were measured with an insulated water bath. Oil-free vacuum pumps and oil-free pressure regulators were used for all measurements to prevent contamination of the samples during the evacuation process or of the feed gases during the isotherm measurements. Langmuir surface areas were determined from N_2_ adsorption data at 77 K using Micromeritics software. Gas adsorption isotherms at 195 K were measured using baths with dry ice and isopropanol, and temperatures between −70 and −40 °C were measured using a Julabo immersion cooler with silicone-based Julabo thermal bath fluid. In all measurements, equilibration was determined by a change of less than 0.010% over a minute in the pressure over the sample, which typically involved each measurement point taking half an hour or longer.

### Adsorption isotherm fitting

Adsorption isotherms for the uptake of O_2_ in Co_2_(OH)_2_(bbta) at 195, 213, and 223 K were adequately fit with a dual-site Langmuir equation (Eq. 1), where *n* is the amount adsorbed in mmol g^−1^, *n*_*sat,i*_ is the saturation capacity in mmol g^−1^, *b*_*i*_ is the Langmuir parameter in bar^−1^, and *P* is the pressure in bar.

1 $${\it{n}} = \frac{{{\it{n}}_{{\it{sat}},1}{\it{b}}_1{\it{P}}}}{{1 + {\it{b}}_1{\it{P}}}} + \frac{{{\it{n}}_{{\it{sat}},2}{\it{b}}_2{\it{P}}}}{{1 + {\it{b}}_2{\it{P}}}}$$

2 $$b_i = e^{S_i/R}e^{ - E_i \cdot 1000/RT}$$

The Langmuir parameter can further be expressed using Eq. 2, where *S*_*i*_ is the site-specific integral entropy of adsorption in J mol^−1^K^−1^, *E*_*i*_ is the site-specific differential enthalpy of adsorption in kJ mol^−1^, *R* is the gas constant in J mol^−1^K^−1^, and *T* is the temperature in K. The isotherms were fit independently for each temperature. However, it should be noted that the saturation capacities, *n*_*sat,i*_, were fixed to the values determined from a simultaneous fit to all temperatures. The error for these values was determined using the program Origin and are determined to be 2.46 ± 0.02 and 8.3 ± 0.1 mmol g^−1^ for *n*_*sat,1*_ and *n*_*sat,2*_, respectively.

### Differential enthalpy of adsorption calculations

Using the dual-site and single-site Langmuir fits, the differential enthalpy of adsorption, Δ*h*_ads_, can be calculated as a function of the total amount of gas adsorbed, *n*, by using the Clausius−Clapeyron equation (Eq. 3), where *R* is the gas constant in J mol^−1^K^−1^, *T* is the temperature in K, *n* is the total amount adsorbed in mmol g^−1^, and *P* is the pressure in bar.

3 $${\mathrm{\Delta }}h_{ads} = - RT^2\left( {\frac{{\partial \ln P}}{{\partial T}}} \right)_n$$

The Langmuir fits for O_2_ and N_2_ adsorption in Co_2_(OH)_2_(bbta) and Co_2_Cl_2_(bbta) were used to obtain the exact pressures that correspond to specific loadings at 195, 213, and 223 K with Mathematica 11.0. This was done at loading intervals of 0.05 mmol g^−1^. At each loading, the slope of the best-fit line to ln(*P*) versus 1/*T* was calculated in order to obtain the differential enthalpy of adsorption. The y-intercepts of these linear trendlines are equal to −Δ*s*_ads_ *R*^−1^ at each loading (with *p*_0_ = 1 bar), and thus were used to determine the corresponding differential entropies of adsorption. The results are shown in Supplementary Fig. 14 and reported in Supplementary Tables 1–4.

To corroborate the use of the dual-site Langmuir fit for O_2_ adsorption on Co_2_(OH)_2_(bbta), additional calculations were performed by fitting the O_2_ isotherms independently by spline interpolation and utilizing those fits to determine the differential enthalpy of adsorption, as described above. The differential enthalpy of adsorption obtained through this method is similar within error to the values calculated using the dual-site Langmuir model, providing validation for interpreting the adsorption data with a dual-site Langmuir model (Supplementary Fig. 16).

### Ideal adsorbed solution theory and vacuum swing adsorption calculations

Ideal adsorbed solution theory (IAST)^35^ was used to predict mixed gas behavior (specifically, 0.21 bar O_2_ and 0.79 bar N_2_) from single-component O_2_ and N_2_ adsorption isotherms. IAST selectivities as a function of temperature were calculated using the Langmuir fits for O_2_ and N_2_ in Co_2_(OH)_2_(bbta) and listed in Supplementary Table 5. The selectivity factor, *S*, is defined according to Eq. 4, where *n*_*i*_ is the amount adsorbed for each component as determined from IAST and *x*_*i*_ is the mole fraction of each component in the gas phase at equilibrium.

4 $$S = \frac{{n_{O_2}/n_{N_2}}}{{x_{O_2}/x_{N_2}}}$$

Vacuum swing adsorption capacities were determined by extrapolating the exact capacity of adsorbed O_2_ on Co_2_(OH)_2_(bbta) at 0.05 and 0.21 bar O_2_ pressure at 195 K, by using the Langmuir adsorption fits determined as described above (3.009 and 4.507 mmol g^−1^ respectively). The difference in these values leads to a working capacity of 1.498 mmol g^−1^, higher values than seen in either Co-BTTri or Fe_2_(dobdc)^24^.

### X-ray powder diffraction

Powder X-ray diffraction data for Co_2_Cl_2_(bbta) and Co_2_(OH)_2_(bbta) were collected on Beamline 17-BM-B at the Advanced Photon Source at Argonne National Laboratory. A sample of the fully desolvated framework (∼2 mg) was loaded into a 1.0 mm borosilicate capillary inside a N_2_-atmosphere glovebox, before being attached to a custom-designed valved gas-dosing cell and transferred to the goniometer head. Adsorbed N_2_ was removed by evacuating in situ using a turbomolecular pump. Once diffraction patterns were obtained for the evacuated framework, the samples were dosed with O_2_ (5 to 1000 mbar). Diffraction data were collected after allowing the samples to equilibrate for at least 30 min after each dose, as confirmed by a constant pressure readout and consistent diffraction patterns. An Oxford Systems Cryostream 800 was used to change the temperature between 100 and 298 K. All X-ray wavelengths were between 0.451 and 0.453 Å, and are specified for each experiment. Diffracted intensity was recorded by a PerkinElmer a-Si flat panel detector. In the case of Co_2_Cl_2_(bbta), the sample was dosed with 27 mbar of O_2_ at 298 K and then slowly cooled to 110 K (2 K/min), while monitoring powder X-ray diffraction patterns during the temperature changes.

### X-ray powder diffraction analysis

Precise unit cell parameters of evacuated Co_2_Cl_2_(bbta), O_2_-dosed Co_2_Cl_2_(bbta), evacuated Co_2_(OH)_2_(bbta), and O_2_-dosed Co_2_(OH)_2_(bbta) were obtained by structureless Pawley refinement, as implemented in TOPAS-Academic 4.1, and all were consistent with a hexagonal lattice, similar to those observed for Fe_2_Cl_2_(bbta) and other M_2_X_2_(bbta) frameworks^9,32,33,54^. The backgrounds of the patterns were modeled with Chebyshev polynomial functions that were used, along with the correct unit cell parameters, sample displacement and profile parameters, in the subsequent Rietveld refinements. Peak shapes were described with the fundamental parameters approach.

The structural model for evacuated Fe_2_Cl_2_(bbta) was first used as a starting point for evacuated Co_2_Cl_2_(bbta)^9^. The positions and thermal displacement parameters of the cobalt and chloride atoms were freely refined, and the carbon and nitrogen atoms of the bbta^2−^ ligand were refined with soft constraints. A single refined thermal displacement parameter was assigned to all atoms of the bbta^2−^ ligand. Consistent with previous work as well as the needle-like crystal habit observed in the SEM images of Co_2_Cl_2_(bbta) (Supplementary Fig. 45), a single March-Dollase correction along the (0 0 1) direction provided an improvement in the goodness-of-fit parameters (*R*_*wp*_ improved by ∼1.3%). The Fourier difference map showed little evidence for electron density over the framework metal centers, indicating that the material was fully desolvated prior to the gas dosing experiments.

The Rietveld refinement using O_2_-dosed Co_2_Cl_2_(bbta) data obtained at 110 K was approached by examining the influence of O_2_-dosing on the diffraction pattern of the parent framework. Only a slight unit cell contraction was observed at this temperature (<0.9%), with some slight intensity changes being the chief difference between the two patterns. The Fourier difference map showed evidence for O_2_ adsorbed within the pores, and a species was modeled along the locations indicated by the map and then allowed to freely refine. Upon this refinement, the closest distance of O_2_ from the cobalt ions is ∼2.7 Å, with few changes observed in the coordination environment of the cobalt ions otherwise. This distance suggests a weak interaction, consistent with the small differential enthalpy of adsorption of O_2_ measured for this framework. As with the desolvated sample, a single March-Dollase correction applied along the (0 0 1) direction provided an improvement in the goodness-of-fit parameters (*R*_*wp*_ improved by 1.06%).

The obtained structural model of desolvated Co_2_Cl_2_(bbta) was used as the starting point for the Rietveld refinement using the diffraction pattern of evacuated Co_2_(OH)_2_(bbta). The hydroxo oxygen atom and cobalt atom were freely refined, and the ligand atom positions were refined within soft restraints. A single refined thermal displacement parameter was assigned to all atoms of the bbta^2−^ ligand. In the course of the refinement, it should be noted that the combination of the special positions of the Co and O atoms and the shorter Co–O bond length (∼2.1 Å in comparison to the ∼2.4 Å Co–Cl bond distances observed in Co_2_Cl_2_(bbta)) cause the O–Co–O angle to approach 170°, rather than 180° as observed in Co_2_Cl_2_(bbta). As a result, the coordination environment of the cobalt centers deviates slightly from an ideal square pyramidal geometry. However, this geometry is also observed in the Rietveld refinements performed using the neutron powder diffraction data, and is not chemically unreasonable, particularly if some strain is imposed by the crystallographic lattice. The O–Co–O angle also appears to play a role in the unit cell volume of Co_2_(OH)_2_(bbta) being slightly bigger than that of Co_2_Cl_2_(bbta), despite Cl^−^ having a larger ionic radius than OH^−^. As with Co_2_Cl_2_(bbta), single March-Dollase correction was applied along the (0 0 1) direction and provided a slight improvement in the goodness-of-fit parameters (*R*_*wp*_ improved by 0.3%), consistent with the needle-like crystal habit observed in the SEM images of Co_2_(OH)_2_(bbta). Additionally, there was little to no observable electron density over the metal center, indicating complete desolvation prior to the gas dosing experiments on the same sample.

The structural model for the evacuated sample was used as the starting point for the determination of the structural models for Co_2_(OH)_2_(bbta) dosed with increasing pressures of O_2_ (0.0067–1.01 bar). Upon gas dosing, significant unit cell contractions were observed (4427(3) Å^3^ to 4047.4(5) Å^3^), necessitating care in the refinement of the structural models from the evacuated material, as the starting point was quite far off. Soft constraints on the bbta^2−^ ligand bond lengths and angles were employed, and the cobalt and hydroxo oxygen atoms were refined within constraints. After the framework structure was approximately determined, a Fourier difference map was used to examine adsorbed species within the pores. From this map, a bent shape of unmodeled electron density could be distinguished above the metal centers of the framework, reminiscent of a disordered bent end-on superoxo species. When oxygen atoms were assigned to be within this unmodeled density and then freely refined, one atom appeared to be directly above the cobalt ion on the two-fold axis that runs through the metal center. The second atom, which refined to a position ∼1.3 Å away from the first, refined to a general position with approximately 0.5 occupancy of the first atom in a position close to the hydroxo oxygen atom (∼2.7–2.9 Å). At low pressures of O_2_, the species over the metal centers appeared to be the only significant adsorbed species. At higher dosed pressures of O_2_, additional electron density could be resolved in the pores. Specifically, a position in between the superoxo species and over the ligand appeared to be a preferred location for a physisorbed oxygen. However, this position appears to be highly disordered under the measured conditions, in contrast to structural models obtained from the powder neutron diffraction data described below. As with desolvated Co_2_(OH)_2_(bbta), a single March-Dollase correction applied along the (0 0 1) direction provided an improvement in the goodness-of-fit parameters (*R*_*wp*_ improved by 0.4%).

In all structural models, hydrogen atoms were added to the benzene ring of bbta^2−^ ligand to yield a C–H distance of 1.09 Å. During the final refinement, all parameters were freely refined. The result of all Rietveld refinements using data collected on Co_2_Cl_2_(bbta) are reported in Supplementary Figs. 21 and 22, and the parameters are listed in Supplementary Tables 6–8. The result of all Rietveld refinements using powder X-ray diffraction data collected on the Co_2_(OH)_2_(bbta) framework are reported in Supplementary Tables 10–12, and the refinements are shown in Supplementary Figs. 26–35.

We also collected powder X-ray diffraction data for desolvated Co_2_(OH)_2_(bbta) prepared according to a previously published procedure, via washing of Co_2_Cl_2_(bbta) with aqueous KOH^1^. The sample was washed as previously described, before being fully desolvated (activation conditions: 100 °C for 24 h) and packed into a 1.0 mm borosilicate capillary inside a nitrogen glovebox that was flame-sealed. The sample was then measured at Beamline 12.2.2 at the Advanced Light Source of Lawrence Berkeley National Laboratory under an average wavelength of 0.4948 Å at room temperature (see Supplementary Fig. 19). The diffraction data confirms that in our hands, the known preparation resulted in synthesis of a significant amount of Co(OH)_2_ alongside Co_2_(OH)_2_(bbta), in contrast to the preparation using aqueous ammonia washes.

### Neutron powder diffraction collection and analysis

Powder neutron diffraction data were collected on 0.485 g of desolvated Co_2_(OH)_2_(bbta), loaded into a vanadium sample can in a helium glovebox. The sample can was sealed with an indium o-ring onto a copper heating block containing a valved outlet for gas loading. Data were collected at the high-resolution powder diffractometer, BT1, at the National Institute of Standards and Technology Center for Neutron Research, utilizing a Ge(311) monochromator with an in-pile 60’ collimator, corresponding to a neutron wavelength of 2.0772 Å. After mounting the sample to a bottom-loaded closed cycle refrigerator, the sample was cooled to 6 K for measurement, and subsequent measurements were collected at 150 and 300 K (Supplementary Fig. 33). For gas loading, the sample was dosed with ∼1 equiv. of O_2_ per cobalt at 100 K, with adsorption verified barometrically prior to cooling to 8 K to avoid condensation. Measurements were then conducted at 8, 150, and 300 K, then at 150 and 8 K. Diffraction patterns for initial and final 8 and 150 K data sets were virtually identical (Supplementary Fig. 35).

Powder neutron diffraction data was analyzed using GSAS^55^. Initially, Le Bail refinements were conducted to determine the profile parameters, after which Rietveld refinements were conducted. Bond restraints were initially placed on all bonds, based on chemical information. Final refinements only included bond restraints on the phenyl ring, with minimal impact. Occupancies that refined within unity (the atoms of bbta^2−^ linker, the Co atom, and the O atom of the OH^−^ groups), were fixed. The thermal parameters of the bbta^2−^ linker were constrained to the same value. During the final refinements, the profile parameters, all atomic positions, and the occupancies were all refined, for both the gas-dosed and bare structures. The parameters are listed in Supplementary Tables 13–16, and the refinements are shown in Supplementary Figs. 34, 36, and 37. To confirm the position of the hydrogen atom of the hydroxo groups, a Rietveld refinement was performed where the hydroxo hydrogen atom was omitted from the structure, while keeping all other aspects of the refinement constant, and a Fourier difference map (*F*_*obs*_ – *F*_*calc*_) was calculated from the refinement. The diminished goodness-of-fit parameters and the clear presence of well-localized atoms in the negative component of the different map (Supplementary Figs. 38 and 39) confirm the assignment of the hydrogen atom and its ability to hydrogen bond to the superoxo oxygen atom(d_H···O_ = 2.35(2) Å).

### Infrared spectroscopy

Infrared spectra were collected using a Bruker Vertex 70 spectrometer equipped with a glowbar source, KBr beamsplitter, and a liquid nitrogen cooled mercury-cadmium-telluride detector. A custom-built diffuse reflectance system with an IR-accessible gas dosing cell was used for all measurements. Sample temperature was controlled by an Oxford Instruments OptistatDry TLEX cryostat, and sample atmosphere was controlled by a Micromeritics ASAP 2020Plus gas sorption analyzer. In a typical experiment, a sample of the desolvated framework material was dispersed in dry KBr (10 wt. %) by gently mixing the two powders in an argon-filled glovebox and was subsequently evacuated at room temperature overnight. Spectra were collected in situ under UHP-grade ^16^O_2_ and ^18^O_2_ (99 atom % ^18^O, Sigma-Aldrich) at 4 cm^−1^ resolution until equilibrium was observed in both pressure output and spectra. Peak fitting was performed in Origin using 2–4 Lorentzian peaks with the Levenberg-Marquardt algorithm. Spectra representative of various coverages with different superoxo species are shown (Supplementary Figs. 58–62).

### Hill equation analysis

The occupancy values of the superoxo species, as determined by powder X-ray diffraction analysis, were fit to the Hill equation (Eq. 5), in which *θ* is the occupancy (between 0 and 1), [*L*] is the pressure of O_2_ in bar, *K*_*d*_ is the apparent dissociation constant, and *n* is the Hill coefficient, which can be approximately related to the number of binding sites^49^. It should be noted that we were unable to determine experimentally a physically-meaningful value for *K*_*d*_, so the parameter was allowed to freely refine in the process of all fits.

5 $$\theta = \frac{{[L]^n}}{{K_d + [L]^n}}$$

Additionally, in order to determine whether the adsorption of O_2_ on the cobalt centers could be modeled to the Hill equation, the occupancies determined by X-ray powder diffraction were compared to the gas adsorption data. Stoichiometry, as well as X-ray powder diffraction analysis (detailed above) of the occupancy of reduced oxygen species within the pores of Co_2_(OH)_2_(bbta), reveal that the gas adsorption data encompasses both adsorption at coordinatively-unsaturated cobalt centers as well as physisorption. Low pressure adsorption data up to an unspecified regime should correspond to adsorption on isolated cobalt centers, whereas the occupancies determined from X-ray powder diffraction data could provide information about adsorption on the metal centers at high pressures, otherwise obscured by the large amount of physisorption in the gas adsorption data. In the X-ray powder diffraction data, in both the 7 mbar O_2_-dosed and 50 mbar O_2_-dosed Rietveld refinements, there is little evidence for occupancy of the physisorption site. These results indicate that the gas adsorption data up to at least 50 mbar is largely representative of metal-site adsorption. As a result of this assessment, the adsorption isotherm for O_2_ in Co_2_(OH)_2_(bbta) at 195 K was fit between 0 bar and 0.05 bar with the Hill equation. It should be noted that the projected capacity values from the occupancies are slightly higher than the experimental gas adsorption capacities at low pressures, which may be due to defects (e.g. surface sites) that are not reflected in the occupancies.

The fitting of the gas adsorption data for Co_2_Cl_2_(bbta) was also performed. In this instance, all values were unconstrained. We note, however, that when we constrain *q*_sat_ to be the value expected for one O_2_ per Co (5.77 mmol g^−1^), *n* and *K*_*d*_ refine to be 1.30 and 0.85 respectively. These values result in greater deviations from the experimental points than the unconstrained values, and were ultimately disregarded, particularly as we could not justify a reason why n should be greater than 1. Rather, the saturation capacity of 16.9 mmol g^−1^ suggests that the unconstrained fitting might be reflective of pore-filling of the framework with O_2_, with 16.9 mmol g^−1^ being closer to the adsorption values that would be expected with an O_2_ surface area at 90 K, rather than an interaction with the open metal site. This result is consistent with the X-ray powder diffraction and gas adsorption data that indicate a very weak interaction with O_2_.

The residual sum of squares (“RSS”) and the average relative error (“ARE”) are reported with each associated fit.

### Measurements of d.c. magnetic susceptibility

Samples were prepared by adding crystalline powder to a 5-mm i.d. 7-mm o.d. quartz tube containing a raised quartz platform. These samples include desolvated Co_2_Cl_2_(bbta) (9.8 mg), desolvated Co_2_(OH)_2_(bbta) (17.2 mg), methanol-solvated Co_2_(OH)_2_(bbta) (12.8 mg), Co_2_(OH)_2_(bbta) dosed with 0.5 equivalents of O_2_ per cobalt (15.4 mg), and Co_2_(OH)_2_(bbta) dosed with 1 equivalent of O_2_ per cobalt (11.4 mg). Sample powders were restrained with a plug of compacted glass wool that prevented crystallite torqueing, but enabled gas-dosing with O_2_. The methanol-solvated sample was filtered on a polypropylene membrane filter (0.45 μm) and dried briefly at room temperature under vacuum, before being loaded into the quartz tube. Air-free elemental analysis of the sample was measured on the same day of sample preparation in order to accurately quantify the amount of solvent adsorbed in the pores of the material. The O_2_-loaded samples were prepared by attaching the quartz tubes containing activated Co_2_(OH)_2_(bbta) to a Micromeritics ASAP 2020 gas adsorption analyzer. One equivalent of O_2_ and 0.5 equivalents of O_2_ for every Co atom within the structure was calculated and then exposed to the samples. All quartz tubes containing samples, including those loaded with O_2_ gas, were flame-sealed while the sample was cooled with liquid nitrogen. Magnetic susceptibility measurements were performed using a Quantum Design MPMS2 SQUID magnetometer. Measurements of d.c. magnetic susceptibility were collected in the temperature range 2–300 K under applied magnetic fields of 1 T. Diamagnetic corrections were applied to the data using Pascal’s constants to give *χ*_D_ = −0.00014552 emu mol^−1^ for desolvated Co_2_Cl_2_(bbta), *χ*_D_ = −0.00011752 emu mol^−1^ for desolvated Co_2_(OH)_2_(bbta), *χ*_D_ = −0.00019341 emu mol^−1^ for methanol-solvated Co_2_(OH)_2_(bbta), *χ*_D_ = −0.00012672 emu mol^−1^ for Co_2_(OH)_2_(bbta) dosed with 0.5 equivalents of O_2_ per cobalt, and *χ*_D_ = −0.00013592 emu mol^−1^ for Co_2_(OH)_2_(bbta) dosed with 1 equivalent of O_2_ per cobalt. The collected data is shown in Supplementary Figs. 63–65.

### Theoretical calculations

Periodic models for the desolvated and O_2_-loaded Co_2_Cl_2_(bbta) and Co_2_(OH)_2_(bbta) were optimized using the PBE-D3 functional^56,57^, as implemented in the VASP 5.4.4 program^58^. Dioxygen adsorption energies were obtained by single point calculations using the PBE0-D3 functional^59^ with the PBE-D3 optimized structures. The vibrational properties were modeled using tetracobalt clusters, excised from the PBE-D3 optimized structures along a single helical chain. Constrained geometry optimizations were performed using the M06^60^ functional, where the dioxygen molecules, cobalt atoms, and the first coordination sphere of the two central cobalt atoms were allowed to relax. The electronic structure of the frameworks was evaluated using complete active space self-consistent field (CASSCF) single point calculations followed by second-order perturbation theory (CASPT2) using the MOLCAS 8.2 program^61^. An additional set of tetracobalt cluster models was employed, obtained from the experimental crystal structures of desolvated Co_2_Cl_2_(bbta) and Co_2_(OH)_2_(bbta). Dicobalt cluster models were obtained by replacing the external cobalt atoms by magnesium ions. Partial geometry optimizations were performed using the M06 functional by relaxing only C and H atoms. The computational details are further provided in the Supplementary Information.

### Markov chain simulations of negative cooperativity

The framework metal sites were simulated, in a simplified fashion, as a chain of twelve Co atoms whose occupancies (bound or unbound to oxygen) at a given time were modeled as a Markov chain. At each time step, a random Co atom was chosen. If bound, the states of the Co atoms remained unchanged, reflecting the negligible probability of a bound Co atom becoming unbound in the timescales observed. If unbound, the chosen Co atom was changed to a bound state with a probability that decreased with the number of bound neighboring Co atoms, reflecting negative cooperativity. Periodic boundary conditions, simulating the repetition of such short chains in the material, were used to determine the neighbors of the first and last Co atoms. The Markov chain was run to T = 200 timesteps; the probability of an unbound Co atom becoming bound at any timestep given zero, one, and two bound neighbors was 0.5, 0.0005, and 0.0001, respectively. This data is shown in Supplementary Fig. 68.

## Supplementary information

Supplementary Information

## Data Availability

The main data supporting the findings of this study are available within the paper and its Supplementary Information. The source data underlying Figs. 3 and 6 are provided as a Source Data file. Other data are available from the corresponding author upon request. Metrical data for the solid-state structures of Co_2_Cl_2_(bbta), O_2_-dosed Co_2_Cl_2_(bbta), Co_2_(OH)_2_(bbta) and O_2_-dosed Co_2_(OH)_2_(bbta) are available free of charge from the Cambridge Crystallographic Data Centre under reference numbers 1965641–1965647.

## Source data

Source Data
