# Supplementary material for: Negative cooperativity upon hydrogen bond-stabilized O2 adsorption in a redox-active metal–organic framework
Source: Nat Commun. 2020 Jun 18;11:3087. doi: 10.1038/s41467-020-16897-z (PMC7303157; doi:10.1038/s41467-020-16897-z)
Supplement: Supplementary file 1 — Supplementary Information [file 41467_2020_16897_MOESM1_ESM.pdf]

Negative cooperativity upon hydrogen bond-stabilized O_2 adsorption in a redox-active metal–organic framework

Julia Oktawiec,[1] Henry Z. H. Jiang,[1] Jenny G. Vitillo,[2] Douglas A. Reed,[1] Lucy E. Darago,[1] Benjamin A. Trump,[3] Varinia Bernales,[2] Harriet Li,[4] Kristen A. Colwell,[5] Hiroyasu Furukawa,[1,6] Craig M. Brown,[3,7] Laura Gagliardi,[2] Jeffrey R. Long[1,5,6]

[1]*Department of Chemistry, University of California, Berkeley, California, 94720, USA*

[2]*Department of Chemistry and Supercomputing Institute, University of Minnesota, Minneapolis, Minnesota 55455, USA*

[3]*National Institute of Standards and Technology, Center for Neutron Research, Gaithersburg, Maryland 20899, USA*

[4]*Department of Aeronautics and Astronautics, Massachusetts Institute of Technology, Cambridge, Boston, 02139, USA*

[5]*Department of Chemical and Biomolecular Engineering, University of California, Berkeley, California, 94720, USA*

[6]*Materials Sciences Division, Lawrence Berkeley National Laboratory, Berkeley, California, 94720, USA*

[7]*Chemical and Biomolecular Engineering, University of Delaware, Newark, Delaware 19716, USA*

Table of Contents

1. **Thermal gravimetric analysis** 3
2. **Gas adsorption measurements** 3
3. **Adsorption isotherm fitting** 7
4. **Differential enthalpy of adsorption calculations** 10
5. **Ideal adsorbed solution theory calculations** 12
6. **Synchrotron powder X-ray diffraction data and analysis** 13
7. **Powder neutron diffraction data collection and analysis** 24
8. **Hill equation analysis** 31
9. **Scanning electron microscopy** 33
10. **Diffuse reflectance infrared Fourier transform spectroscopy** 34
11. **Measurements of d.c. magnetic susceptibility** 44
12. **Supplementary Methods: Periodic calculations** 46
13. **Supplementary Methods: Cluster calculations** 46
14. **Markov chain simulations of negative cooperativity** 48
15. **Supplementary Methods: Computed electronic and magnetic properties of Co_2Cl_2(bbta) and $Co_2(OH)_2$(bbta)** 49
16. **Supplementary Methods: Periodic models for O_2 adsorbed on Co_2Cl_2(bbta) and $Co_2(OH)_2$(bbta)** 65
17. **Supplementary Methods: Co_4OH and Co_4Cl cluster models for O_2 adsorbed on Co_2Cl_2(bbta) and $Co_2(OH)_2$(bbta)** 74
18. **Supplementary Discussion: Summary of quantum chemical computations** 80
19. **Supplementary Note 1. Checkcif alerts and responses** 82
20. **Supplementary References** 87

1. Thermal gravimetric analysis

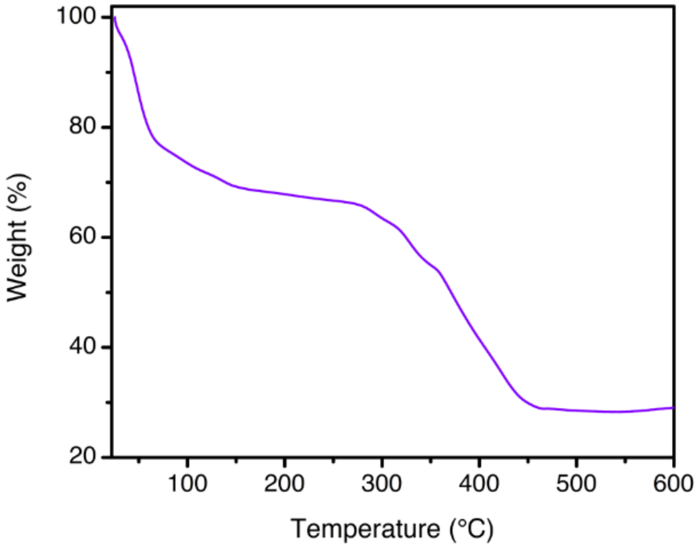

Supplementary Figure 1. Thermal gravimetric analysis of methanol-solvated $Co_2(OH)_2$(bbta) under flowing nitrogen (10 mL/min), with a ramp rate of 2 °C/min. The first initial mass loss at 50 ºC represents loss of solvent in the pores of the framework, and the second large mass loss starting from 300 ºC represents decomposition of the material.

2. Gas adsorption measurements

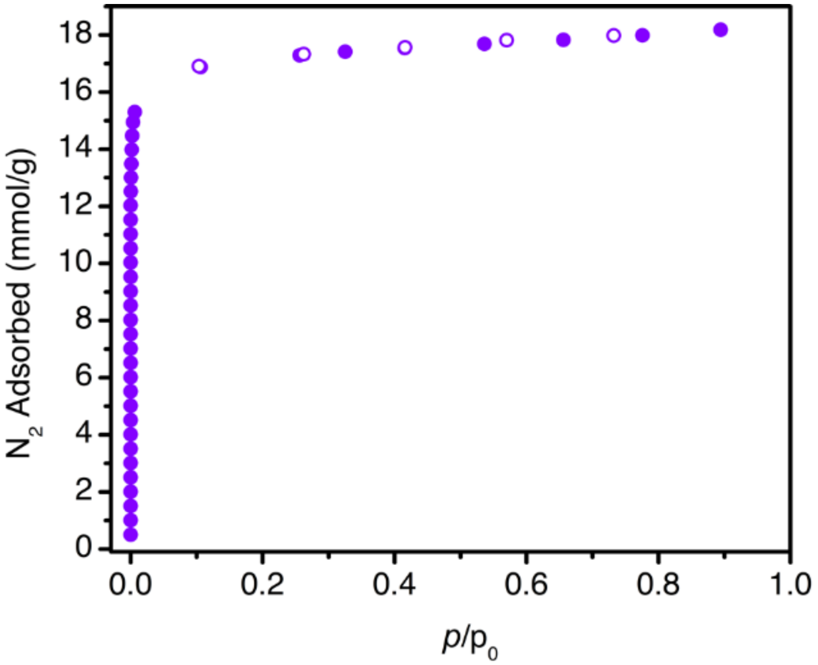

Supplementary Figure 2. N_2 adsorption isotherm of $Co_2(OH)_2$(bbta) (purple symbols) obtained at 77 K. Filled purple circles represent an adsorption branch, and open purple circles represent a desorption branch.

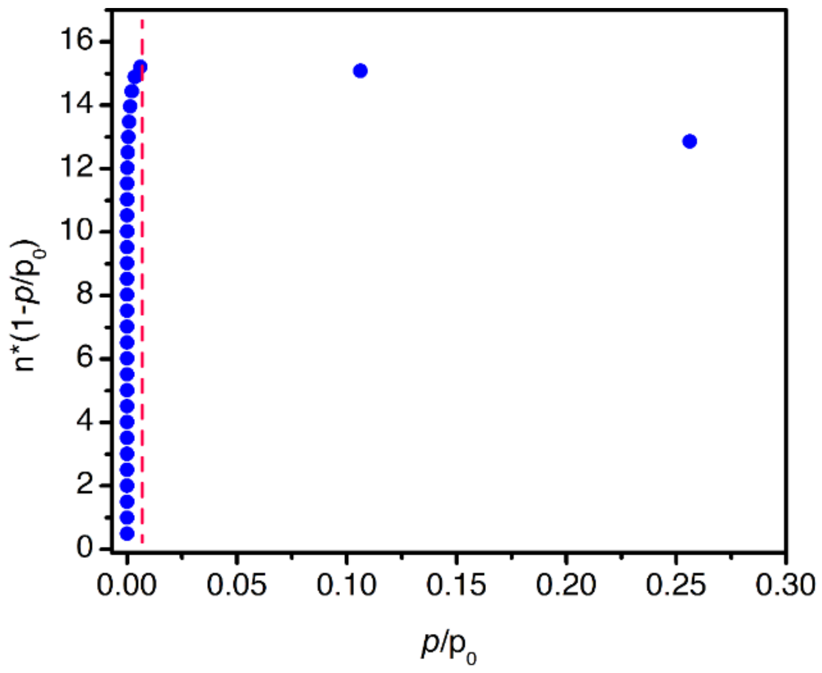

Supplementary Figure 3. A graph of n*(1-p/p_0) vs. p/p_0, to determine the maximum value of p/p_0 to be used to fit the BET isotherm of $Co_2(OH)_2$(bbta), according to the first BET consistency criterion. The maximum value is indicated by the dashed red line.[1]

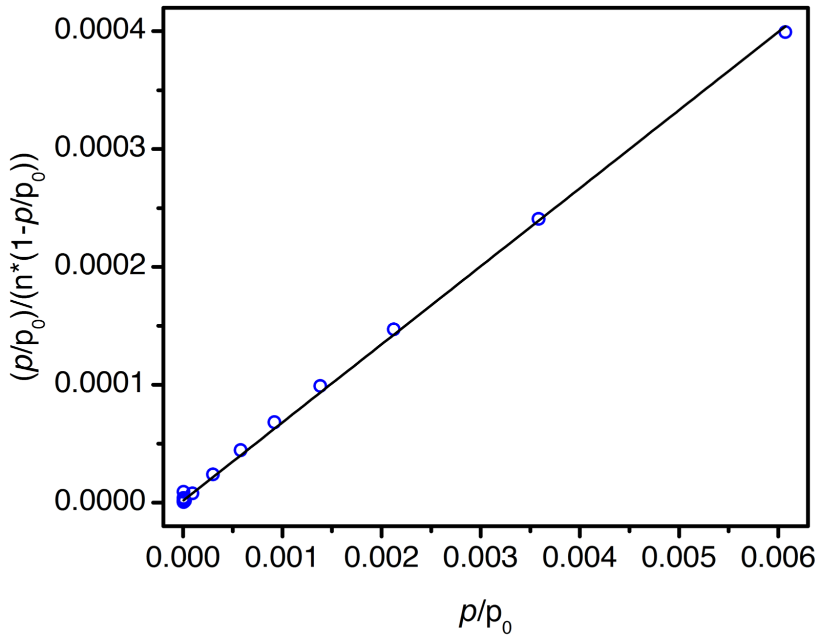

Supplementary Figure 4. A plot of (p/p_0)/(n*(1−p/p_0)) vs. p/p_0 to determine the BET surface area of $Co_2(OH)_2$(bbta). Given that the y-intercept calculated from the best fit line is a positive value, the second BET consistency criterion is fulfilled to yield a BET surface area of 1472(9) m^2/g.[1] The Langmuir surface area is 1790 m^2/g.

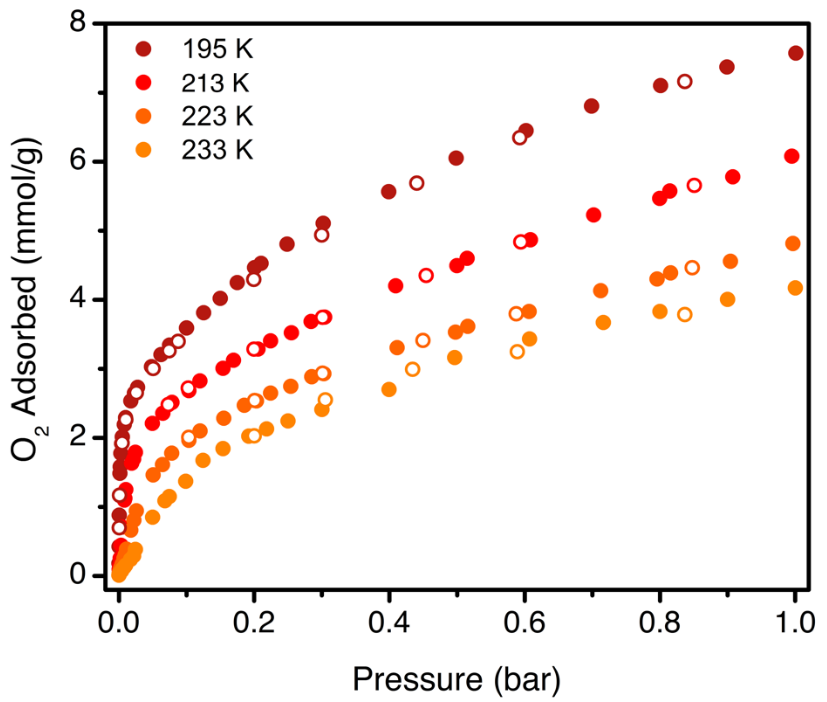

Supplementary Figure 5. Adsorption isotherms of O_2 obtained for $Co_2(OH)_2$(bbta) at 195, 213, 223, and 233 K (dark red, red, dark orange, and orange symbols, respectively). The filled dot symbols represent adsorption and the open circles represent desorption.

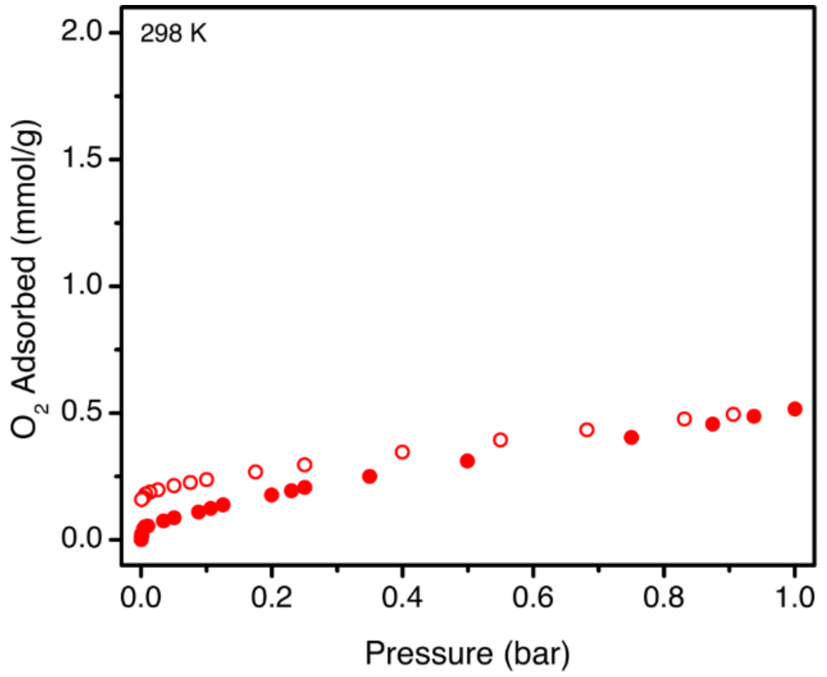

Supplementary Figure 6. Adsorption isotherm of O_2 obtained for $Co_2(OH)_2$(bbta) at 298 K (red symbols). The filled dot symbols represent adsorption points and the open circles represent desorption. The slight hysteresis observed is likely due to the error of the extremely low uptake observed in these conditions.

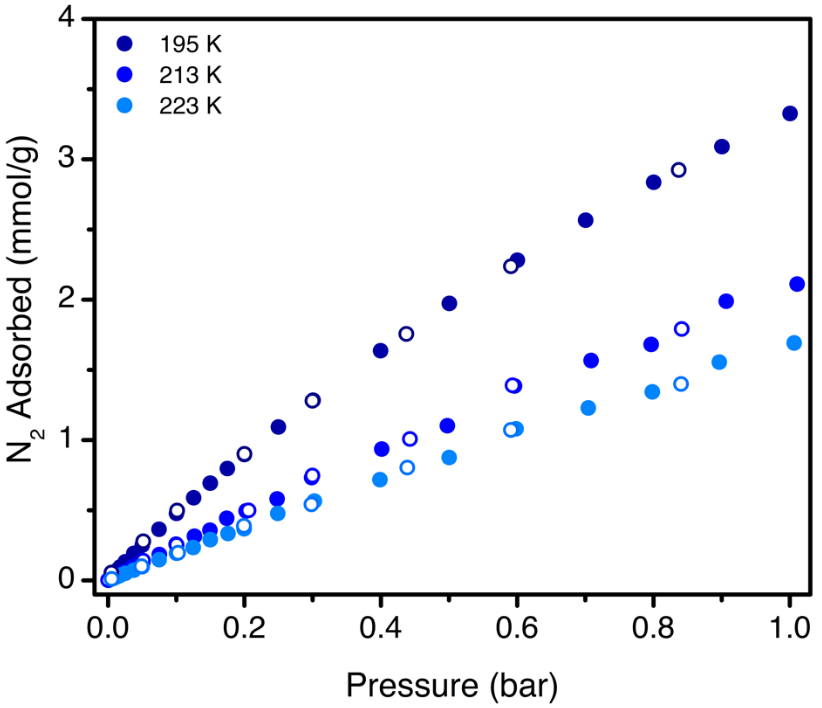

Supplementary Figure 7. Adsorption isotherms of N_2 obtained for $Co_2(OH)_2$(bbta) at 195, 213, and 223 K (dark blue, blue, and light blue symbols, respectively). The filled dot symbols represent adsorption and the open circles represent desorption.

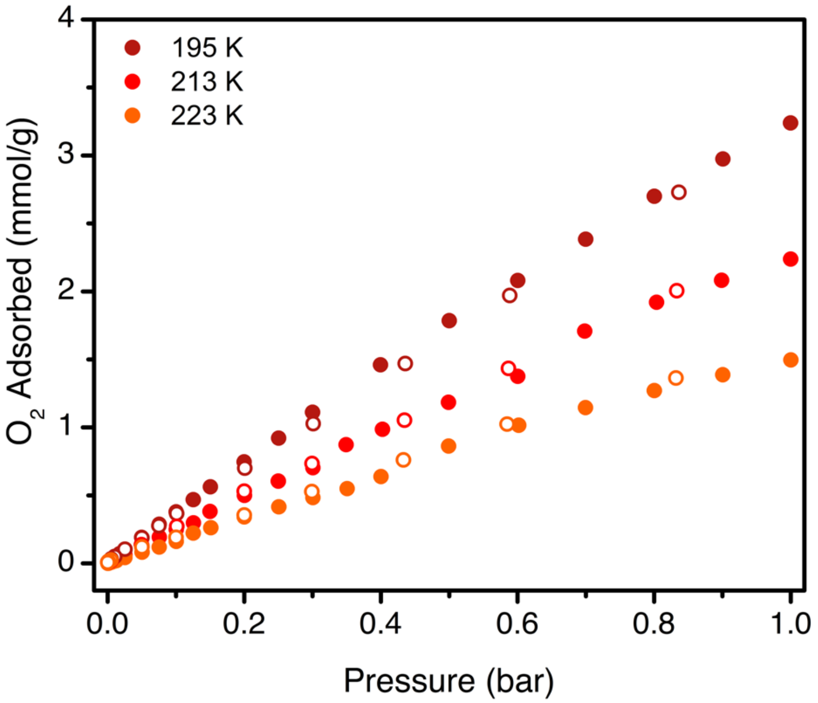

Supplementary Figure 8. Adsorption isotherms of O_2 obtained for Co_2Cl_2(bbta) at 195, 213, and 223 K (dark red, red, and orange symbols, respectively). The filled dot symbols represent adsorption and the open circles represent desorption.

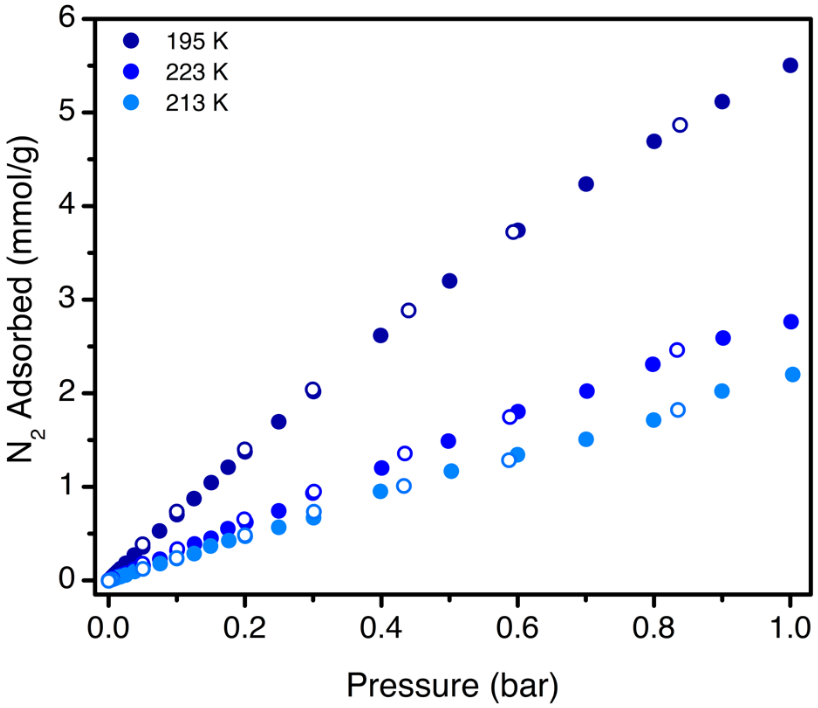

Supplementary Figure 9. Adsorption isotherms of N_2 obtained for Co_2Cl_2(bbta) at 195, 213, and 223 K (dark blue, blue, and light blue symbols, respectively). The filled dot symbols represent adsorption and the open circles represent desorption.

3. Adsorption isotherm fitting

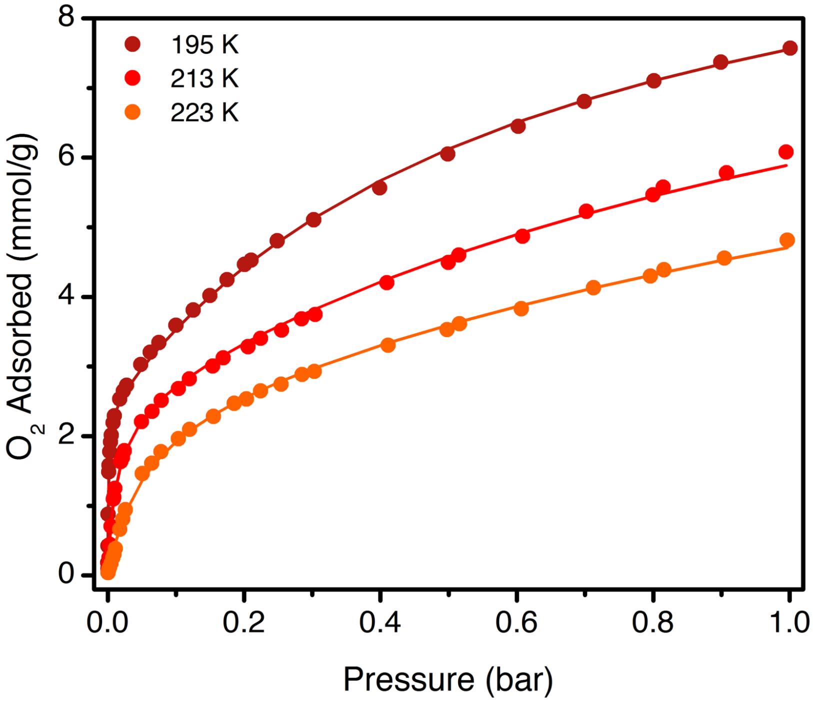

Supplementary Figure 10. Dual-site Langmuir fits (solid lines) of O_2 adsorption isotherms for $Co_2(OH)_2$(bbta) collected at 195, 213 , and 223 K (dark red, red, and orange symbols, respectively).

The fits were used to determine the differential enthalpy and entropy of O_2 adsorption in the material.

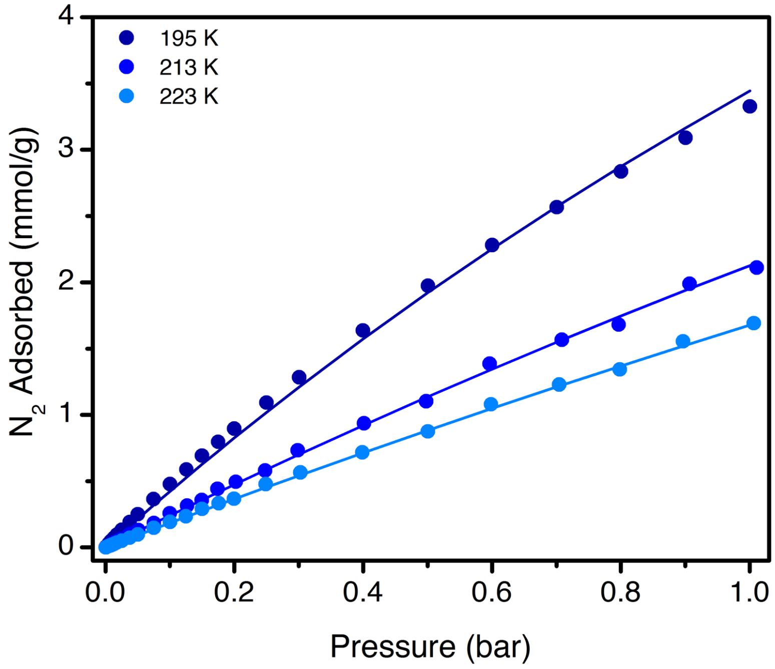

Supplementary Figure 11. Single-site Langmuir fits (solid lines) of N_2 adsorption isotherms for $Co_2(OH)_2$(bbta) collected at 195, 213, and 223 K (navy blue, bright blue, and cerulean symbols respectively). The fits were used to determine the differential enthalpy and entropy of nitrogen adsorption.

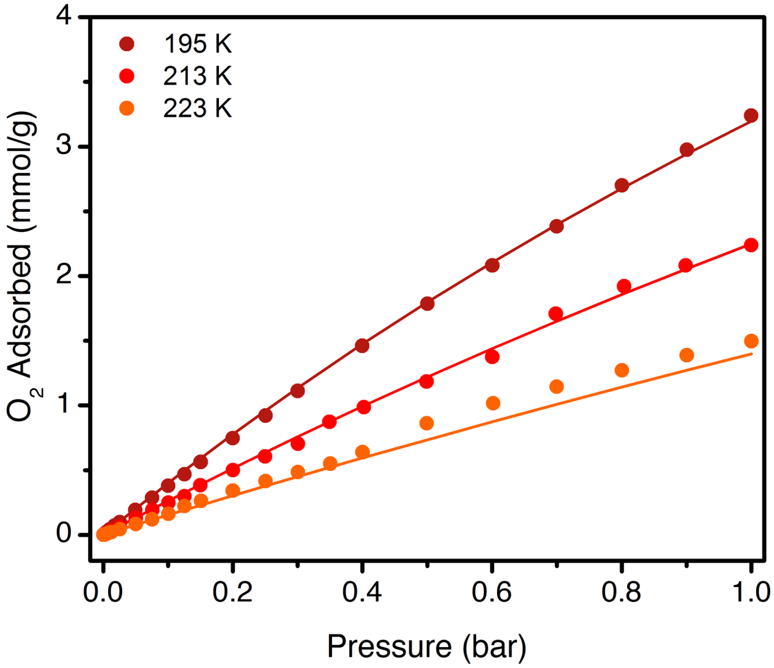

Supplementary Figure 12. Single-site Langmuir fits (solid lines) of O_2 adsorption isotherms for Co_2Cl_2(bbta) collected at 195, 213, and 223 K (dark red, red, and orange symbols respectively). The fits were used to determine the differential enthalpy and entropy of nitrogen adsorption.

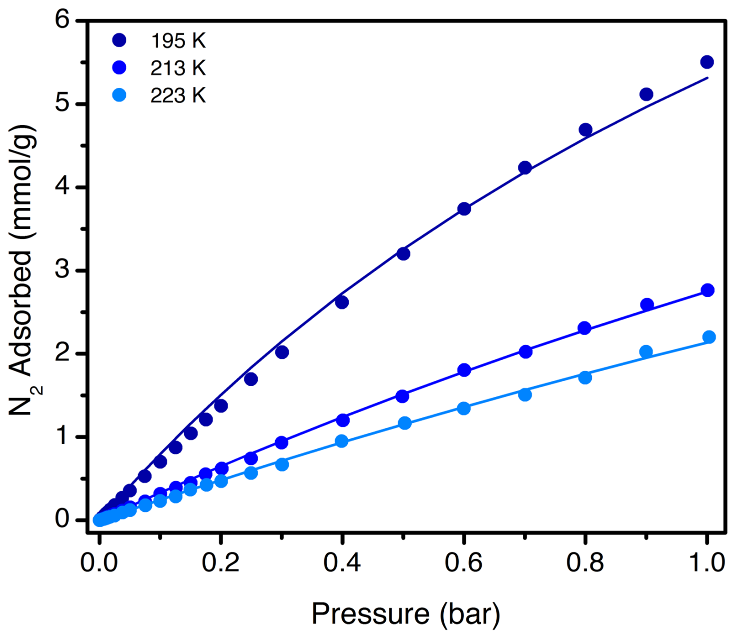

Supplementary Figure 13. Single-site Langmuir fits (solid lines) of N_2 adsorption isotherms for Co_2Cl_2(bbta) (symbols) collected at 195, 213, and 223 K. The Langmuir fits were used to determine the differential enthalpy and entropy of nitrogen adsorption.

Supplementary Table 1. Dual-site Langmuir fit parameters for O_2 adsorption isotherms of $Co_2(OH)_2$(bbta). As described above, the saturation capacities for the first and second sites were determined by a simultaneous fit to all isotherms, and then fixed for each individual refinement.

Parameter	195 K	213 K	223 K
$n_{sat,1}$ (mmol/g)	2.457	2.457	2.457
$-S_1$ (J/mol·K)	199.004	203.045	200.193
$-E_1$ (kJ/mol)	49.836	50.982	50.082
$n_{sat,2}$ (mmol/g)	8.332	8.332	8.332
$-S_2$ (J/mol·K)	79.640	81.037	81.635
$-E_2$ (kJ/mol)	16.258	16.651	16.495

Supplementary Table 2. Single-site Langmuir fit parameters for N_2 adsorption isotherms of $Co_2(OH)_2$(bbta). As described above, the saturation capacity was adapted from the 77 K Langmuir surface area, which was fixed for all fits while the E_1 and S_1 parameters were allowed to vary.

Parameter	195 K	213 K	223 K
$n_{sat,1}$ (mmol/g)	16.30	16.30	16.30
$-S_1$ (J/mol·K)	7.153	7.267	7.235
$-E_1$ (kJ/mol)	9.460	9.510	9.418

Supplementary Table 3. Single-site Langmuir fit parameters for O_2 adsorption isotherms of Co_2Cl_2(bbta). As described above, the saturation capacity was adapted from the 77 K Langmuir surface area, which was fixed for all fits while the E_1 and S_1 parameters were allowed to vary.

Parameter	195 K	213 K	223 K
$n_{sat,1}$ (mmol/g)	14.40	14.40	14.40
$-S_1$ (J/mol·K)	57.475	61.440	63.053
$-E_1$ (kJ/mol)	9.176	10.100	9.928

Supplementary Table 4. Single-site Langmuir fit parameters for N_2 adsorption isotherms of Co_2Cl_2(bbta). As described above, the saturation capacity was adapted from the 77 K Langmuir surface area, which was fixed for all fits while the E_1 and S_1 parameters were allowed to vary.

Parameter	195 K	213 K	223 K
$n_{sat,1}$ (mmol/g)	14.40	14.40	14.40
$-S_1$ (J/mol·K)	9.925	10.107	10.242
$-E_1$ (kJ/mol)	15.223	15.340	15.750

4. Differential enthalpy of adsorption calculations

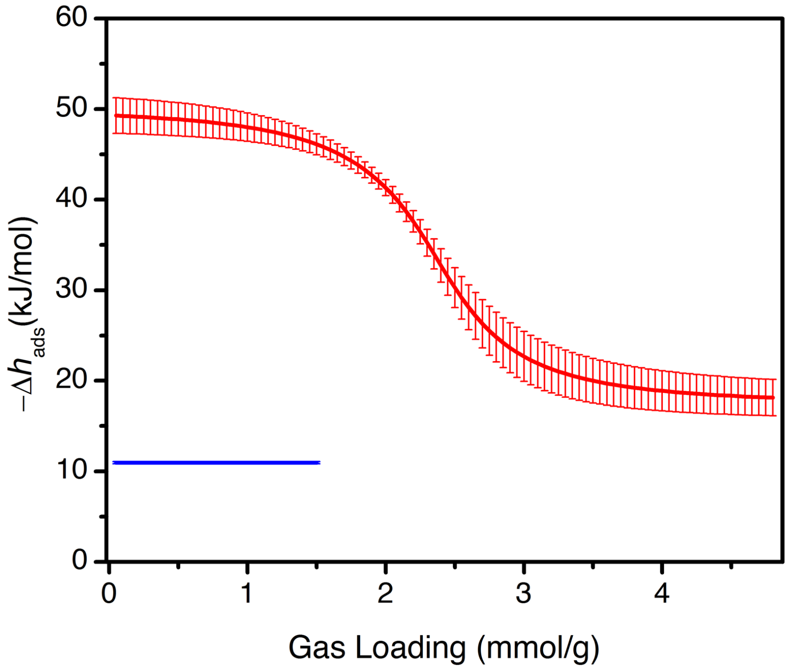

Supplementary Figure 14. Differential enthalpy of O_2 adsorption (red line) and differential enthalpy of N_2 adsorption (blue line) in $Co_2(OH)_2$(bbta), determined using the Clausius–Clapeyron equation and the fit values shown in Supplementary Tables 1 and 2. Error bars are also given for the differential enthalpy of N_2 adsorption, but they are not distinguishable from the data points.

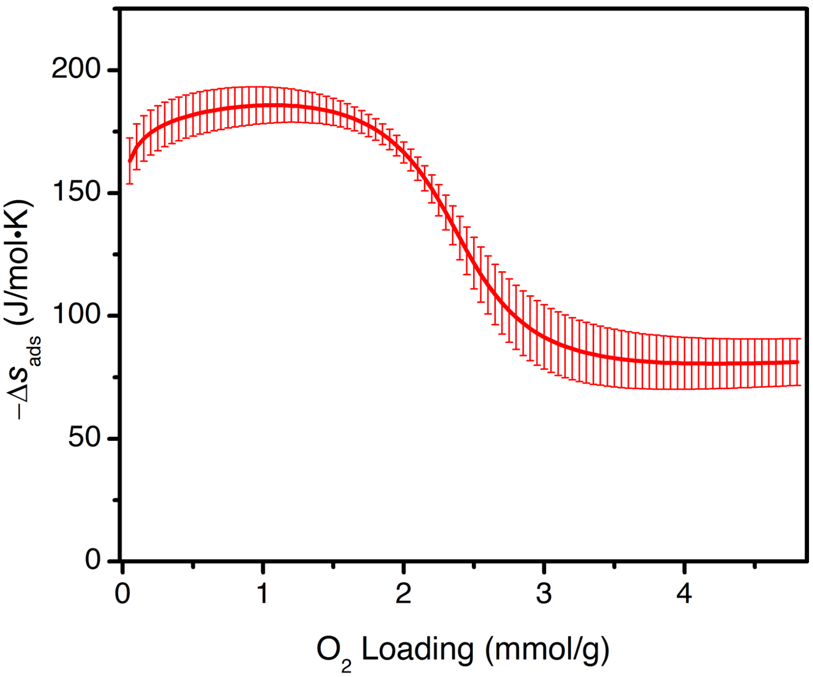

Supplementary Figure 15. Differential entropy of adsorption for O_2 adsorption in $Co_2(OH)_2$(bbta), determined using the Clausius–Clapeyron equation and the fit values shown in Supplementary Tables 1 and 2.

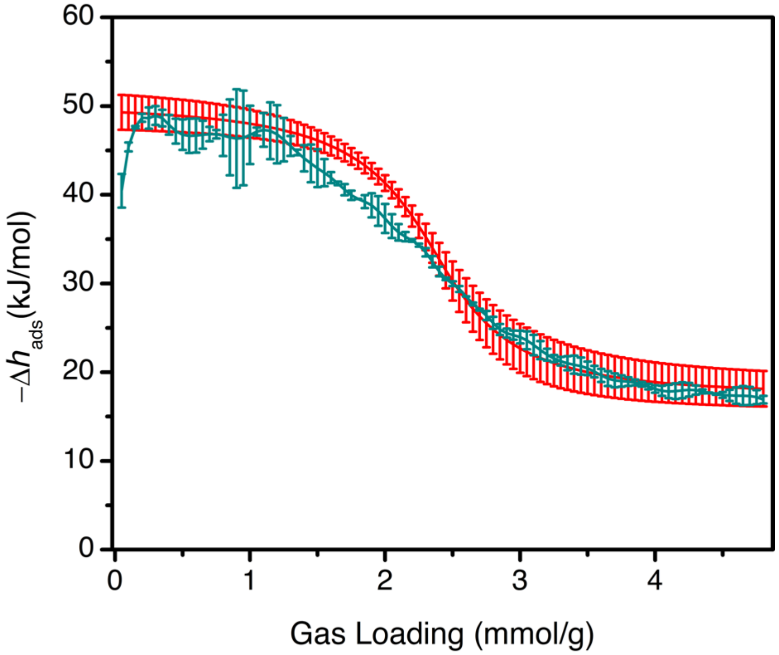

Supplementary Figure 16. A comparison of the differential enthalpy of adsorption for O_2 adsorption in $Co_2(OH)_2$(bbta), determined using the dual-site Langmuir expression and the Clausius–Clapeyron equation (red trace), and using spline interpolation and the Clausius–Clapeyron equation (teal trace). The close agreement within error at almost all loadings corroborates the use of the dual-site Langmuir equation in modeling O_2 adsorption in $Co_2(OH)_2$(bbta).

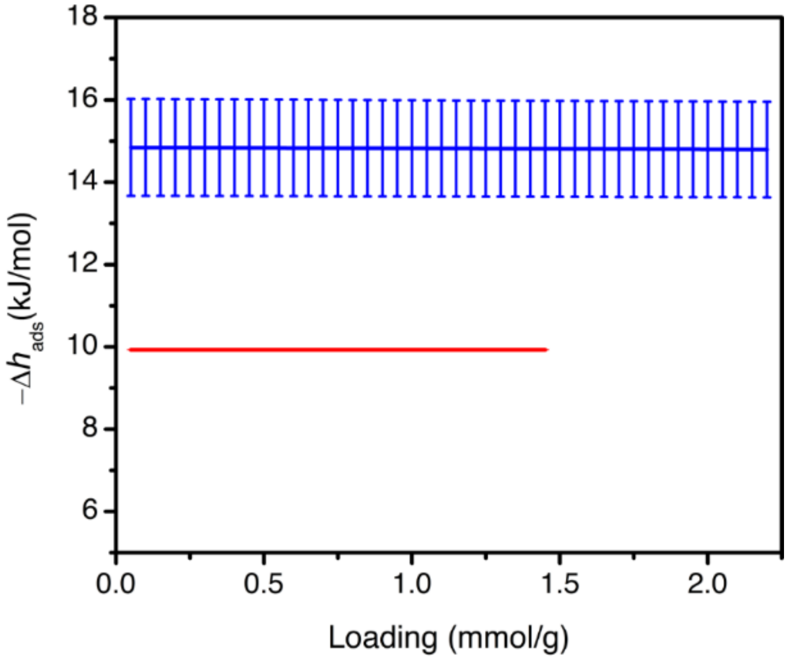

Supplementary Figure 17. Differential enthalpy of adsorption for O_2 and N_2 adsorption in Co_2Cl_2(bbta), determined using the Clausius–Clapeyron equation and the fits shown in Supplementary Tables 3 and 4. The differential enthalpy of adsorption of O_2 also has error bars displayed, though they are not distinguishable from the data points.

5. Ideal adsorbed solution theory calculations

Supplementary Table 5. Ideal adsorbed solution theory (IAST) selectivities for $Co_2(OH)_2$(bbta) at different temperatures, calculated using the Langmuir fits to the pure component adsorption isotherms for O_2 and N_2 (see Supplementary Tables 1 and 2), for a theoretical mixture of 0.21 bar O_2 and 0.79 bar N_2.

T (K)	IAST Selectivity (0.21 mbar O_2)	Purity of Adsorbed O_2 (%)	Amount O_2 Adsorbed (mmol/g)	Amount N_2 Adsorbed (mmol/g)
195	42.17	91.81	5.00	0.45
203	31.93	89.46	4.12	0.49
213	22.21	85.52	3.29	0.56
223	14.40	79.29	2.51	0.66

6. Synchrotron powder X-ray diffraction data and analysis

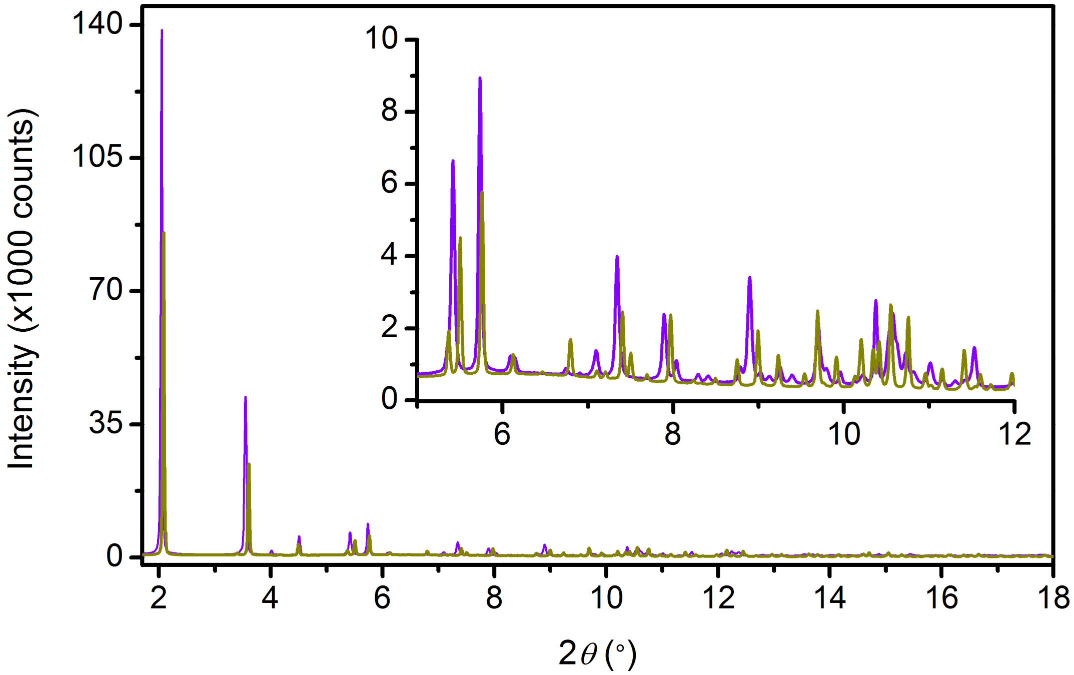

Supplementary Figure 18. Powder X-ray diffraction patterns of desolvated $Co_2(OH)_2$(bbta) (purple trace) and Co_2Cl_2(bbta) (green trace) collected under vacuum. The intensity differences between the two patterns are indicative of different structures. (λ = 0.45241 Å).

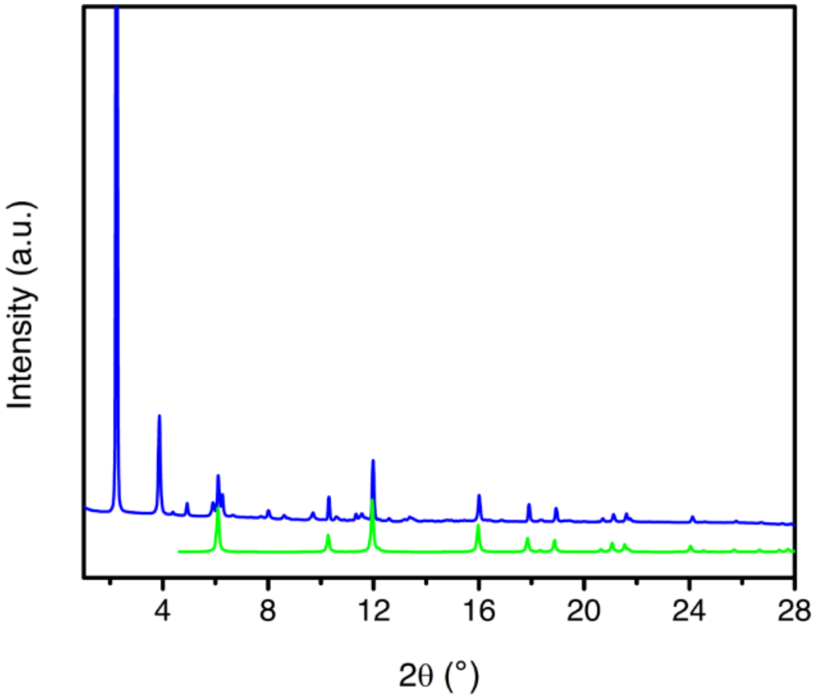

Supplementary Figure 19. Powder X-ray diffraction pattern of desolvated $Co_2(OH)_2$(bbta) synthesized using aqueous KOH (according to a literature procedure[2]) and the predicted powder X-ray diffraction pattern of $Co(OH)_2$ (green trace).[3] Peaks corresponding to $Co(OH)_2$ can be clearly distinguished in the diffraction pattern of the $Co_2(OH)_2$(bbta) sample. (λ = 0.4948 Å).

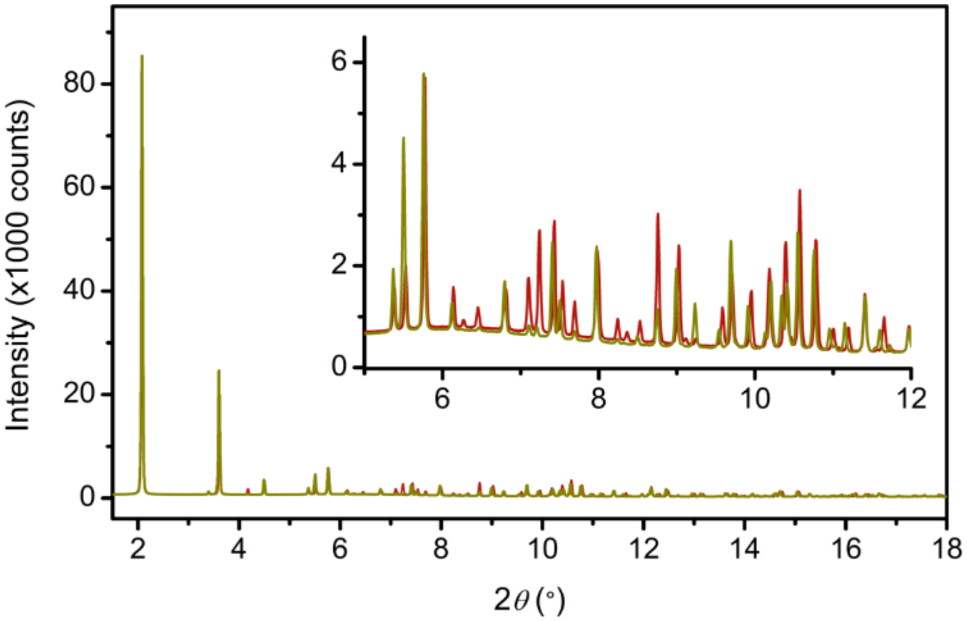

Supplementary Figure 20. Powder X-ray diffraction patterns of desolvated Co_2Cl_2(bbta) under vacuum at 298 K (green trace) and desolvated Co_2Cl_2(bbta) dosed with 27 mbar of O_2 at 195 K (dark red trace). The two patterns show only minor changes in the unit cells of the two samples, but there are clear intensity differences, indicative of O_2 adsorption within the pores of Co_2Cl_2(bbta) under the experimental conditions. (λ = 0.45241 Å).

Supplementary Table 6. Experimental, unit cell, and refinement parameters obtained by Rietveld refinement using synchrotron X-ray powder diffraction patterns of Co_2Cl_2(bbta) under vacuum and dosed with O_2 gas.

	Desolvated Co_2Cl_2(bbta)	27 mbar O_2
λ (Å)	0.45241	0.45241
Temperature	298 K	195 K
Space Group	$R\bar{3}m$	$R\bar{3}m$
a (Å)	24.9201(6)	24.8053(6)
c (Å)	8.15463(18)	8.17402(19)
Vol. (Å^3)	4385.7(2)	4355.7(2)
R_{wp} (%)	4.48	4.08
R_{exp} (%)	3.38	3.43
R_p (%)	3.34	2.85
R_{Bragg} (%)	2.98	2.39
GoF	1.32	1.19

Supplementary Table 7. Structural model obtained by Rietveld refinement of the synchrotron powder X-ray diffraction pattern of desolvated Co_2Cl_2(bbta) (T = 298 K). The refinement plot is shown in Supplementary Figure 21. Values in parenthesis indicate one standard deviation from the parameter value. Space group R–3m, a = 24.9201(6) Å, c = 8.15463(18) Å. Figures-of-merit (as defined by TOPAS): R_{wp} = 4.48%, R_p = 3.34%, R_{Bragg} = 2.98%, GoF = 1.32.

atom	*x*	*y*	*z*	*multiplicity*	*occupancy*	U_{iso} (Å^2)
Co	−0.04552(9)	1/3	1/3	18	1	0.0317(8)
Cl	−0.09035(16)	0.24298(16)	1/6	18	1	0.0512(16)
N1	0.0569(3)	0.31268(19)	0.2095(7)	36	1	0.0189(19)[a]
N2	0.0373(5)	1/3	1/3	18	1	0.0189(19)[a]
C1	0.1122(3)	0.3187(2)	0.2564(5)	36	1	0.0189(19)[a]
C2	0.1525(5)	0.3050(5)	0.1747(9)	18	1	0.0189(19)[a]
H1	0.1911	0.3822	0.6140	18	1	0.0189(19)[a]

[a]The thermal parameters for all of atoms of the bbta^{2-} ligand were constrained to be equivalent.

Supplementary Table 8. Structural model obtained by Rietveld refinement of the synchrotron powder X-ray diffraction pattern of desolvated Co_2Cl_2(bbta) dosed with 27 mbar of O_2 (T = 195 K). The refinement plot is shown in Supplementary Figure 22. Values in parenthesis indicate one standard deviation from the parameter value. Space group R–3m, a = 24.8053(6) Å, c = 8.17402(19) Å. Figures-of-merit (as defined by TOPAS): R_{wp} = 4.08%, R_p = 2.85%, R_{Bragg} = 2.39%, GoF = 1.19.

atom	*x*	*y*	*z*	*multiplicity*	*occupancy*	U_{iso} (Å^2)
Co	−0.04636(8)	1/3	1/3	18	1	0.0469(13)
Cl	−0.08901(13)	0.24432(13)	1/6	18	1	0.048(5)
N1	0.0569(3)	0.3127(3)	0.2095(7)	36	1	0.0063(16)[a]
N2	0.0373(4)	1/3	1/3	18	1	0.0063(16)[a]
C1	0.1122(3)	0.3187(3)	0.2564(4)	36	1	0.0063(16)[a]
C2	0.1525(6)	0.3050(5)	0.1747(10)	18	1	0.0063(16)[a]
H1	0.1422	0.2845	0.0528	18	1	0.0063(16)[a]
O1	0	0.1812(6)	0	18	0.880(10)	0.068(6)
O2	0.0120(16)	0.1578(13)	−0.099(3)	36	0.929(8)	0.199(8)
O3	−0.0206(5)	0.1722(4)	0.4592(16)	36	0.440(5)	0.253(19)

[a]The thermal parameters for all of atoms of the bbta^{2-} ligand were constrained to be equivalent.

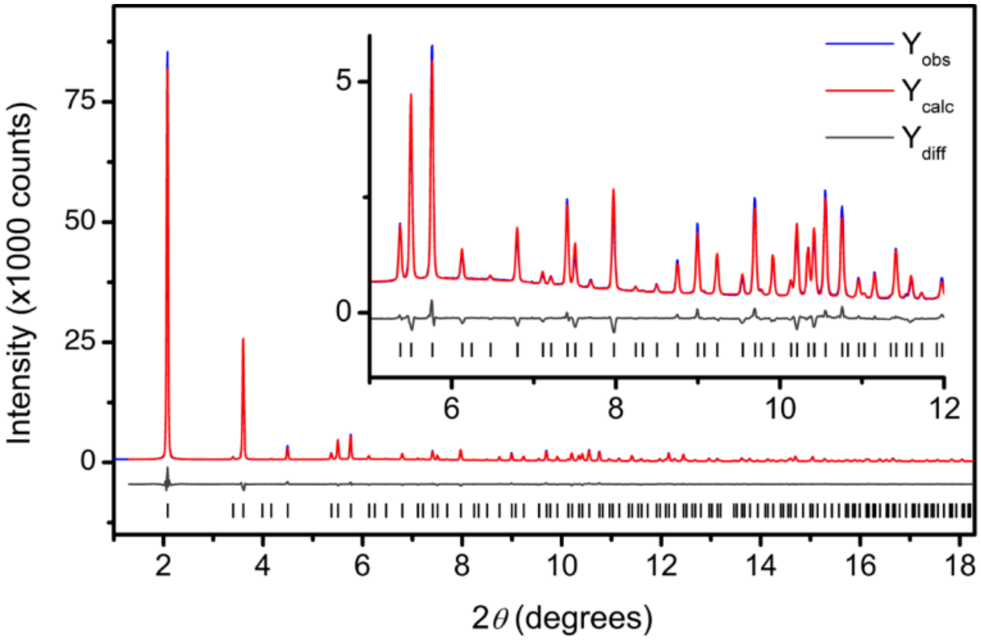

Supplementary Figure 21. Rietveld refinement with synchrotron powder X-ray diffraction data for desolvated Co_2Cl_2(bbta) collected under vacuum at 298 K from 1.5º to 18.3º. Blue and red lines represent the observed and calculated diffraction patterns, respectively. The gray line represents the difference pattern and the black tick marks indicate calculated Bragg peak positions. The inset shows a magnified view of the high angle region. Figures-of-merit (as defined by TOPAS): R_{wp} = 4.48%, R_p = 3.34%, R_{exp} = 3.38%, R_{Bragg} = 2.98%, GoF = 1.32. (λ = 0.45241 Å).

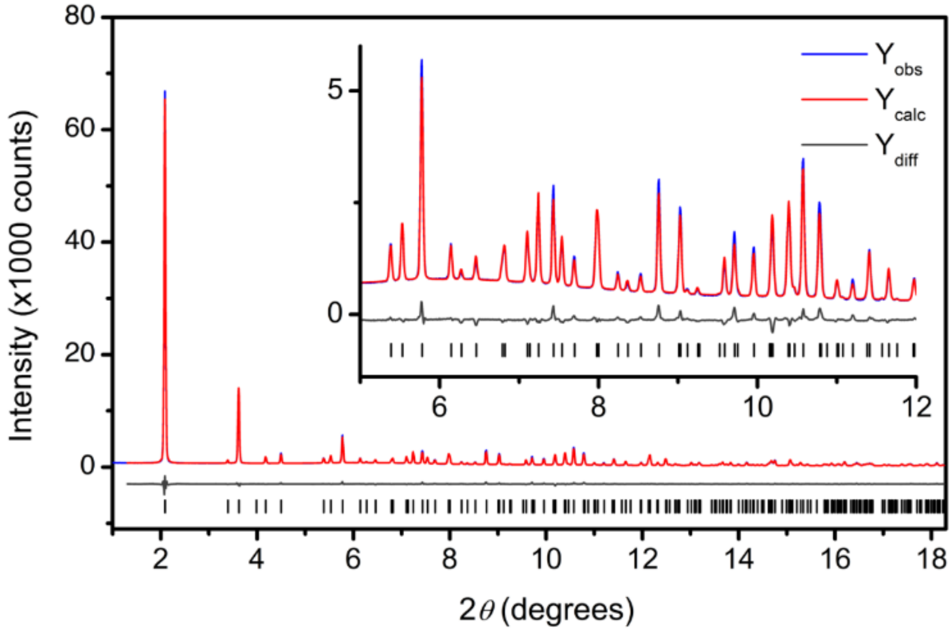

Supplementary Figure 22. Rietveld refinement with synchrotron powder X-ray diffraction data for desolvated Co_2Cl_2(bbta) dosed with 27 mbar of O_2 collected at 195 K from 1.5º to 18.3º. Blue and red lines represent the observed and calculated diffraction patterns, respectively. The gray line represents the difference between observed and calculated patterns, and the black tick marks indicate calculated Bragg peak positions. The inset shows a magnified view of the high angle

region. Figures-of-merit (as defined by TOPAS): R_{wp} = 4.08%, R_p = 2.85%, R_{exp} = 3.43%, R_{Bragg} = 2.39%, GoF = 1.19. (λ = 0.45241 Å).

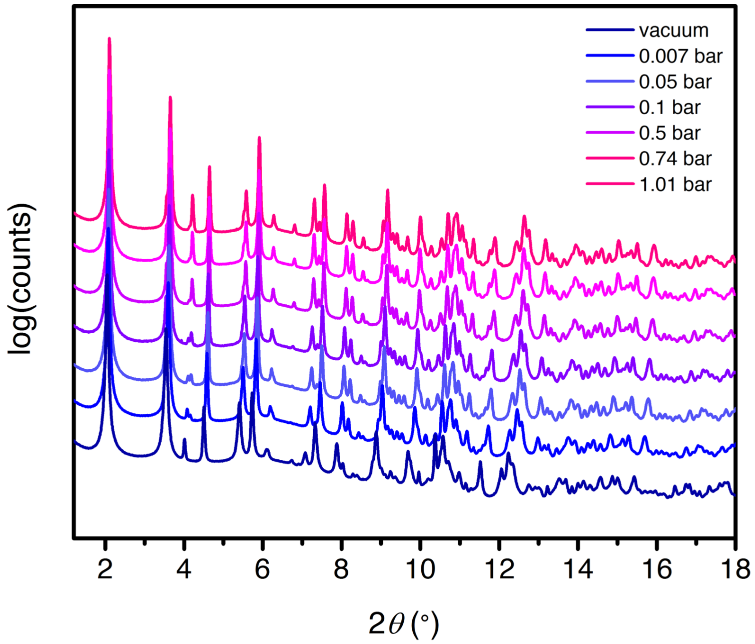

Supplementary Figure 23. Powder X-ray diffraction patterns of $Co_2(OH)_2$(bbta) dosed with increasing pressures of O_2 at 195 K, from vacuum (navy trace) to 1.01 bar of O_2 (red trace). As the pressure of O_2 increases, the allowed reflections shift to higher angles, indicative of shrinkage of the framework unit cell resulting from oxidation of the cobalt(II) centers of the material as cobalt(III)-superoxo species are generated. (λ = 0.45236 Å).

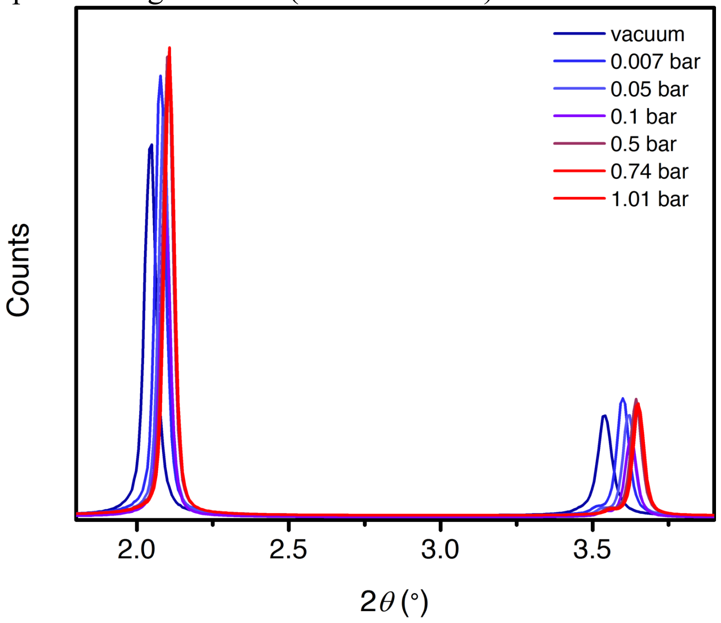

Supplementary Figure 24. Powder X-ray diffraction patterns of $Co_2(OH)_2$(bbta) dosed with increasing pressures of O_2 at 195 K, from vacuum (navy trace) to 1.01 bar of O_2 (red trace). As the pressure of O_2 increased, the allowed reflections shifted to higher angles, indicative of an increased shrinkage of the framework unit cell resulting from oxidation of the cobalt(II) centers to generate cobalt(III)-superoxo species. (λ = 0.45236 Å).

Supplementary Table 9. Experimental, unit cell, and refinement parameters obtained from Rietveld refinement using synchrotron X-ray powder diffraction patterns of $Co_2(OH)_2$(bbta) under vacuum and dosed with O_2.

	Desolvated $Co_2(OH)_2$(bbta)	6.7 mbar O_2	46.7 mbar O_2
λ (Å)	0.45236	0.45236	0.45236
Temperature	195 K	195 K	195 K
Space Group	$R\bar{3}m$	$R\bar{3}m$	$R\bar{3}m$
a (Å)	25.3186(8)	24.9432(19)	24.8075(9)
c (Å)	7.9757(4)	7.8488(5)	7.8055(4)
Vol. (Å^3)	4427.7(3)	4229.0(7)	4160.0(4)
R_{wp} (%)	4.23	4.00	4.00
R_{exp} (%)	1.93	2.11	2.29
R_p (%)	3.17	3.10	3.05
R_{Bragg} (%)	1.84	1.83	2.00
GoF	1.51	1.89	1.75
O_2 occ. on open metal site (%)	–	42.4(12)	55.3(12)

Supplementary Table 10. Experimental, unit cell, and refinement parameters obtained from Rietveld refinement using synchrotron X-ray powder diffraction patterns of $Co_2(OH)_2$(bbta) under vacuum and dosed with O_2.

	100 mbar O_2	503 mbar O_2	741 mbar O_2	1014 mbar O_2
λ (Å)	0.45236	0.45236	0.45236	0.45236
Temperature	195 K	195 K	195 K	195 K
Space Group	$R\bar{3}m$	$R\bar{3}m$	$R\bar{3}m$	$R\bar{3}m$
a (Å)	24.7567(16)	24.6356(14)	24.6008(13)	24.5748(14)
c (Å)	7.7898(4)	7.7546(4)	7.7451(4)	7.7385(4)
Vol. (Å^3)	4134.7(6)	4075.9(5)	4059.3(5)	4047.4(5)
R_{wp} (%)	3.90	3.53	3.49	3.53
R_{exp} (%)	2.59	2.10	2.10	2.10
R_p (%)	2.93	2.73	2.65	2.67
R_{Bragg} (%)	1.85	1.15	1.04	0.91
GoF	1.50	1.68	1.66	1.68
O_2 occ. on open metal site (%)	59.8(11)	68.0(11)	71.4(11)	75.1(10)
O_2 occ. in pore site (%)	8.3(10)	31.0(9)	38.9(9)	46.0(9)

Supplementary Table 11. Structural model obtained by Rietveld refinement using synchrotron powder X-ray diffraction data of desolvated $Co_2(OH)_2$(bbta) (T = 195 K). The refinement plot is shown in Supplementary Figure 26. Values in parenthesis indicate one standard deviation from the parameter value. Space group $R\text{–}3m$, a = 25.3186(8) Å, c = 7.9757(4) Å. Figures-of-merit (as defined by TOPAS): R_{wp} = 4.23%, R_p = 3.17%, R_{Bragg} = 1.84%, GoF = 1.51.

atom	*x*	*y*	*z*	*multiplicity*	*occupancy*	U_{iso} (Å^2)
Co	0.95788(10)	1/3	1/3	18	1	0.0469(13)
O	1	0.4078(4)	0.5	18	1	0.048(5)
N1	0.6425(4)	0.7269(2)	0.4598(12)	36	1	0.047(3)[a]
N2	2/3	0.7039(5)	1/3	18	1	0.047(3)[a]
C1	0.6549(7)	0.7806(5)	0.4154(7)	36	1	0.047(3)[a]
C2	0.6438(2)	0.8219(2)	0.4985(17)	18	1	0.047(3)[a]
H1	0.6267	0.8133	0.6267	18	1	0.047(3)[a]

[a]The thermal parameters for all of atoms of the bbta^{2-} ligand were constrained to be equivalent.

Supplementary Table 12. Structural model obtained by Rietveld refinement using a synchrotron powder X-ray diffraction pattern of desolvated $Co_2(OH)_2$(bbta) dosed with 1.014 bar of O_2 (T = 195 K). The refinement plot is shown in Supplementary Figure 32.. Values in parenthesis indicate one standard deviation from the parameter value. Space group R–3m, a = 24.5748(14) Å, c = 7.7385(4) Å. Figures-of-merit (as defined by TOPAS): R_{wp} = 3.53%, R_p = 2.67%, R_{Bragg} = 0.91%, GoF = 1.68.

atom	*x*	*y*	*z*	*multiplicity*	*occupancy*	U_{iso} (Å^2)
Co	0.95642(10)	1/3	1/3	18	1	0.0469(13)
O1	1	0.4016(4)	0.5	18	1	0.040(4)
O2	0.2132(3)	0.2132(3)	0	18	0.751(10)	0.075(8)
O3	0.2045(4)	0.1688(6)	0.1085(15)	36	0.375(10)	0.083(13)
O4	0.2881(9)	0.4640(8)	0.108(4)	36	0.460(9)	0.253(19)
N1	0.6473(4)	0.7211(4)	0.4671(12)	36	1	0.028(2)[a]
N2	2/3	0.7027(6)	1/3	18	1	0.028(2)[a]
C1	0.6540(4)	0.7781(4)	0.4182(10)	36	1	0.028(2)[a]
C2	0.6406(7)	0.8203(4)	0.5030(19)	18	1	0.028(2)[a]
H1	0.6203	0.8101	0.6321	18	1	0.028(2)[a]

[a]The thermal parameters for all of atoms of the bbta^{2-} ligand were constrained to be equivalent.

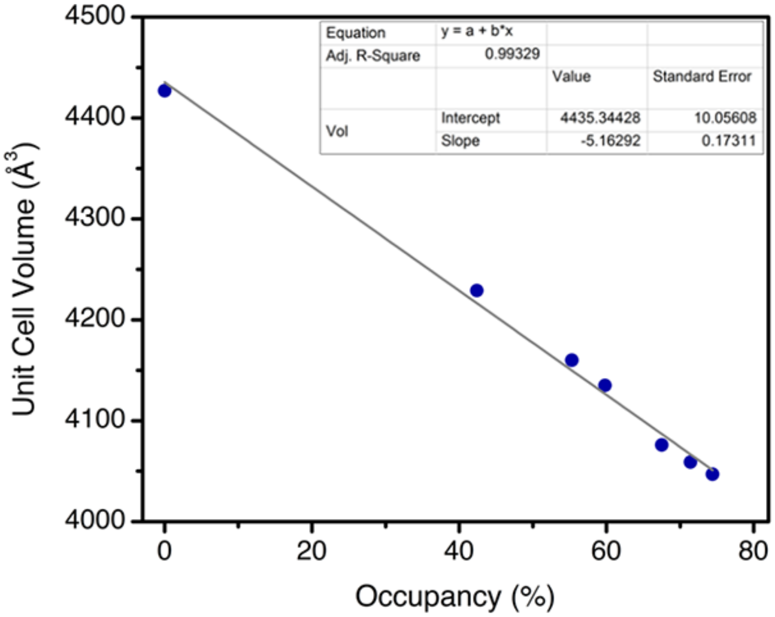

Supplementary Figure 25. A plot of the refined superoxo occupancies vs. sample unit cell volumes (symbols), as obtained by Rietveld refinement using data collected on samples of $Co_2(OH)_2$(bbta) dosed with increasing pressures of O_2 gas at 195 K. The gray line is a linear fit to the data according to $y = 4435 \times x - 5.16$, with errors as described in the inset box on the graph. The graph shows that the observed unit cell volume may correlate with the occupancy of the superoxo in the ranges measured here.

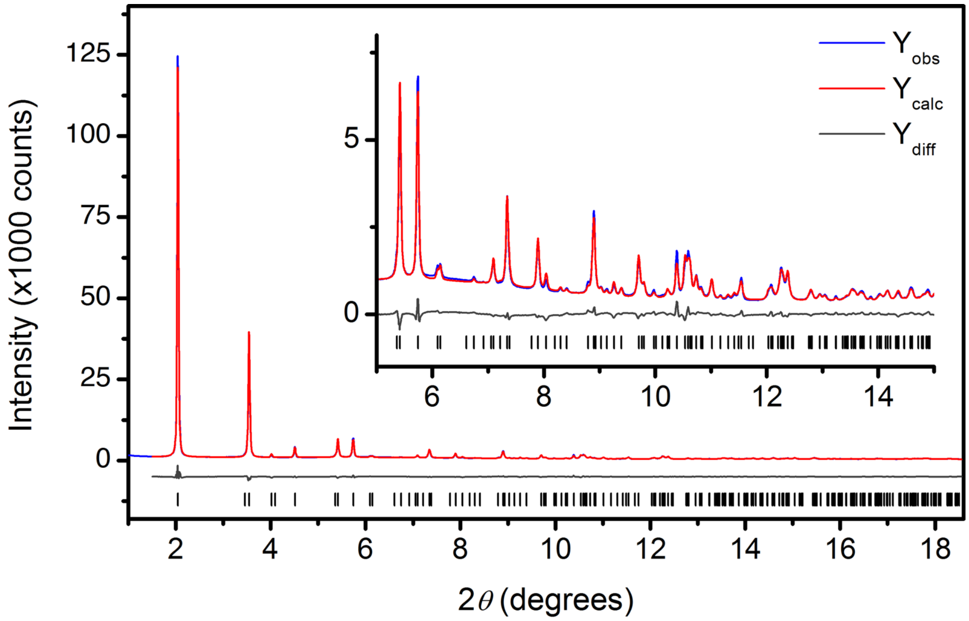

Supplementary Figure 26. Rietveld refinement with synchrotron powder X-ray diffraction data for desolvated $Co_2(OH)_2$(bbta) (blue line) under vacuum at 195 K from 1.5º to 18.6º. The red line represents the calculated diffraction pattern, the gray line represents the difference between observed and calculated patterns, and the black tick marks indicate calculated Bragg peak positions. The inset shows the high angle region at a magnified scale. Figures-of-merit (defined by TOPAS): R_{wp} = 4.23%, R_p = 3.17%, R_{exp} = 1.93%, R_{Bragg} = 1.84%, GoF = 1.51. (λ = 0.45236 Å).

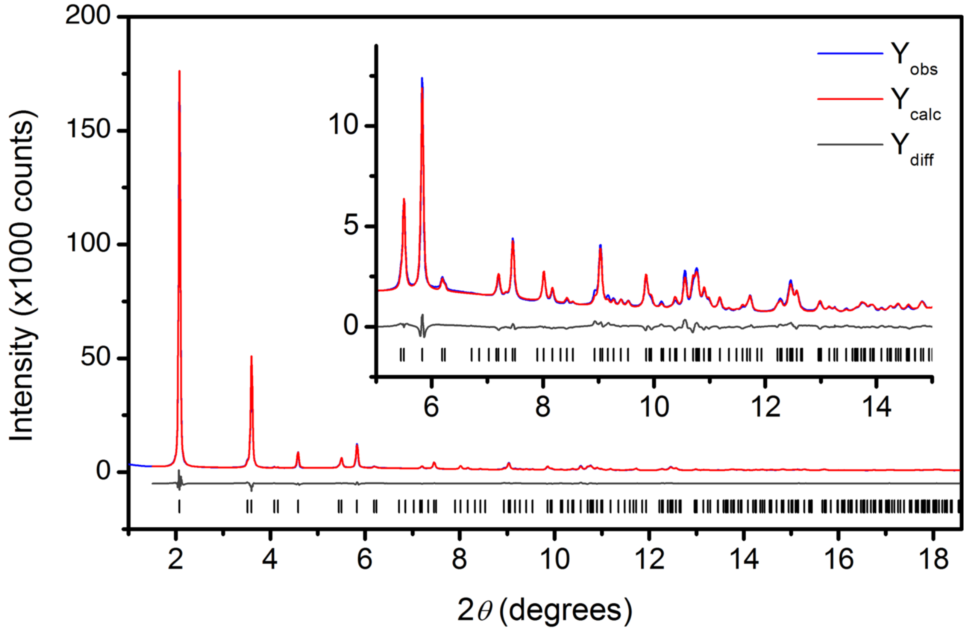

Supplementary Figure 27. Rietveld refinement with powder X-ray diffraction data for $Co_2(OH)_2$(bbta) (blue line) dosed with 6.7 mbar of O_2 at 195 K from 1.5º to 18.6º. The red line represents the calculated diffraction pattern, the gray line represents the difference between observed and calculated patterns, and the black tick marks indicate calculated Bragg peak positions. The inset shows the high angle region at a magnified scale. Figures-of-merit (defined by TOPAS): R_{wp} = 4.00%, R_p = 3.10%, R_{exp} = 2.11%, R_{Bragg} = 1.83%, GoF = 1.89. (λ = 0.45236 Å).

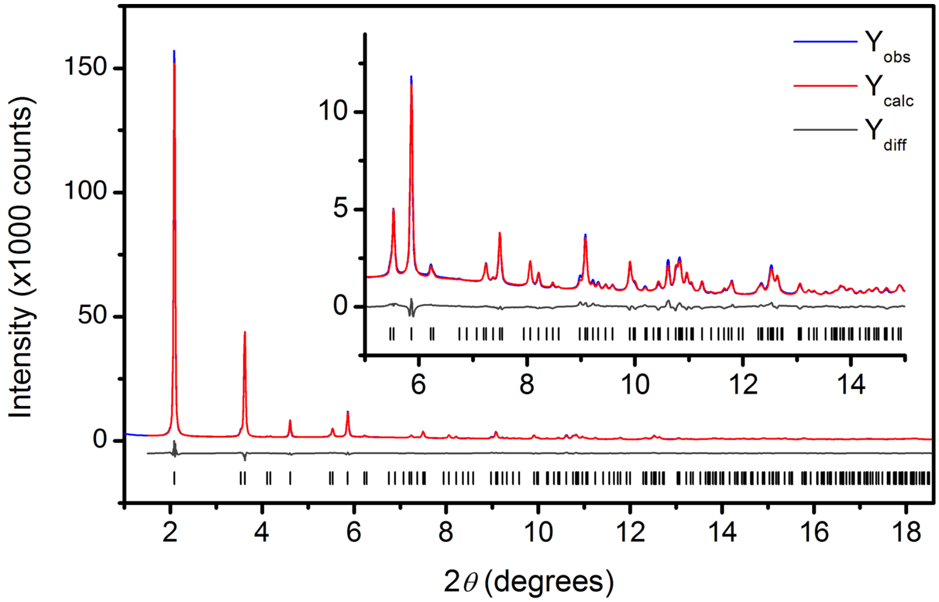

Supplementary Figure 28. Rietveld refinement with powder X-ray diffraction data for $Co_2(OH)_2$(bbta) (blue line) dosed with 46.7 mbar of O_2 at 195 K from 1.5º to 18.6º. The red line represents the calculated diffraction pattern, the gray line represents the difference between observed and calculated patterns, and the black tick marks indicate calculated Bragg peak positions. The inset shows the high angle region at a magnified scale. Figures-of-merit (as defined by TOPAS): R_{wp} = 4.00%, R_p = 3.05%, R_{exp} = 2.29%, R_{Bragg} = 2.00%, GoF = 1.75. (λ = 0.45236 Å).

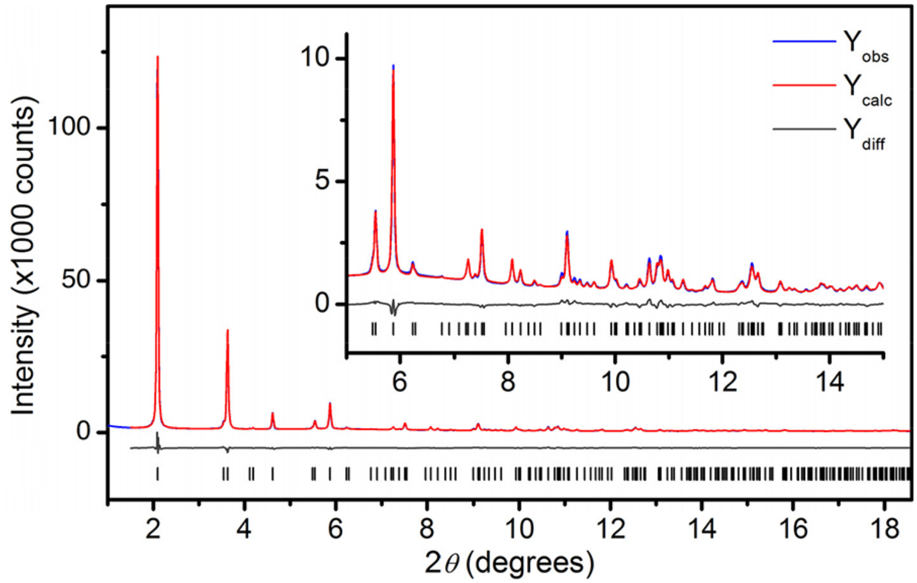

Supplementary Figure 29. Rietveld refinement with powder X-ray diffraction data for $Co_2(OH)_2$(bbta) dosed with 100 mbar of O_2 at 195 K from 1.5º to 18.6º (blue line). The red line represents the calculated diffraction pattern, the gray line represents the difference between observed and calculated patterns, and the black tick marks indicate calculated Bragg peak positions. The inset shows the high angle region at a magnified scale. Figures-of-merit (as defined by TOPAS): R_{wp} = 3.90%, R_p = 2.93%, R_{exp} = 2.59%, R_{Bragg} = 1.85%, GoF = 1.50. (λ = 0.45236 Å).

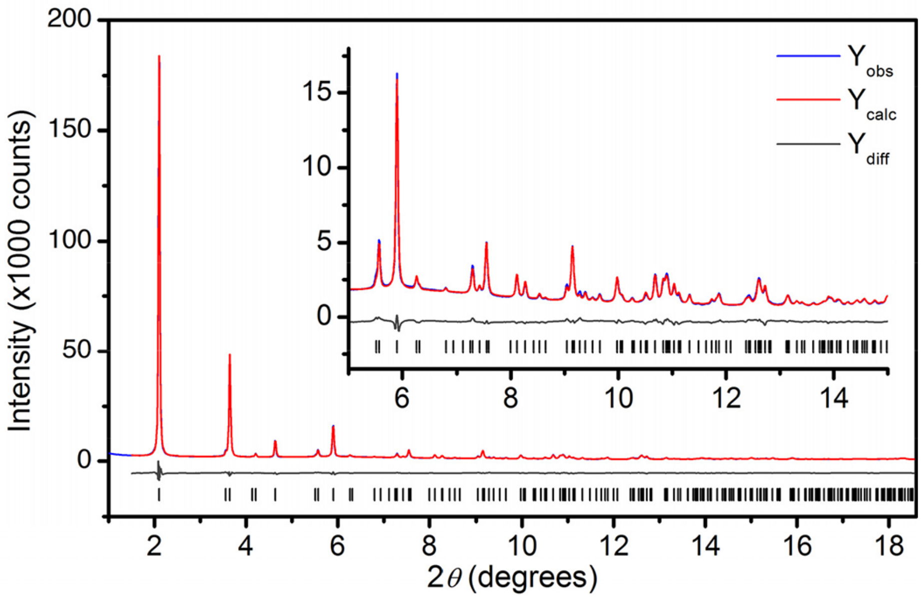

Supplementary Figure 30. Rietveld refinement with powder X-ray diffraction data for $Co_2(OH)_2$(bbta) dosed with 503 mbar of O_2 at 195 K from 1.5º to 18.6º (blue line). The red line represents the calculated diffraction pattern, the gray line represents the difference between observed and calculated patterns, and the black tick marks indicate calculated Bragg peak positions. The inset shows the high angle region at a magnified scale. Figures-of-merit (defined by TOPAS): R_{wp} = 3.53%, R_p = 2.73%, R_{exp} = 2.10%, R_{Bragg} = 1.15%, GoF = 1.68. (λ = 0.45236 Å).

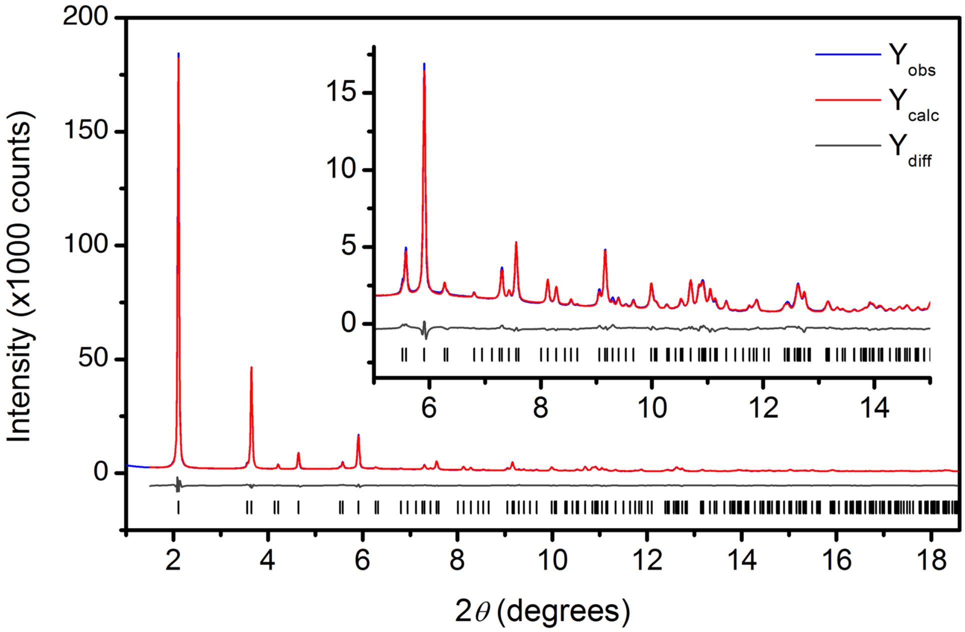

Supplementary Figure 31. Rietveld refinement with powder X-ray diffraction data for $Co_2(OH)_2$(bbta) dosed with 741 mbar of O_2 at 195 K from 1.5º to 18.6º (blue line). The red line represents the calculated diffraction pattern, the gray line represents the difference between observed and calculated patterns, and the black tick marks indicate calculated Bragg peak positions. The inset shows the high angle region at a magnified scale. Figures-of-merit (defined by TOPAS): R_{wp} = 3.49%, R_p = 2.65%, R_{exp} = 2.10%, R_{Bragg} = 1.04%, GoF = 1.66. (λ = 0.45236 Å).

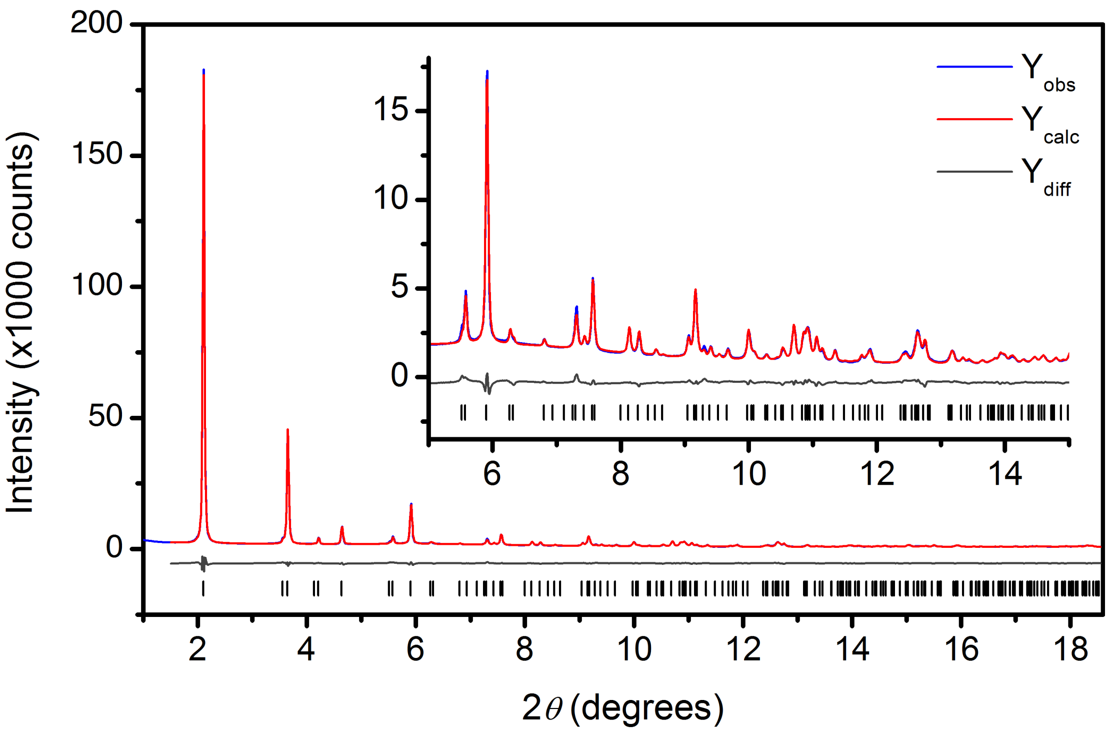

Supplementary Figure 32. Rietveld refinement with powder X-ray diffraction data for $Co_2(OH)_2$(bbta) dosed with 1.014 bar of O_2 at 195 K from 1.5° to 18.6° (blue line). The red line represents the calculated diffraction pattern, the gray line represents the difference between observed and calculated patterns, and the black tick marks indicate calculated Bragg peak positions. The inset shows the high angle region at a magnified scale. Figures-of-merit (defined by TOPAS): R_{wp} = 3.53%, R_p = 2.67%, R_{exp} = 2.10%, R_{Bragg} = 0.91%, GoF = 1.68. (λ = 0.45236 Å).

7. Powder neutron diffraction data collection and analysis

Supplementary Table 13. Experimental, unit cell, and refinement parameters obtained by Rietveld refinement using neutron powder diffraction patterns of $Co_2(OH)_2$(bbta) under vacuum and under 1 eq. of O_2 gas.

	Desolvated $Co_2(OH)_2$(bbta)	O_2-dosed $Co_2(OH)_2$(bbta)	O_2-dosed $Co_2(OH)_2$(bbta)
λ (Å)	2.0772	2.0772	2.0772
Temperature	6 K	150 K	8 K
Space Group	$R\bar{3}m$	$R\bar{3}m$	$R\bar{3}m$
a (Å)	25.3775(9)	24.4658(8)	24.4276(7)
c (Å)	7.9742(4)	7.6786(4)	7.6633(4)
Vol. (Å^3)	4447.5(4)	3980.4(3)	3960.1(3)
R_{wp} (%)	3.07	3.05	2.93
R_{exp} (%)	1.94	2.78	2.81
R_p (%)	2.31	2.57	2.50
R_{Bragg} (%)	8.76	4.89	4.48
GoF	1.60	1.13	1.07
O_2 occ. (%)	–	86.9(8)	87.2(7)

Supplementary Table 14. Structural model obtained by Rietveld refinement using a neutron powder diffraction pattern of desolvated $Co_2(OH)_2$(bbta) obtained at 6 K. The refinement plot is shown in Supplementary Figure 34. Values in parenthesis indicate one standard deviation from the parameter value. Temperature = 6 K, space group *R*–3*m*, *a* = 25.3775(9) Å, *c* = 7.9742(4) Å. Figures-of-merit (as defined by GSAS): R_{wp} = 3.07%, R_p = 2.31%, R_{Bragg} = 8.76%, GoF = 1.60.

atom	*x*	*y*	*z*	*multiplicity*	*occupancy*	U_{iso} (Å^2)
Co	0.9592(10)	1/3	1/3	18	1	0.010(5)
O1	1	0.5975(8)	0.5	18	1	0.028(3)
H1	0.3033(12)	0.2287(7)	0.248(2)	36	0.5	0.047(7)
N1	0.6453(15)	0.7250(3)	0.4599(4)	36	1	0.0196(3)[a]
N2	2/3	0.70722(19)	1/3	18	1	0.0196(3)[a]
C1	0.65483(18)	0.7830(4)	0.4182(4)	36	1	0.0196(3)[a]
C2	0.6413(4)	0.8206(5)	0.5059(7)	18	1	0.0196(3)[a]
H2	0.7124(6)	0.8562(10)	0.0377(13)	18	1	0.0196(3)[a]

[a]The thermal parameters for all of atoms of the $bbta^{2-}$ ligand were constrained to be equivalent.

Supplementary Table 15. Structural model obtained by Rietveld refinement using a neutron powder diffraction pattern of desolvated $Co_2(OH)_2$(bbta) dosed with 1 equivalent of O_2 and obtained at 150 K. The refinement plot is shown in Supplementary Figure 36. Values in parenthesis indicate one standard deviation from the parameter value. Temperature = 150 K, space group *R*–3*m*, a = 24.4658(8) Å, c = 7.6786(4) Å. Figures-of-merit (as defined by GSAS): R_{wp} = 3.05%, R_p = 2.57%, R_{Bragg} = 4.89%, GoF = 1.13.

atom	*x*	*y*	*z*	*multiplicity*	*occupancy*	U_{iso} (Å^2)
Co	0.9569(5)	1/3	1/3	18	1	0.017(4)
O1	1	0.5960(3)	0.5	18	1	0.0142(14)
H1	0.3077(8)	0.2292(6)	0.2489(15)	36	0.5	0.013(5)
O2	0.2156(4)	0.2156(4)	0	18	0.869(8)	0.0159(19)
O3	0.1934(7)	0.1722(5)	0.1061(14)	36	0.434(4)	0.037(4)
O4	0.3022(13)	0.4500(12)	0.210(4)	36	0.203(8)	0.032(9)
N1	0.64953(15)	0.72443(12)	0.4698(4)	36	1	0.01279(19)[a]
N2	2/3	0.70363(17)	1/3	18	1	0.01279(19)[a]
C1	0.65467(18)	0.78042(16)	0.4215(4)	36	1	0.01279(19)[a]
C2	0.6432(3)	0.82161(15)	0.5135(7)	18	1	0.01279(19)[a]
H2	0.7089(5)	0.8545(3)	0.0269(11)	18	1	0.01279(19)[a]

[a]The thermal parameters for all of atoms of the $bbta^{2-}$ ligand were constrained to be equivalent.

Supplementary Table 16. Structural model obtained by Rietveld refinement using a neutron powder diffraction pattern of desolvated $Co_2(OH)_2(bbta)$ dosed with 1 equivalent of O_2 and obtained at 8 K. The refinement plot is shown in Supplementary Figure 37. Values in parenthesis indicate one standard deviation from the parameter value. Temperature = 8 K, space group *R–3m*, a = 24.4276(7) Å, c = 7.6633(4) Å. Figures-of-merit (as defined by GSAS): R_{wp} = 2.93%, R_p = 2.50%, R_{Bragg} = 4.48%, GoF = 1.07.

atom	*x*	*y*	*z*	*multiplicity*	*occupancy*	U_{iso} (Å^2)
Co	0.9569(5)	1/3	1/3	18	1	0.016(4)
O1	1	0.5959(3)	0.5	18	1	0.0130(13)
H1	0.3061(7)	0.2284(6)	0.2431(14)	36	0.5	0.019(5)
O2	0.2132(3)	0.2132(3)	0	18	0.872(7)	0.0101(16)
O3	0.2045(4)	0.1688(6)	0.1085(15)	36	0.436(4)	0.032(4)
O4	0.2881(9)	0.4640(8)	0.108(4)	36	0.185(7)	0.038(9)
N1	0.64902(13)	0.72410(11)	0.4680(3)	36	1	0.01272(17)[a]
N2	2/3	0.70241(15)	1/3	18	1	0.01272(17)[a]
C1	0.65494(15)	0.78126(14)	0.4208(4)	36	1	0.01272(17)[a]
C2	0.6420(3)	0.82101(13)	0.5193(6)	18	1	0.01272(17)[a]
H2	0.7097(5)	0.8549(3)	0.0241(10)	18	1	0.01272(17)[a]

[a]The thermal parameters for all of atoms of the $bbta^{2-}$ ligand were constrained to be equivalent.

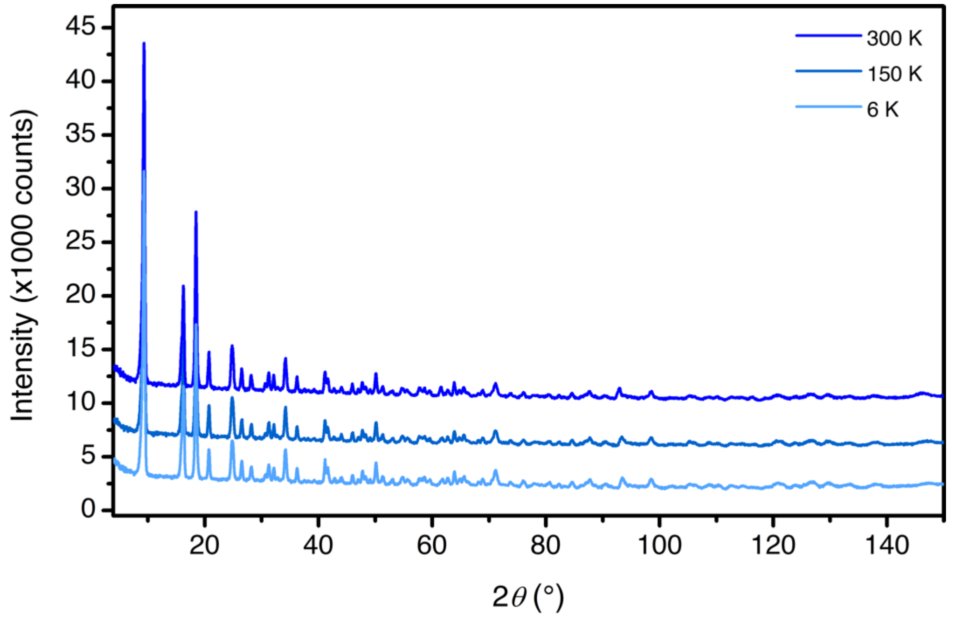

Supplementary Figure 33. Powder neutron diffraction data of desolvated $Co_2(OH)_2(bbta)$ obtained at 300, 150, and 6 K (navy, blue, and pale blue traces respectively) with a wavelength of 2.0774 Å from 4° to 150°. The patterns show very little difference between them in peak positions and intensities, indicating that the material does not change to any significant degree in structure upon cooling to 6 K from room temperature.

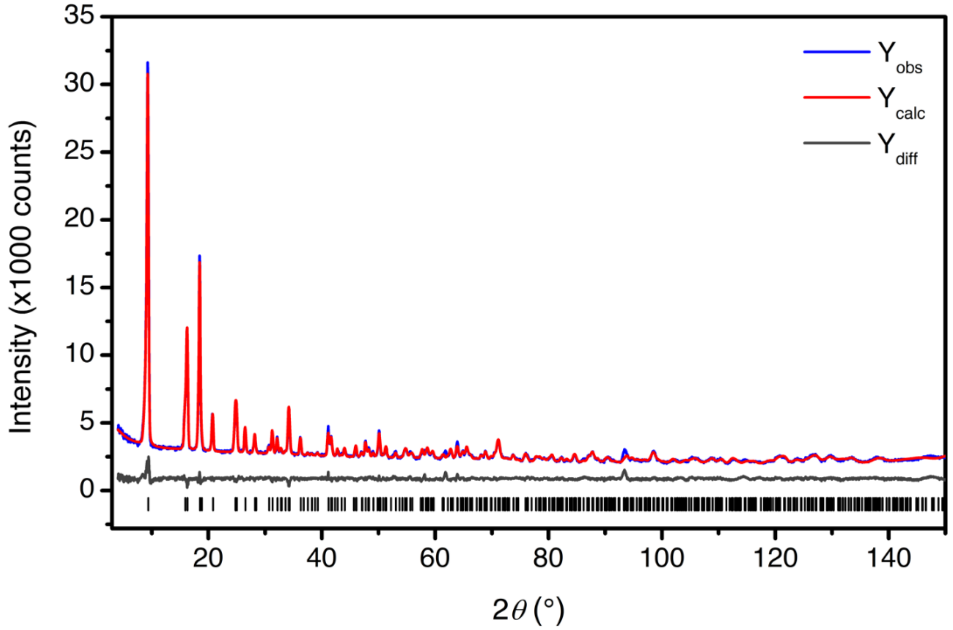

Supplementary Figure 34. Rietveld refinement with powder neutron diffraction data of desolvated $Co_2(OH)_2$(bbta) obtained at 6 K with a wavelength of 2.0774 Å from 5º to 150º (blue line). The red line represents the calculated diffraction pattern, the gray line represents the difference between observed and calculated patterns, and the black tick marks indicate calculated Bragg peak positions. Figures-of-merit (as defined by TOPAS): R_{wp} = 3.07%, R_p = 2.31%, R_{exp} = 1.93%, R_{Bragg} = 8.76%, GoF = 1.60.

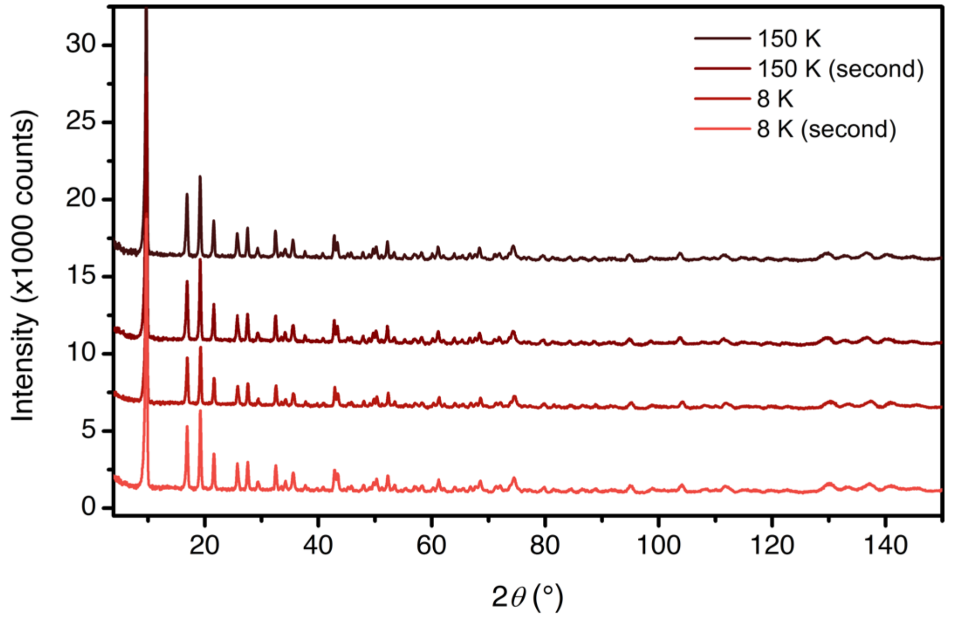

Supplementary Figure 35. Powder neutron diffraction data of desolvated $Co_2(OH)_2$(bbta) dosed with 1 equivalent of O_2, obtained at 8 and 150 K as described in the methods (pale red to brown traces) with a wavelength of 2.0772 Å from 4º to 150 º. The patterns show very little difference between them in peak positions and intensities, indicating that the material does not change to any significant degree in structure upon cycling the temperature from 8 K up to room temperature and back down to 8 K, and particularly between patterns taken at the same temperature.

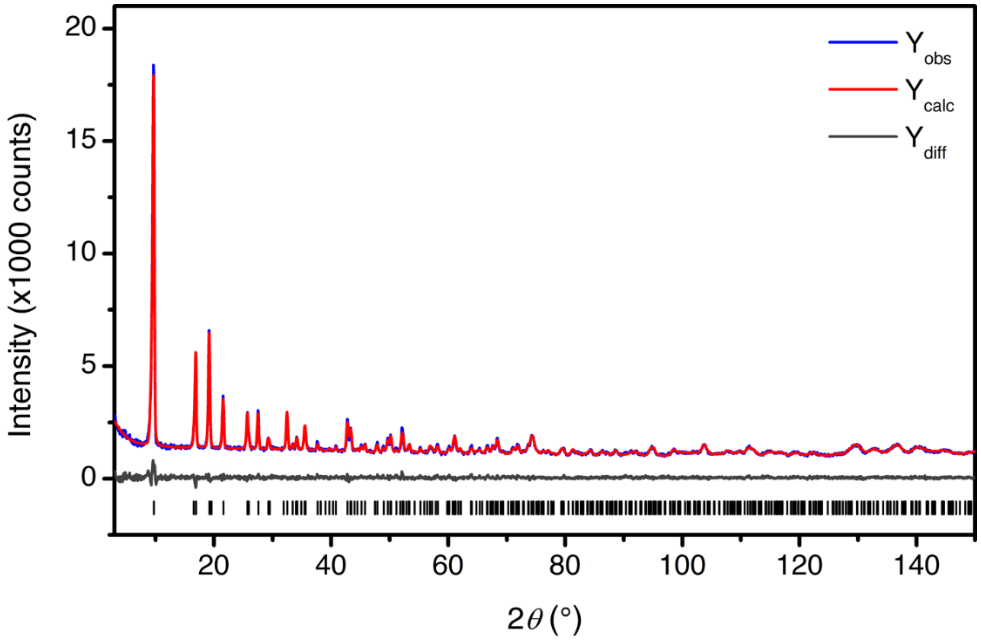

Supplementary Figure 36. Rietveld refinement with powder neutron diffraction data of desolvated $Co_2(OH)_2$(bbta) dosed with 1 equivalent of O_2, obtained at 150 K with a wavelength of 2.0772 Å, from 5º to 150º (blue line). The red line represents the calculated diffraction pattern, the gray line represents the difference between observed and calculated patterns, and the black tick marks indicate calculated Bragg peak positions. Figures-of-merit (as defined by GSAS): R_{wp} = 3.05%, R_p = 2.57%, R_{exp} = 2.78%, R_{Bragg} = 4.89%, GoF = 1.13.

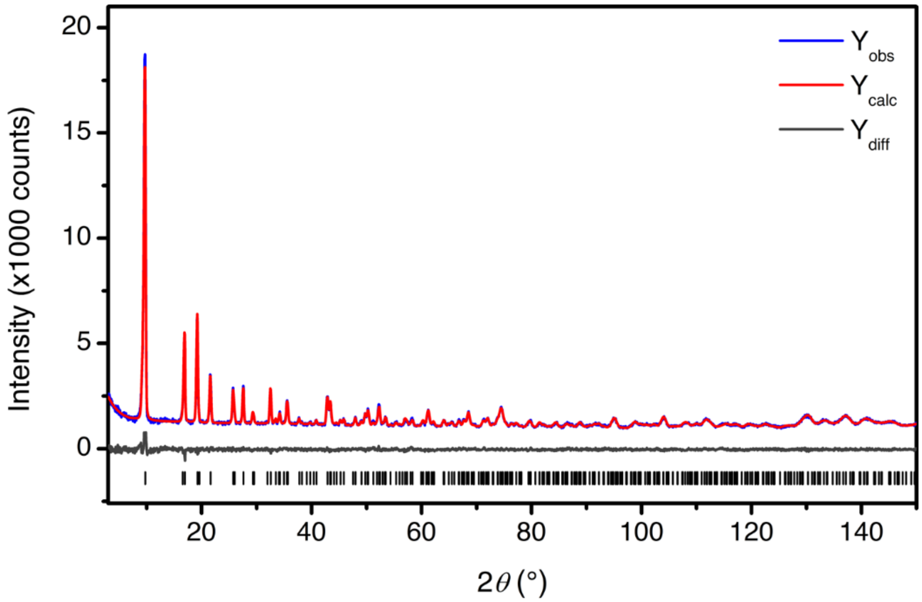

Supplementary Figure 37. Rietveld refinement using neutron powder diffraction data of desolvated $Co_2(OH)_2$(bbta) dosed with 1 equivalent of O_2, obtained at 8 K with a wavelength of 2.0772 Å, from 5º to 150º (blue line). The red line represents the calculated diffraction pattern, the gray line represents the difference between observed and calculated patterns, and the black tick marks indicate calculated Bragg peak positions. Figures-of-merit (as defined by GSAS): R_{wp} = 2.93%, R_p = 2.50%, R_{exp} = 2.81%, R_{Bragg} = 4.48%, GoF = 1.07.

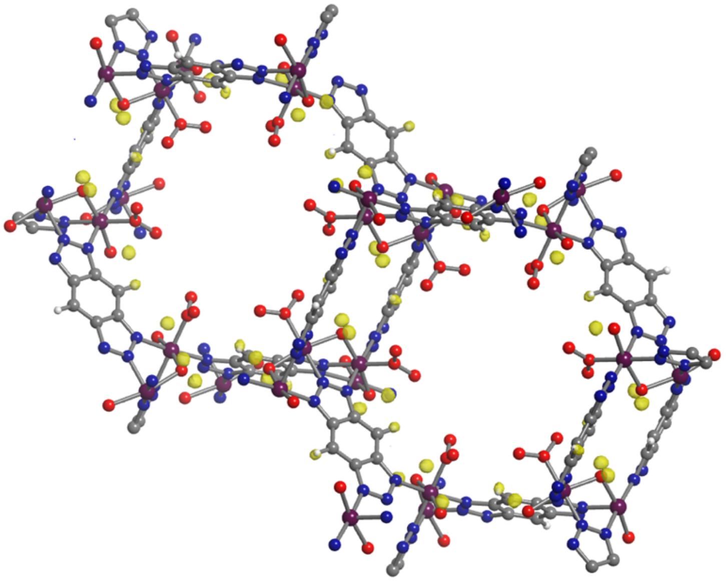

Supplementary Figure 38. View of the negative component of the Fourier difference map ($F_{obs} - F_{calc}$; shown in yellow) against the structural model of O_2-dosed $Co_2(OH)_2$(bbta) along the *c*-axis, as generated by the program VESTA.[4] The structural model was determined from Rietveld refinement with powder neutron diffraction data of the O_2-dosed sample (Supplementary Figure 37) with the hydroxo H atom omitted from the refinement. Two orientations are visible for the disordered superoxo group and the atomic positions of the hydroxo group visible in the difference map due to the nearby two-fold axis. Purple, red, blue, and gray spheres represent Co, O, N, C, and H atoms respectively.

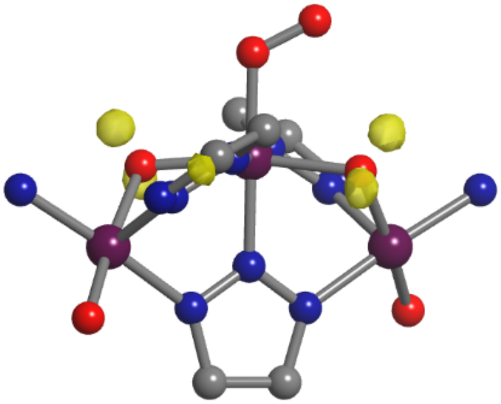

Supplementary Figure 39. View of the negative component of the Fourier difference map ($F_{obs} - F_{calc}$; shown in yellow) on the structural model of O_2-dosed $Co_2(OH)_2$(bbta) along the *c*-axis, as generated by the program VESTA.[4] The structural model was determined from Rietveld refinement with powder neutron diffraction data of the O_2-dosed sample (Supplementary Figure 37), with the hydroxo hydrogen atom omitted from the refinement. Only one orientation of the disordered superoxo group is shown for clarity, though two orientations are visible for the atomic position of the hydroxo group visible in the difference map due to the nearby two-fold axis. Purple, red, blue, and gray spheres represent Co, O, N, C, and H atoms respectively.

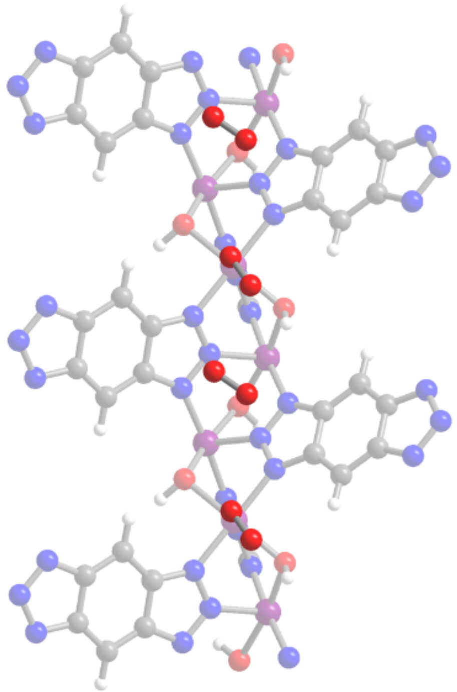

Supplementary Figure 40. View of the structural model of O_2-dosed $Co_2(OH)_2$(bbta) along the *c*-axis, as determined from Rietveld refinement with powder neutron diffraction data of the O_2-dosed sample (Supplementary Figure 37). The framework structure is faded to provide greater visibility to the adsorbed O_2 species, including the superoxo species (first and third from the bottom) and physisorbed oxygen (second and fourth from the bottom). Only one orientation of the disordered OH and superoxo groups is shown for clarity. Purple, red, blue, gray, and white spheres represent Co, O, N, C, and H atoms respectively.

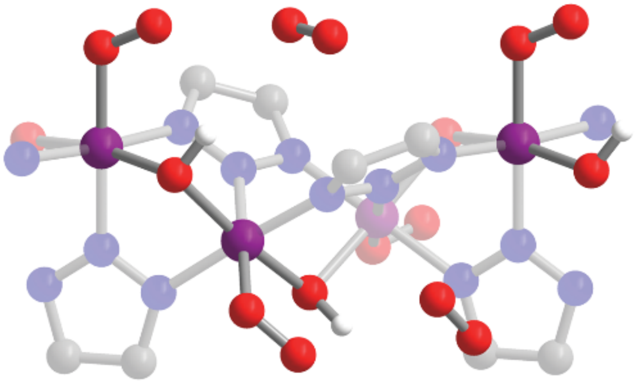

Supplementary Figure 41. View of the structural model of O_2-dosed $Co_2(OH)_2$(bbta) along the c-axis, as determined from Rietveld refinement with powder neutron diffraction data of the O_2-dosed sample (Supplementary Figure 37). The framework structure is lightened in color to provide greater visibility to the adsorbed O_2 species and hydrogen bonding interactions involving the superoxos and physisorbed oxygen. Only one orientation of the disordered OH and superoxo groups is shown for clarity. Purple, red, blue, gray, and white spheres represent Co, O, N, C, and H atoms respectively.

8. Hill equation analysis

Supplementary Table 17. Parameters obtained from applying the Hill equation analysis to different experimental datasets for $Co_2(OH)_2(bbta)$. The residual sum of squares (RSS) and the average relative error (ARE) are also reported with each associated fit.

Parameter	PXRD occupancies	Gas adsorption data (0 to 0.05 bar O_2)
n	0.262	0.514
K_d	0.371	0.0577
Saturation Capacity (mmol/g)	–	3.759
RSS	0.0005	0.027
ARE	0.00006	-0.004

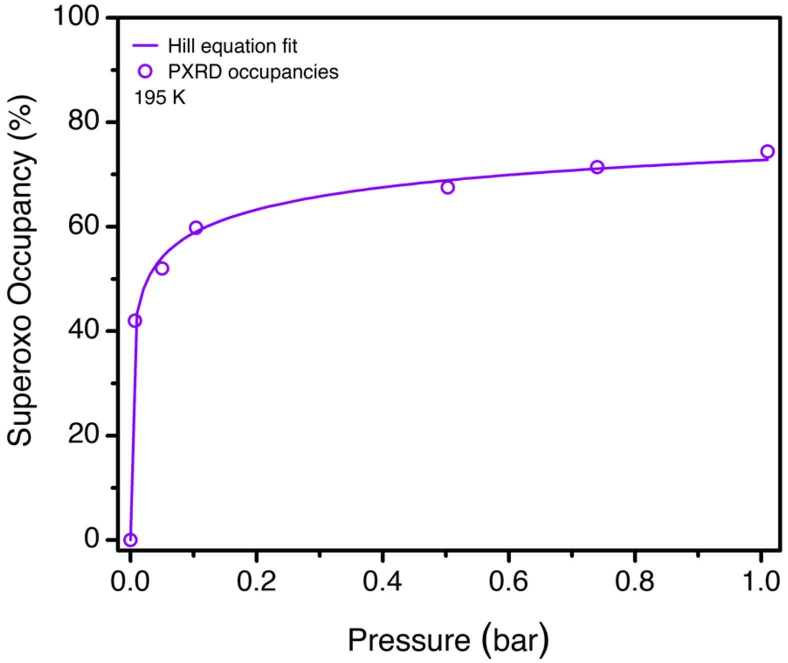

Supplementary Figure 42. Hill equation fit (purple line) to the occupancies of the superoxo species, determined from powder X-ray diffraction data obtained at 195 K (purple symbols).

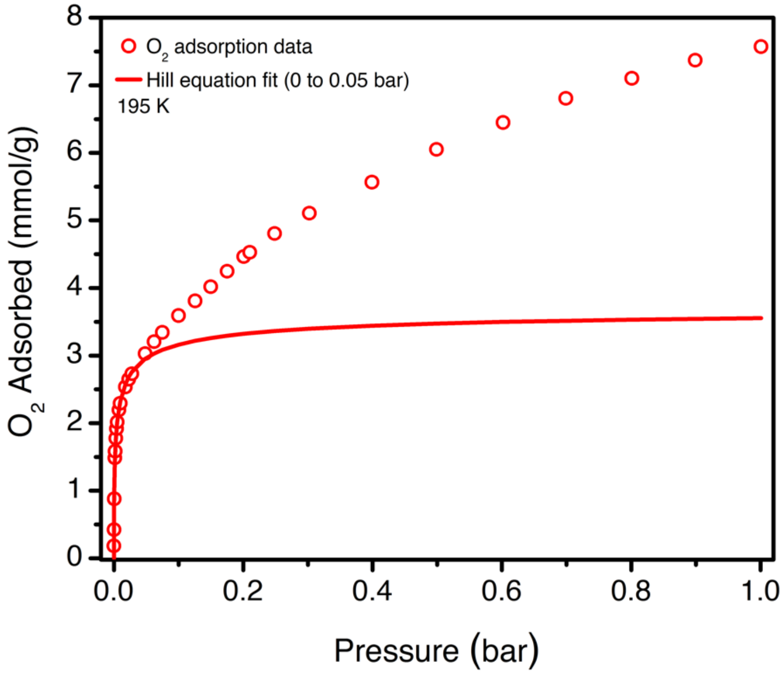

Supplementary Figure 43. Hill equation fit (red line) to the O_2 adsorption data of $Co_2(OH)_2(bbta)$ between 0 and 0.05 bar, obtained at 195 K (red symbols).

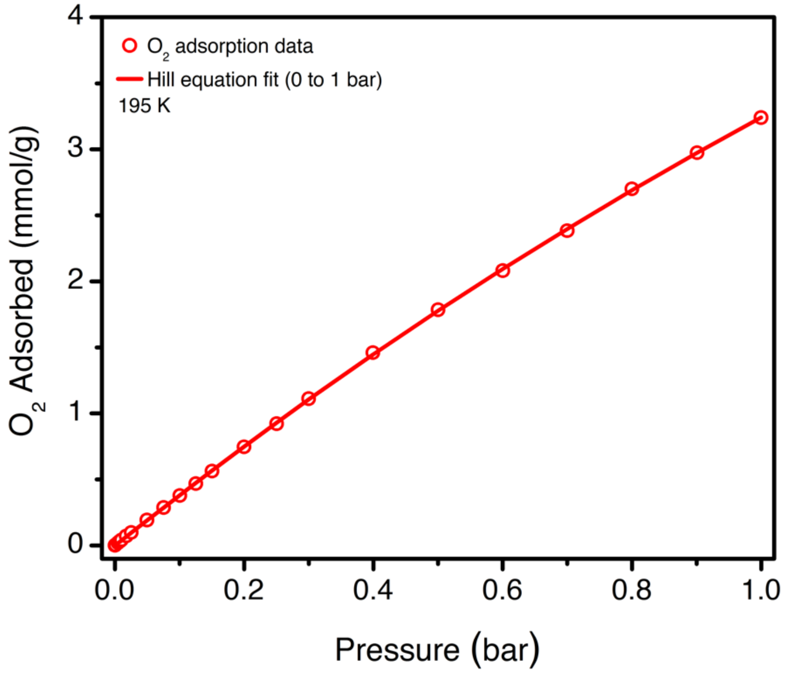

Supplementary Figure 44. Hill equation fit (red line) to the O_2 adsorption data of $Co_2Cl_2(bbta)$ between 0 and 1 bar, obtained at 195 K (red symbols). In this fit, q_{sat}, n, and K_d refine to 16.9 mmol/g, 1.01, and 4.2 respectively, with an RSS value of 0.00096 and an ARE value of 0.0146.

9. Scanning electron microscopy

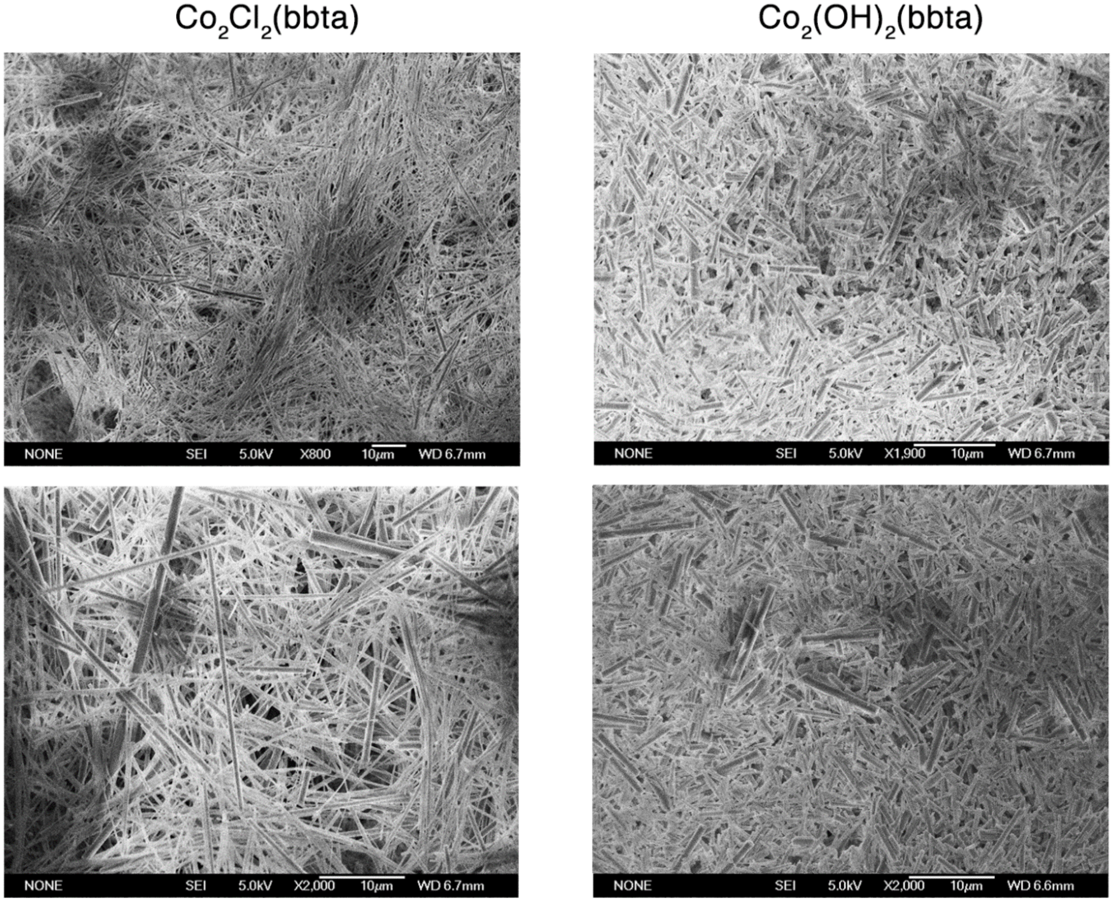

Supplementary Figure 45. Scanning electron microscopy images of Co_2Cl_2(bbta) (left) and $Co_2(OH)_2$(bbta) (right) crystallites. Scale bars at the bottom of the image indicate 10 μm. Crystals were imaged at 5 keV/12 μA by field emission SEM (JEOL FSM6430). Desolvated samples were suspended in methanol and drop cast onto a silicon chip. To dissipate charge, the samples were sputter coated with approximately 3 nm of Au (Denton Vacuum, LLC).

10. Diffuse reflectance infrared Fourier transform spectroscopy

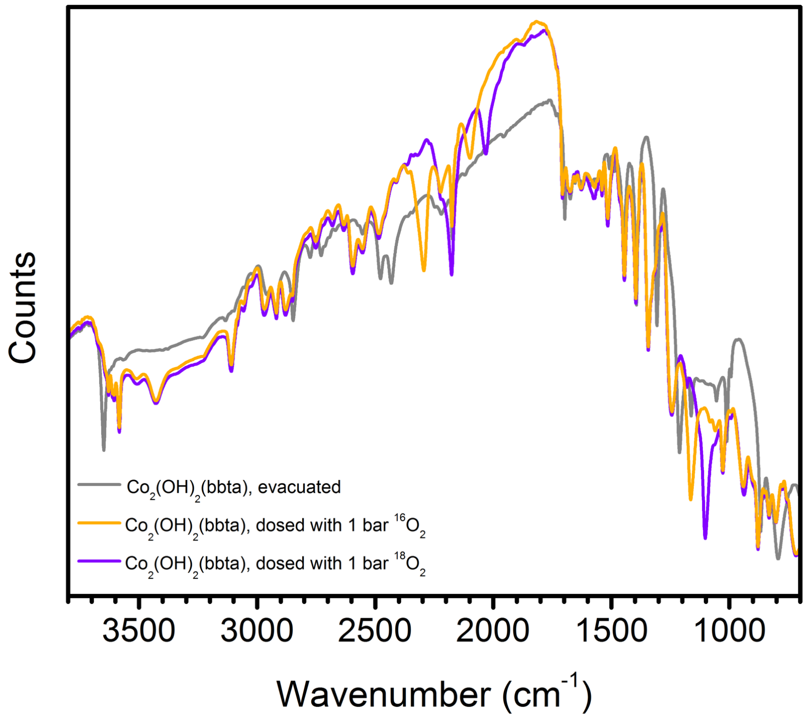

Supplementary Figure 46. Raw DRIFTS spectra (obtained at 195 K) of desolvated $Co_2(OH)_2$(bbta) under vacuum (gray trace), $Co_2(OH)_2$(bbta) dosed with 1 bar of $^{16}O_2$ (yellow trace), and $Co_2(OH)_2$(bbta) dosed with 1 bar of $^{18}O_2$ (purple trace).

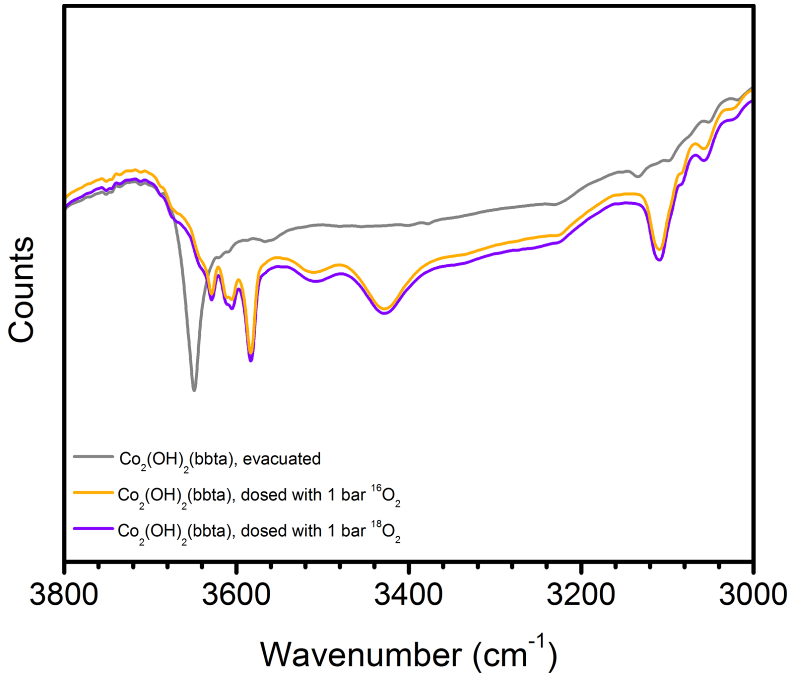

Supplementary Figure 47. Raw DRIFTS spectra (obtained at 195 K) of desolvated $Co_2(OH)_2$(bbta) under vacuum (gray trace), $Co_2(OH)_2$(bbta) dosed with 1 bar of $^{16}O_2$ (yellow trace), and $Co_2(OH)_2$(bbta) dosed with 1 bar of $^{18}O_2$ (purple trace). The broad band at 3420 cm^{-1} likely represents a Co(III)–OH species that participates in hydrogen bonding to superoxo species on the same Co(III) center.

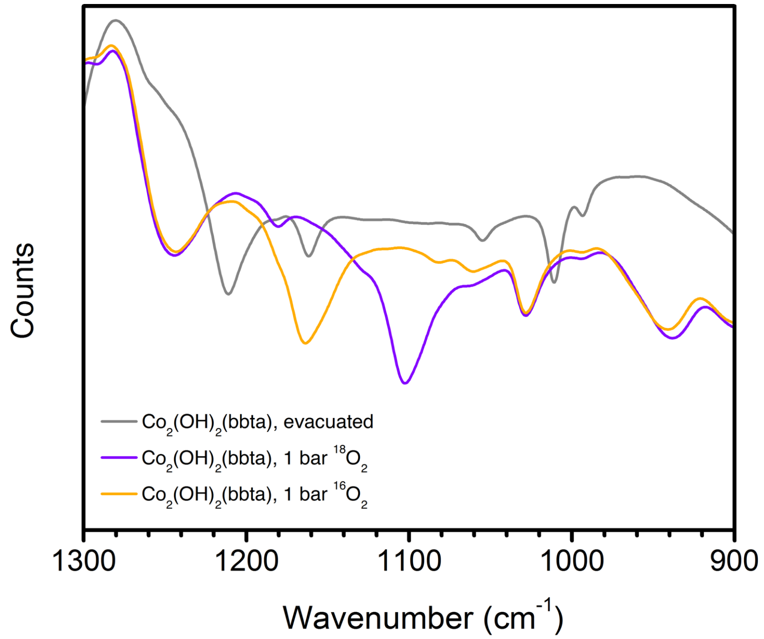

Supplementary Figure 48. Raw DRIFTS spectra (obtained at 195 K) of $Co_2(OH)_2$(bbta) under vacuum (gray trace), $Co_2(OH)_2$(bbta) dosed with 1 bar of $^{18}O_2$ (purple trace), and $Co_2(OH)_2$(bbta) dosed 1 bar of $^{16}O_2$ (orange-yellow trace). The ν(O–O) band observed at 1164 cm^{-1} in a bar of $^{16}O_2$ shifts to 1094 cm^{-1} in the presence of $^{18}O_2$ under similar conditions.

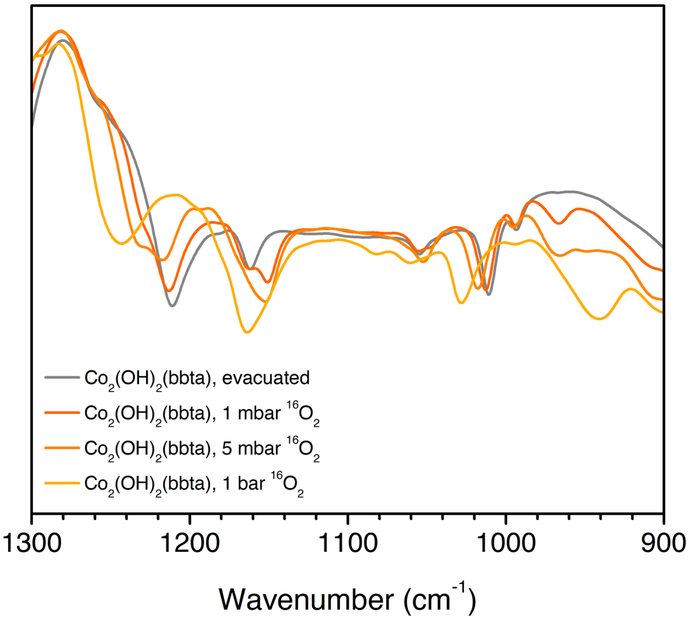

Supplementary Figure 49. Raw DRIFTS spectra of desolvated $Co_2(OH)_2$(bbta) under vacuum (gray trace) and $Co_2(OH)_2$(bbta) dosed with 1 mbar of $^{16}O_2$ (dark orange trace), 5 mbar of $^{16}O_2$ (orange trace), and 1 bar of $^{16}O_2$ (yellow trace). All spectra were obtained at 195 K. A superoxo ν(O–O) band is observed at 1150−1164 cm^{-1}, which shifts from lower wavenumbers to higher wavenumbers and grows in intensity with increasing pressure.

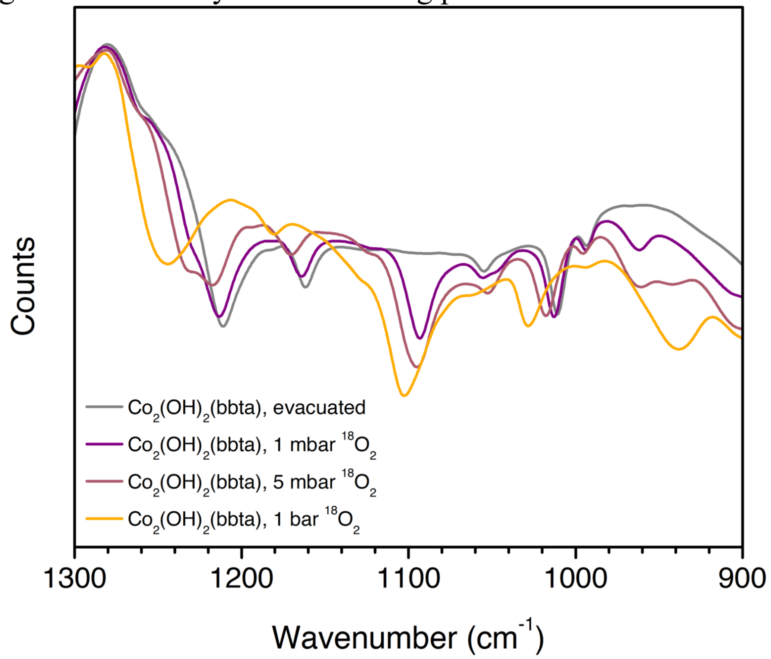

Supplementary Figure 50. Raw DRIFTS spectra (obtained at 195 K) of desolvated $Co_2(OH)_2$(bbta) under vacuum (gray trace) and $Co_2(OH)_2$(bbta) dosed with 1 mbar of $^{18}O_2$ (purple trace), 5 mbar of $^{18}O_2$ (magenta trace), and 1 bar of $^{18}O_2$ (yellow-orange trace). A superoxo ν(O–O) band is observed from 1092-1102 cm^{-1}, which shifts to higher wavenumbers and grows with increasing pressure. This allows observation of superoxo ν(O–O) free from interference by the framework peak at 1160 cm^{-1} in the evacuated spectrum.

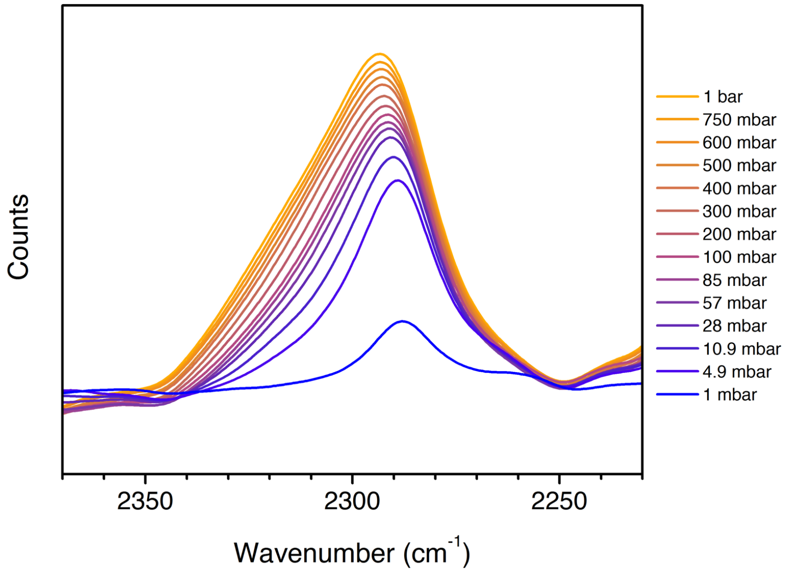

Supplementary Figure 51. DRIFTS spectra of $Co_2(OH)_2$(bbta) dosed with increasing pressures of $^{16}O_2$ (1 mbar, blue line, to 1 bar, orange line) at 195 K, subtracted from the spectrum of evacuated $Co_2(OH)_2$(bbta). The peak in the range of the figure is assigned to the superoxo ν(O–O) overtone band at 1150–1160 cm^{-1}. As can be seen in the fundamental ν(O–O) data, the overtone stretch shifts from a lower wavenumber to a higher wavenumber at higher pressures of O_2 dosed. Additionally, at all pressures, the intensity of the overtone increases, indicating formation of more superoxo species.

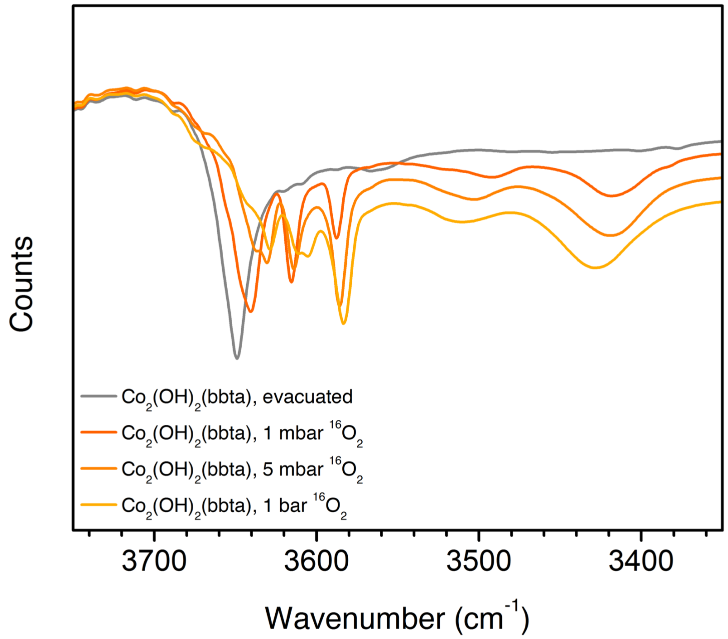

Supplementary Figure 52. Raw DRIFTS spectra of desolvated $Co_2(OH)_2$(bbta) under vacuum (gray trace) and $Co_2(OH)_2$(bbta) dosed with 1 mbar of $^{16}O_2$ (dark orange trace), 5 mbar of of $^{16}O_2$ (orange trace), and 1 bar of $^{16}O_2$ (yellow trace). All spectra were obtained at 195 K. At 3416-3427 cm^{-1} can be observed a broad *ν*(OH) band assigned as a cobalt(III)-hydroxo hydrogen bonding to the proximal O of the adsorbed cobalt-superoxo species.

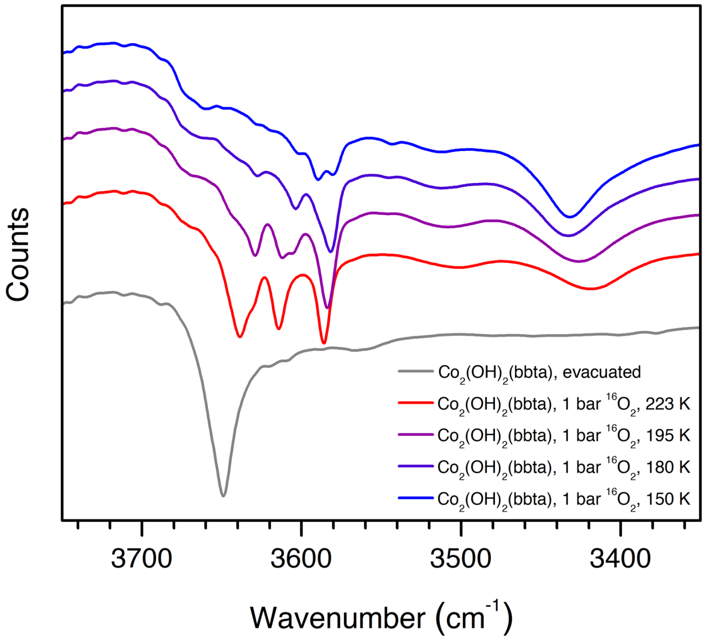

Supplementary Figure 53. Raw DRIFTS spectra of desolvated $Co_2(OH)_2$(bbta) (gray trace), $Co_2(OH)_2$(bbta) dosed with 1 bar of $^{16}O_2$ at 223, 195, 180, and 150 K (red, purple, violet, and blue traces, respectively). As superoxo binding sites proceed to full occupancy with lower temperatures, complex behavior is observed in isolated OH species (ν(OH) 3650–3575 cm^{-1}). At the highest occupancies, the dominant OH species is assigned to the hydrogen-bonding OH (ν(OH) 3430 cm^{-1}) with trace isolated OH remaining.

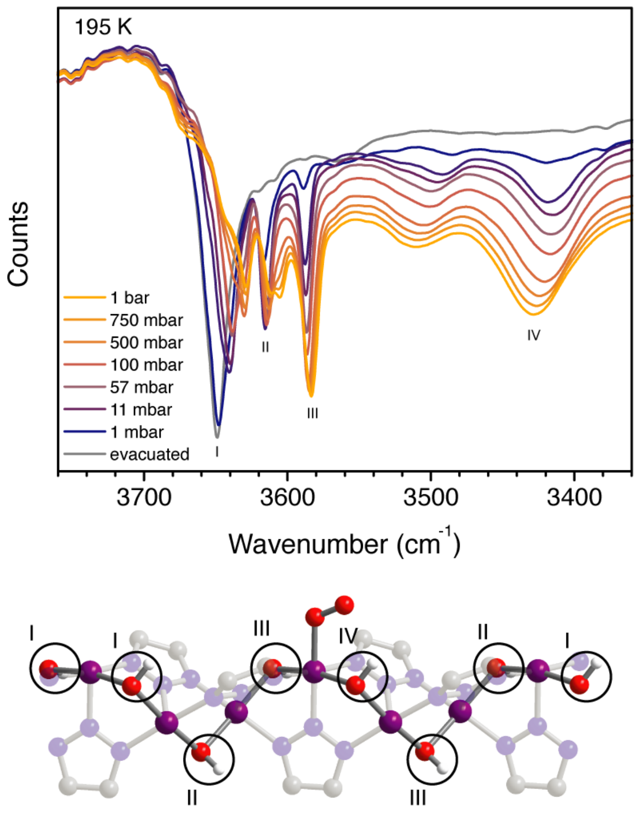

Supplementary Figure 54. Top: raw DRIFTS spectra obtained at 195 K for desolvated and $^{16}O_2$-dosed $Co_2(OH)_2$(bbta), similar to as shown in Figure 4a of the main text. Bottom: a view of the cobalt centers of the framework, with the cobalt centers and hydroxo ligands emphasized for clarity. Hydroxo groups along the chain are assigned to peaks I–III, based on comparisons to cobalt(II)-hydroxo and cobalt(III)-hydroxo complexes in the literature[5–8]. The broad ν(OH) band from 3416–3427 cm^{-1} (IV) is assigned to a cobalt(III)-hydroxo hydrogen bonded to the proximal oxygen atom of an adsorbed superoxo species. Sharper peaks (I-III) correspond to additional framework hydroxo groups, as indicated in the view of the framework.

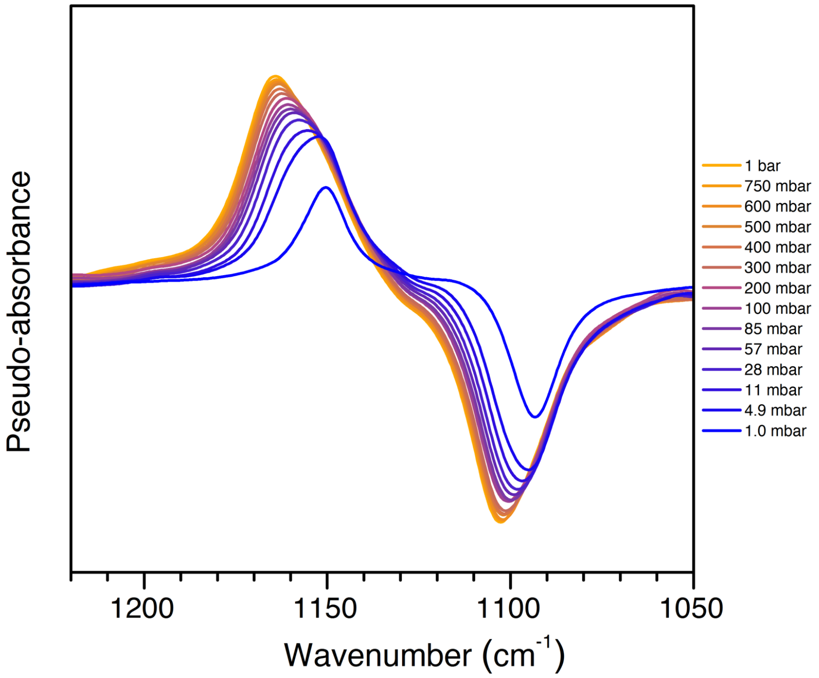

Supplementary Figure 55. DRIFTS spectra of $Co_2(OH)_2$(bbta) at 195 K dosed with increasing pressures of $^{16}O_2$ (1 mbar, blue line, to 1 bar, orange line), subtracted from the spectra of $Co_2(OH)_2$(bbta) dosed with equal pressures of $^{18}O_2$. The *v*(O–O) band of the adsorbed superoxo species appears as a positive peak for $^{16}O_2$ and a negative peak for $^{18}O_2$.

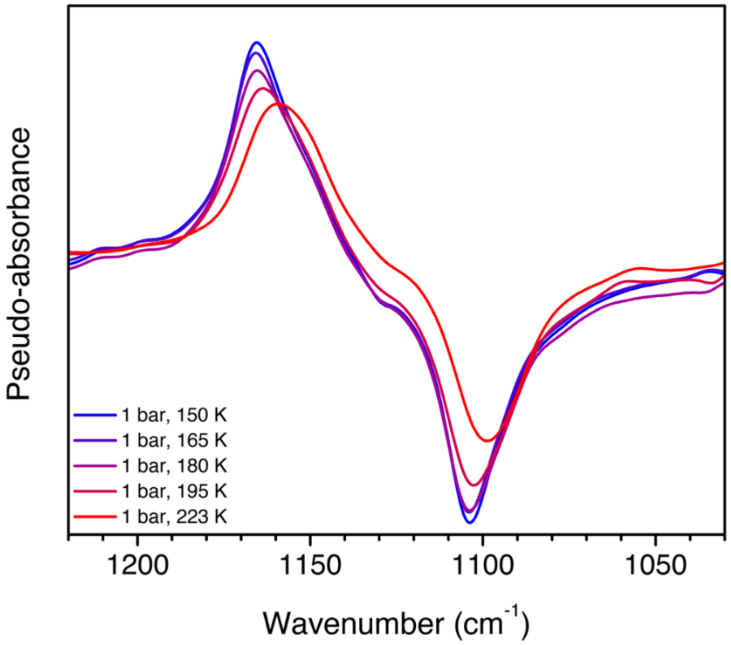

Supplementary Figure 56. DRIFTS spectra of $Co_2(OH)_2$(bbta) dosed with 1 bar of $^{16}O_2$ subtracted from the spectrum of $Co_2(OH)_2$(bbta) dosed with 1 bar of $^{18}O_2$, when taken at temperatures between 223 K and 150 K (red to blue lines). The peak in the range of the figure is assigned to the superoxo *v*(O–O) band. The fundamental *v*(O–O) data shifts from a lower wavenumber to a higher wavenumber at lower temperatures.

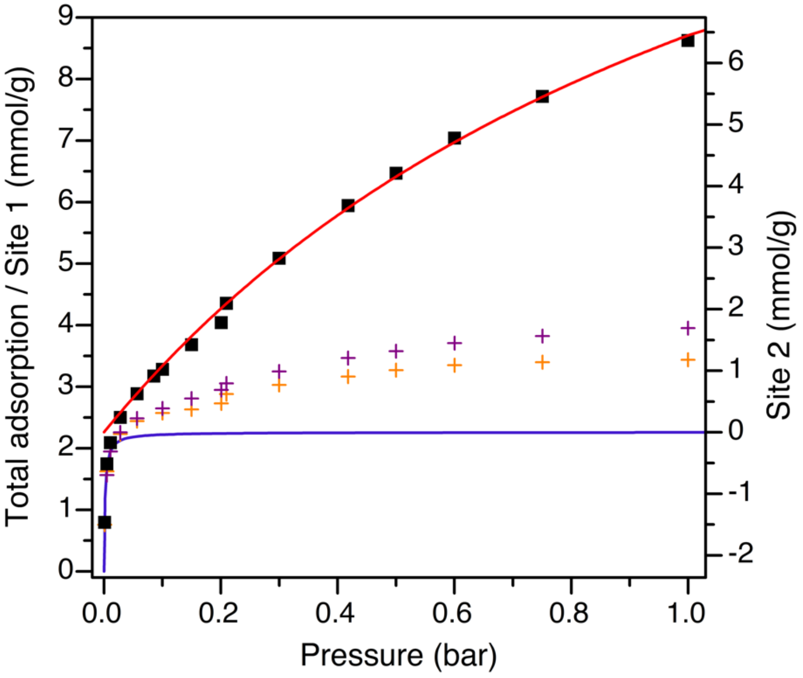

Supplementary Figure 57. A plotted comparison of the O_2 adsorption isotherm data at 195 K, obtained from the isotherm run concurrently while measuring DRIFTS spectra on $Co_2(OH)_2$(bbta) (black symbols), to the expected contribution of the two sites fit by dual-site Langmuir modelling of the variable temperature adsorption data (blue line, the primary strong adsorption site; red line, the secondary adsorption site; see Supplementary Information Section 4). Additionally, the integrated areas of the superoxo fundamental and overtone (orange and purple symbols respectively) are shown. All IR areas have been normalized to the theoretical strong site uptake at 10.93 mbar, showing that even after the primary site has presumably saturated, there is still substantial growth in the intensity of the superoxo species.

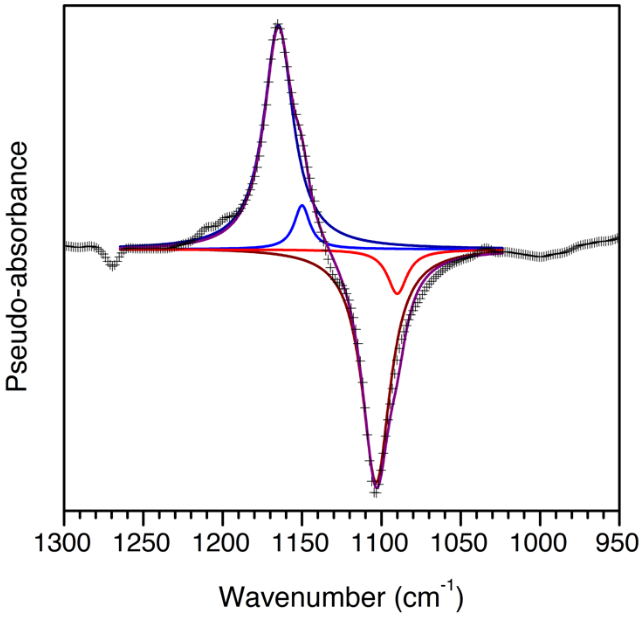

Supplementary Figure 58. 150 K DRIFTS spectrum of $Co_2(OH)_2$(bbta) dosed with 1 bar of $^{16}O_2$ subtracted from the spectrum of $Co_2(OH)_2$(bbta) dosed with 1 bar of $^{18}O_2$, showing asymmetric bands assigned to the superoxo *v*(O–O) (black tick marks). The difference spectrum could be fit to four Lorentzian peaks (purple trace), yielding two contributions each for the $^{16}O_2$ (red and magenta) and $^{18}O_2$ (dark and slate blue) contributions. The dominant species under these conditions appears at 1164.9 cm^{-1} with $^{16}O_2$ and 1103.3 cm^{-1} with $^{18}O_2$.

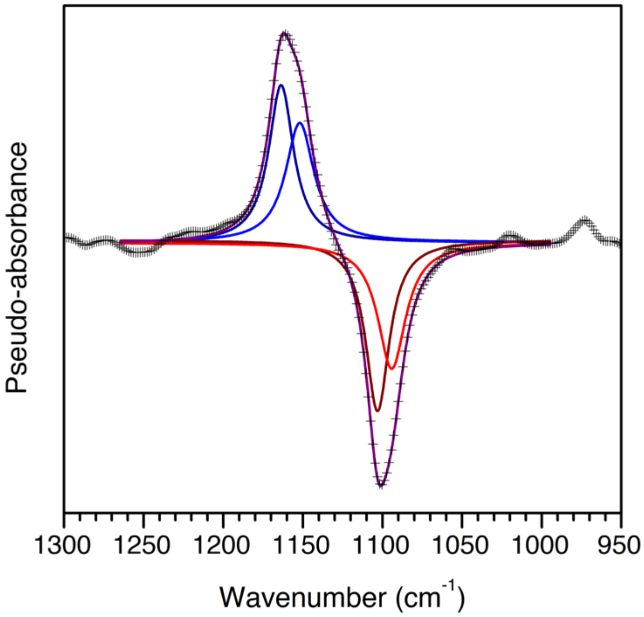

Supplementary Figure 59. 150 K DRIFTS spectrum of $Co_2(OH)_2$(bbta) dosed with 200 mbar of $^{16}O_2$ subtracted from the spectrum of $Co_2(OH)_2$(bbta) dosed with 200 mbar of $^{18}O_2$, showing slightly asymmetric bands assigned to the superoxo *v*(O–O) (black tick marks). The difference spectrum could be fit to four Lorentzian peaks (purple trace), yielding two contributions each for the $^{16}O_2$ (red and magneta) and $^{18}O_2$ (dark and slate blue) at 1163.7 / 1152.0 cm^{-1} and 1103.1 / 1094.2 cm^{-1}, respectively.

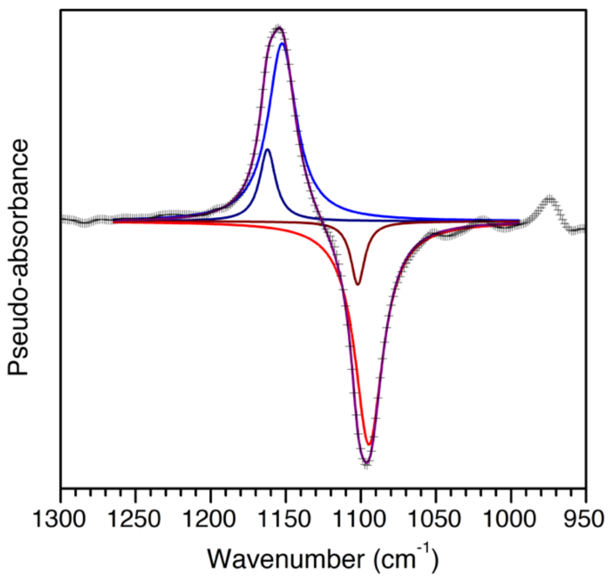

Supplementary Figure 60. 223 K DRIFTS spectra of $Co_2(OH)_2$(bbta) dosed with 300 mbar of $^{16}O_2$ subtracted from the spectrum of $Co_2(OH)_2$(bbta) dosed with 300 mbar of $^{18}O_2$, showing asymmetric bands assigned to the superoxo *v*(O–O) (black tick marks). Fitting the bands to four Lorentzian peaks (purple trace) revealed two components for $^{16}O_2$ (red and magenta) and $^{18}O_2$ (dark and slate blue). The predominant species is the lower wavenumber band (1152.5 and 1094.6 cm^{-1} for $^{16}O_2$ and $^{18}O_2$, respectively).

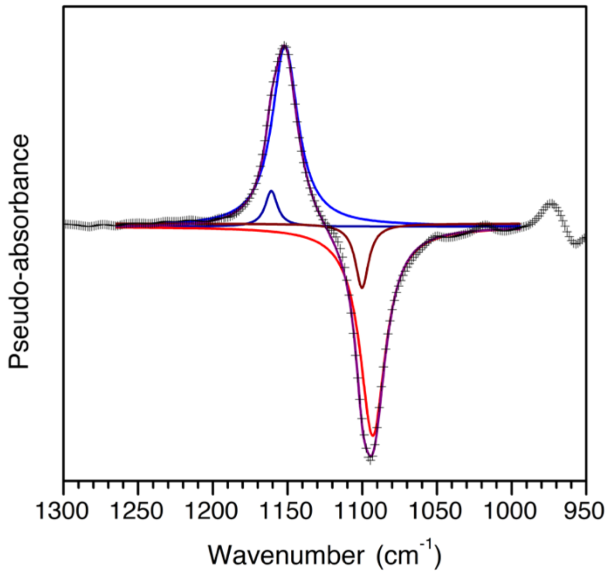

Supplementary Figure 61. 223 K DRIFTS spectra of $Co_2(OH)_2$(bbta) dosed with 100 mbar of $^{16}O_2$ subtracted from the spectrum of $Co_2(OH)_2$(bbta) dosed with 100 mbar of $^{18}O_2$, revealing asymmetric bands assigned to the superoxo *v*(O–O) (black tick marks). A total fit to the data with four Lorentzian peaks (purple trace) revealed two contributions each for $^{16}O_2$ (red and magenta) and $^{18}O_2$ (dark and slate blue). The predominant species is the lower wavenumber band (1151.8 cm^{-1} for $^{16}O_2$ and 1092.9 cm^{-1} for $^{18}O_2$).

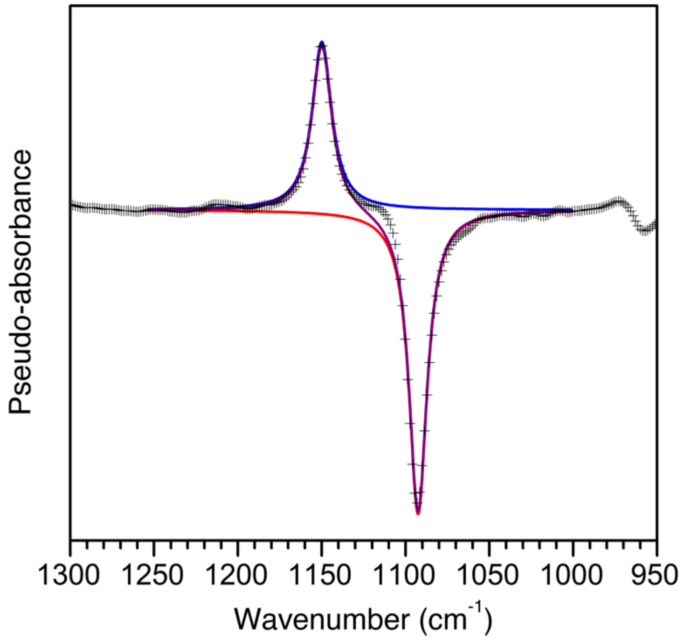

Supplementary Figure 62. 223 K DRIFTS spectra of $Co_2(OH)_2$(bbta) dosed with 5 mbar of $^{16}O_2$ subtracted from the spectrum of $Co_2(OH)_2$(bbta) dosed with 5 mbar of $^{18}O_2$, revealing major bands assigned to the superoxo *v*(O–O) (black tick marks). The fitting of the band to two Lorentzian peaks (purple trace) resulted in an excellent fit with one component each for $^{16}O_2$ (red trace, 1149.9 cm^{-1}) and $^{18}O_2$ (blue trace, 1092.6 cm^{-1}).

11. Measurements of d.c. magnetic susceptibility

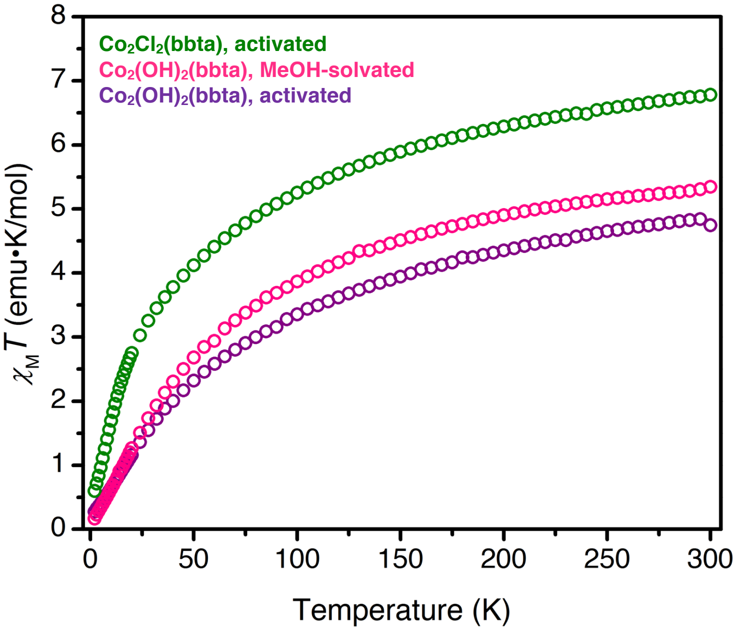

Supplementary Figure 63. Variable temperature magnetic susceptibility times temperature ($\chi_M T$ vs. T) data of samples of desolvated Co_2Cl_2(bbta) (green symbols), desolvated $Co_2(OH)_2$(bbta) (purple symbols), and methanol-solvated $Co_2(OH)_2$(bbta) (pink symbols). All samples were measured under an applied dc field of 0.1 T.

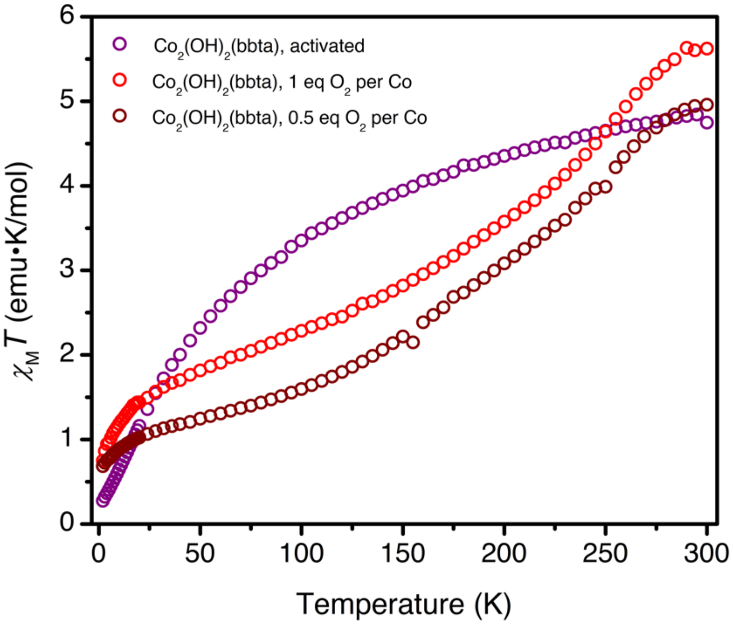

Supplementary Figure 64. Variable temperature magnetic susceptibility times temperature ($\chi_M T$ vs. T) data of samples of $Co_2(OH)_2$(bbta) desolvated (purple symbols), dosed with 0.5 equivalent of O_2 (maroon symbols), and dosed with 1 equivalent of O_2 (red symbols). All samples were measured under an applied dc field of 0.1 T.

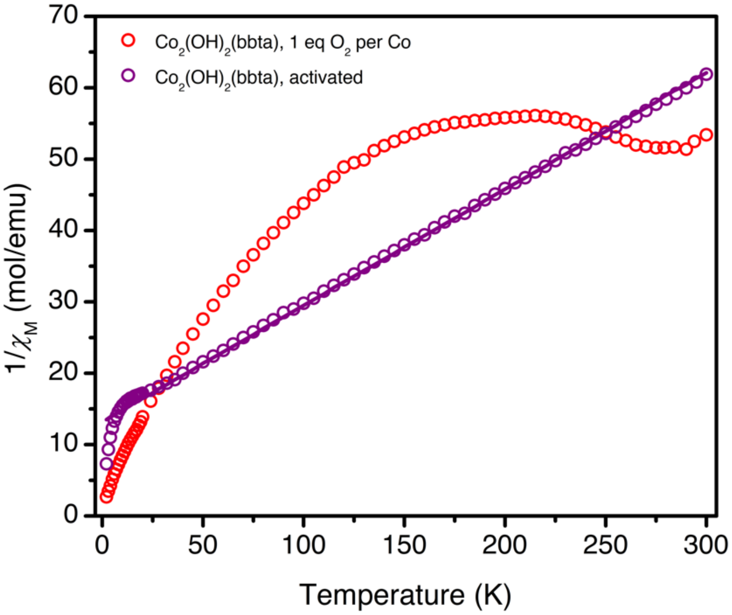

Supplementary Figure 65. The inverse of variable temperature molar magnetic susceptibility ($1/\chi_M$ vs. T) data of samples of $Co_2(OH)_2$(bbta) desolvated (purple symbols) and dosed with 1 equivalent of O_2 (red symbols). All samples were measured under an applied dc field of 0.1 T. A Curie-Weiss fit to the high temperature data of desolvated $Co_2(OH)_2$(bbta) is shown by a solid purple line and afforded C = 6.13 emu K mol^{-1} and $\theta_{CW} = -81.0$ K.

12. Supplementary Methods: Periodic calculations

The periodic models used in the calculations for the activated Co_2Cl_2(bbta) and $Co_2(OH)_2$(bbta) were obtained from the corresponding crystallographic cells determined experimentally, composed by nine formula units ($Co_{18}Cl_{18}(bbta)_9$ and $Co_{18}(OH)_{18}(bbta)_9$). All the periodic structures were optimized using the Perdew-Burke-Ernzerhof (PBE) functional[9] as implemented in the VASP 5.4.4 program.[10] Long-range dispersion interactions were considered using the scheme proposed by Grimme (D3).[11] The plane wave energy cutoff was set to 520 eV, while the Brillouin zone was sampled at the Gamma point. Convergence thresholds were set to 10^{-6} eV for the energy and to 0.02 eV/Å for the forces. Lattice parameters and atom positions were fully optimized using a conjugate-gradient algorithm. No symmetry constraints were imposed (symmetry P_1). Loadings of one O_2 molecule and 18 O_2 molecules per formula unit were considered ($Co_2(OH)_2$(bbta)–O_2 and $Co_2(OH)_2$(bbta)–18O_2, respectively), corresponding to Co:O_2 ratios of 0.05 and 1, respectively. Experimentally, the O_2 isosteric heat of adsorption on $Co_2(OH)_2$(bbta) is dependent on the coverage. For $Co_2(OH)_2$(bbta), we also examined the adsorption of two molecules on two neighboring Co sites, considering four different geometries in order to gain information on the evolution of the adsorption process with coverage (see Supplementary Figure 84): (1) two O_2 adsorbed on the same Co chain (Co1 and Co2 in Supplementary Figure 83a, 2O_2-sfdp); (2,3) two O_2 adsorbed on Co on two neighboring chains (Co1 and Co3, 2O_2-dfsp-a and 2O_2-dfsp-b); (4) two O_2 adsorbed on Co on two neighboring chains, being the two Co not one in front of the other (Co1 and Co4, 2O_2-dfdp). To differentiate between the relative position of the two O_2, we use the shorthand s = same, d = different, p = pore and f = Mg–O file. Geometries (1) and (4) correspond to O_2 adsorbed in two different channels, while in (2) and (3) the two O_2 molecules are in the same pore. An additional structure was considered where the second O_2 molecule is not interacting with a Co center but it is interacting with a previously coordinated O_2 (1O_2-2OH-1O_2-ontop). The PBE-D3 functional is unable to predict correctly the energy of the spin ladder for the isolated MOFs, as expected because of its local character.[12–14] For this reason, single point calculations using PBE0-D3 functional[15] have been performed on the PBE-D3 optimized structures to obtain the adsorption energies and analyze the electronic properties of the material.

13. Supplementary Methods: Cluster calculations

The models used to determine the electronic and magnetic properties of the MOFs were carved from the experimental crystal structure of Co_2Cl_2(bbta) and $Co_2(OH)_2$(bbta) (this work) considering four metals atoms and six organic linkers having final formula $[Co_4(OH)_5(C_6N_6H_2)_6]^{-9}$ and $[Co_4Cl_5(C_6N_6H_2)_6]^{-9}$, respectively (named in the following **Co_4OH** and **Co_4Cl** clusters, see Supplementary Figure 66a and b). A chain of four Co was considered to keep the central Co atoms free of influence from possible boundary conditions and saturation choices of the clusters. Two Co is the minimal necessary number of metal atoms to evaluate the preferential ferro- or antiferromagnetic configuration on the MOFs. The charge was neutralized by the addition of 9 protons. These protons were symmetrically added to cap the triazolate linkers on the positions that were cropped on the periodic structure, in order to avoid unbalanced charge localization. The models possess a final formula of $Co_4(Cl)_5(C_6N_6H_4)_3(C_6N_6H_3)_3$ for Co_2Cl_2(bbta) and $Co_4(OH)_5(C_6N_6H_4)_3(C_6N_6H_3)_3$ for $Co_2(OH)_2$(bbta). Additionally, dicobalt cluster models were designed by replacing the external Co atoms of the prior tetracobalt **Co_4OH** and **Co_4Cl** clusters by Mg^{2+} ions, named as **Co_2Mg_2Cl** and **Co_2Mg_2OH** (Supplementary Figure 66c and d). Partial geometry optimizations were conducted by relaxing C and H atoms whereas the key atoms (Co,

N, O or Cl) were kept fixed as the experimentally observed positions. Since the magnetic properties are dependent on geometries,[16] the distances and angles of Co, N, O and Cl of the MOF framework were kept fixed to those determined by Rietveld refinement of the powder X-ray diffraction data. The hybrid M06 Minnesota functional[17] in the unrestricted formalism (UM06), as implemented in the *Gaussian 09* software package,[18] was employed to describe all possible spin and electron configuration, together with the magnetic coupling constants determined from good performance in previous benchmark studies on MOFs and inorganic complexes.[16,19–21] Additional calculations were performed in order to compared the UM06 performance against the PBE0[9] and B3LYP[22,23] hybrid functionals functional[17] in the unrestricted formalism (UPBE0 and UB3LYP). The def2-TZVP basis set was employed for all atoms.[24,25]

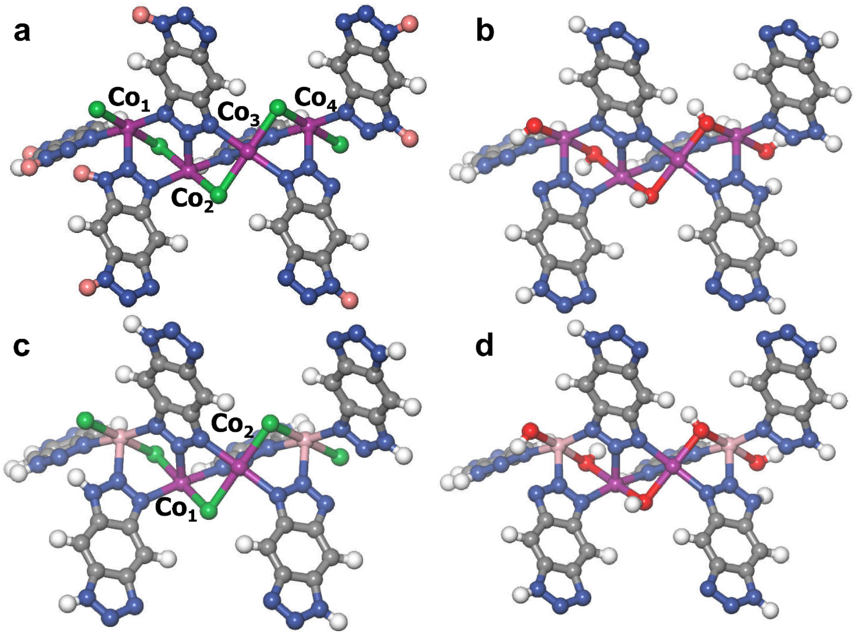

Supplementary Figure 66. **a**, **Co_4Cl**, **b**, **Co_4OH**, **c**, **Co_2Mg_2Cl** and **d**, **Co_2Mg_2OH** cluster model as optimized at the UM06/def2-TZVP level considering three unpaired alpha electrons for Co. The nine protons used to saturate the cluster are indicated in pink for the **Co_4Cl** cluster, and the Mg atoms added to the Co_2Mg_2Cl and Co_2Mg_2OH clusters are indicated in pale pink. Purple, green, grey, blue, red, and white spheres represent Co, Cl, C, N, O, and H atoms, respectively.

Complete active space self-consistent field (CASSCF) single point calculations followed by second-order perturbation theory (CASPT2) were performed using the MOLCAS 8.2 program[26] at UM06 optimized geometries for the state with the highest spin multiplicity. A triple-ζ quality basis set was used for Co (ANO-RCC-VTPZ) whereas a double-ζ quality was used for N, O and Cl in the first coordination sphere (ANO-RCC-VDPZ) and a minimal ANO-RCC-MB basis set was used for the remaining atoms. The active space choices are indicated as (n,m) where n is the number of active electrons and m is the number of active orbitals. In the choice of the active space, we have considered that in these materials, each Co has a square-pyramid-like geometry in the activated material and a square-bipyramid-like when the material is interacting with adsorbates (e.g., O_2 or solvent molecules). Thus, it is expected that the $3d_{xz}$ and $3d_{yz}$ orbitals on each cobalt atom are doubly occupied and the $3d_{xy}$, $3d_{z2}$, and $3d_{x2-y2}$ are singly occupied (see Supplementary Figure 67) and the latter will play a more important role during the active space selection. A

minimal active space of six electrons in six orbitals containing the cobalt singly occupied 3*d*-orbitals (indicated as CAS(6,6)) was used for the Co_2Mg_2Cl and Co_2Mg_2OH clusters. Each of these orbitals is the result of the linear combination with equal coefficients of one 3*d* orbital from each Co atom, with negligible contribution from the ligands for this active space. A larger active space of (6,12), including metal–ligand orbitals and second shell effects, was also considered to explore the active space convergence. For the tetracobalt systems (Co_4OH and Co_4Cl), a CAS(12,12) was selected as a minimal active space considering three 3*d* orbitals per cobalt atom.

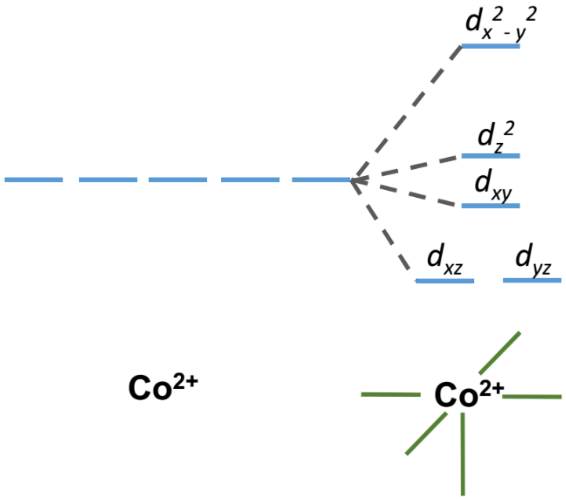

Supplementary Figure 67. Schematic representation of the molecular orbital diagram for the 3*d* orbitals of a cobalt ion in a square-pyramid-like ligand field.

An additional set of cluster models was obtained from the optimized periodic structures in order to model the changes in the vibrational frequencies of O_2 and of the frameworks after adsorption. These new models are fully equivalent to the tetracobalt clusters reported in Supplementary Figure 66a and b. In the first step, only the position of all the H atoms was optimized at the UM06 level in Gaussian 09 using the def2-TZVP basis set for Co and def2-SVP for all the other atoms. The spin ladder was obtained by performing single point calculations on these structures. For the most stable spin state a further optimization step was conducted where the Co atoms, the first coordination sphere of the two central Co (including the hydroxo groups in the case of $Co_2(OH)_2$(bbta)), and the O_2 molecules were allowed to relax. For these atoms, def2-TZVP basis sets were used while def2-SVP basis sets were used for all the others. This level of calculations has shown to be able to correctly reproduce the experimental O_2 adsorption energies in Co-based frameworks in a previous study.[16] Harmonic frequencies were computed using analytic second derivatives in order to evaluate the shift in the vibrational frequency of the O_2 molecule and of the –OH groups upon adsorption. No scaling factors were adopted. Charges were obtained using the Charge Model 5 population analysis (CM5) while the spin densities (expressed as the difference between the α and β electron densities) were evaluated using the Hirshfeld population analysis.[27,28]

14. Markov chain simulations of negative cooperativity

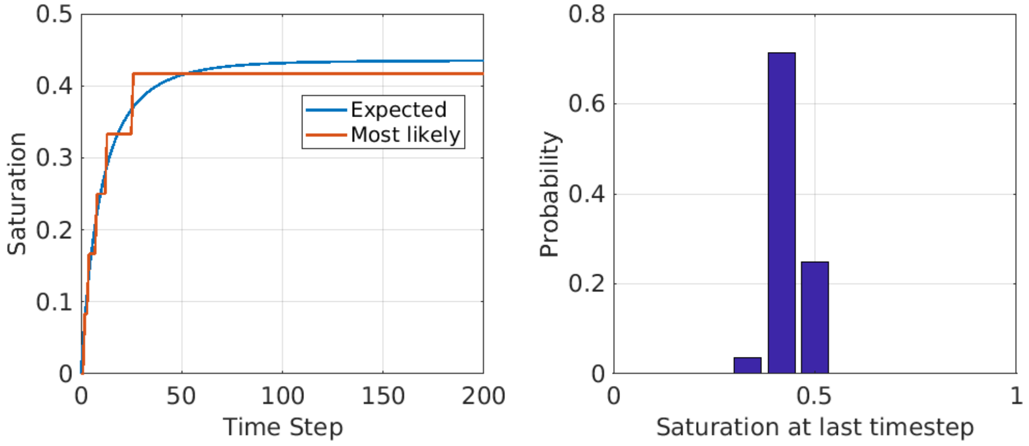

Supplementary Figure 68. Expected and most likely saturation of a twelve-Co chain at timesteps up to T = 200 (left), and probability of different saturations at the final timestep (right). At the final timestep, the expected saturation is about 43%. The expected saturation is asymptotically sub-logarithmic with respect to time; doubling the number of timesteps gives less than a 0.5% increase in final expected saturation.

15. Supplementary Methods: Computed electronic and magnetic properties of Co_2Cl_2(bbta) and $Co_2(OH)_2$(bbta)

Validation of the Co4OH and Co4Cl models

The CM5 charges, Hirshfeld spin density, and electrostatic potential (ESP) maps analysis were used to investigate how the replacement of the two external Co atoms with Mg affect the properties on the dicobalt cluster compared to the tetra-cobalt clusters. We observed in Supplementary Tables 18 and 19 that the two central Co present almost identical charges and spin densities for both models and ligands (**Co_4L** and the **Co_2Mg_2L** cluster, L = OH and Cl**)**. From this analysis, we expect that the dicobalt model will be able to represent the metal-metal coupling present in the material. In addition, the electrostatic potential contour maps were found to be identical for the selected truncation of both **Co_4L** and the **Co_2Mg_2L** models as shown in Supplementary Figure 69.

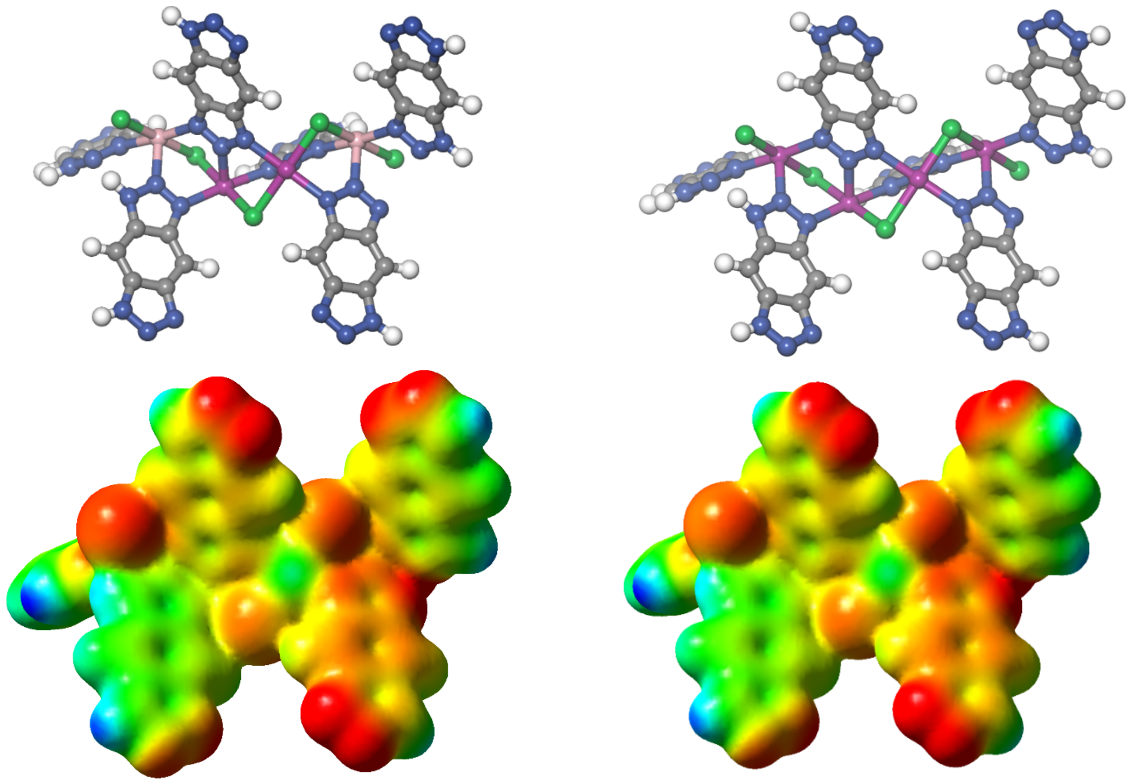

Co_2Mg_2Cl **Co_4Cl**

Supplementary Figure 69. Electrostatic potential maps of the clusters used to simulate Co_2Cl_2(bbta) (**Co_2Mg_2Cl** S=3 and **Co_4Cl** S = 6) as optimized at the UM06/def2-TZVP level and by freezing the position of Co, Mg, Cl and N to the values determined experimentally in this work. Contour color code from red (−0.06) to blue (+0.205) (isosurface: 0.005 au). Purple, pink, green, dark blue, gray, and white spheres represent Co, Mg, Cl, N, C, and H atoms, respectively.

Supplementary Table 18. Charges and spin densities on Co for Co_2Cl_2(bbta) as obtained at the UM06/def2-TZVP level for the **Co_4Cl** (2S + 1 = 13) and **Co_2Mg_2Cl** (2S + 1 = 7) clusters. CM5 charge (q_{CM5}) and Hirshfeld spin density on Co (ρ_{Co}). All the values are reported in a.u.

**Co_4Cl**	q_{CM5}	ρ_{Co}	**Co_2Mg_2Cl**	q_{CM5}	ρ_{Co}
Co1	0.54	2.67	**Mg1**	-	-
Co2	0.58	2.69	**Co2**	0.49	2.66
Co3	0.57	2.70	**Co3**	0.49	2.66
Co4	0.52	2.64	**Mg4**	-	-

Supplementary Table 19. Charges and spin densities on Co for $Co_2(OH)_2$(bbta) as obtained at the UM06/def2-TZVP level for the **Co_4OH** (2S + 1 = 13) and **Co_2Mg_2OH** (2S + 1 = 7) clusters. CM5 charge (q_{CM5}) and Hirshfeld spin density on Co (ρ_{Co}). All the values are reported in a.u.

**Co_4OH**	q_{CM5}	ρ_{Co}	**Co_2Mg_2OH**	q_{CM5}	ρ_{Co}
Co1	0.54	2.67	**Mg1**	-	-
Co2	0.57	2.68	**Co2**	0.57	2.68
Co3	0.56	2.69	**Co3**	0.56	2.68
Co4	0.52	2.63	**Mg4**	-	-

Spin ladder for Co_2Cl_2(bbta)

Co_2Mg_2Cl Cluster Model

In Supplementary Tables 20–22, we report the results obtained using the UM06, UPBE0 and UB3LYP density functionals, respectively. The three approximations predict a similar relative stability of the different spin states (see Supplementary Table 23). By considering only pure spin states, the highest possible multiplicity ($2S$+1 = 7) is predicted to be the most stable state, suggesting that all the Co atoms are in a high spin state. Inspection of the spin densities shows that about 2.6 unpaired electron are located on each Co. The pure singlet state is predicted to be the highest in energy lying at least 200 kJ mol^{-1} above the ground state.

At the UM06 level, the antiferromagnetic (AF) configuration obtained from the broken-symmetry solution was also calculated for the singlet spin state (BS-1 in Supplementary Tables 20 and 23). This configuration is slightly more stable than the high spin septet by about 0.7 kJ mol^{-1}. This is in agreement with the experiments that indicate the antiferromagnetic as the most stable configuration. In Supplementary Figure 70, the spin density map shows an antiferromagnetic configuration with three unpaired electrons located on each Co.

Supplementary Table 20. M06 Spin ladder for Co_2Mg_2Cl. Relative energy stability (ΔE, in kJ mol^{-1}), Hirshfeld spin density on Co (ρ_{Co}) and on all the other atoms ($\rho_{no\text{-}metal}$) as obtained at the **UM06**/def2-TZVP level for the **Co_2Mg_2Cl** cluster for different spin multiplicities (2S + 1). The theoretical value for S^2 ($S(S+1)_{theo}$) along with those obtained in the calculations before ($S(S+1)_{before}$) and after ($S(S+1)_{after}$) the default correction of Gaussian09 for the higher spin state is also reported.

**$2S$+1**	$S(S+1)_{theo}$	$S(S+1)_{before}$	$S(S+1)_{after}$	ρ_{Co1}	ρ_{Co2}	$\rho_{no\text{-}metal}$	ΔE
1	0	0.00	0.00	0.00	0.00	0.00	334.5
3	2	2.19	2.01	0.97	0.97	0.06	112.0
5	6	6.06	6.00	0.97	2.66	0.37	75.1
7	12	12.03	12.00	2.66	2.66	0.68	0.0
**BS-1**	**0**	**3.03**	**8.63**	**2.73**	**-2.72**	**-0.01**	**-0.7**

Supplementary Table 21. PBE0 Spin ladder for Co_2Mg_2Cl. Relative energy stability (ΔE, in kJ mol^{-1}), Hirshfeld spin density on Co (ρ_{Co}) and on all the other atoms ($\rho_{no\text{-}metal}$) as obtained at the **UPBE0**/def2-TZVP level for the **Co_2Mg_2Cl** cluster for different spin multiplicities (2S + 1). The theoretical value for S^2 ($S(S+1)_{theo}$) along with those obtained in the calculations before ($S(S+1)_{before}$) and after ($S(S+1)_{after}$) the default correction of Gaussian09 for the higher spin state is also reported.

**2*S*+1**	***S*(*S*+1)$_{theo}$**	***S*(*S*+1)$_{before}$**	***S*(*S*+1)$_{after}$**	**ρ_{Co1}**	**ρ_{Co2}**	**$\rho_{no\text{-}metal}$**	**Δ*E***
1	0	0.00	0.00	0.00	0.00	0.00	382.2
3	2	2.02	2.00	0.98	0.99	2.00	122.1
5	6	6.02	6.00	0.99	2.69	0.33	52.9
**7**	**12**	**12.02**	**12.00**	**2.68**	**2.68**	**0.59**	**0.0**

Supplementary Table 22. B3LYP Spin ladder for Co_2Mg_2Cl. Relative energy stability (ΔE, in kJ mol^{-1}), Hirshfeld spin density on Co (ρ_{Co}) and on all the other atoms ($\rho_{no\text{-}metal}$) as obtained at the **UB3LYP**/def2-TZVP level for the **Co_2Mg_2Cl** cluster for different spin multiplicities (2S + 1). The theoretical value for S^2 ($S(S+1)_{theo}$) along with those obtained in the calculations before ($S(S+1)_{before}$) and after ($S(S+1)_{after}$) the default correction of Gaussian09 for the higher spin state is also reported.

**2*S*+1**	***S*(*S*+1)$_{theo}$**	***S*(*S*+1)$_{before}$**	***S*(*S*+1)$_{after}$**	**ρ_{Co1}**	**ρ_{Co2}**	**$\rho_{no\text{-}metal}$**	**Δ*E***
1	0	0.00	0.00	0.00	0.00	0.00	297.6
3	2	2.56	2.14	0.97	0.97	0.07	79.5
5	6	6.02	6.00	0.98	2.64	0.38	40.3
**7**	**12**	**12.02**	**12.00**	**2.65**	**2.66**	**0.69**	**0.0**

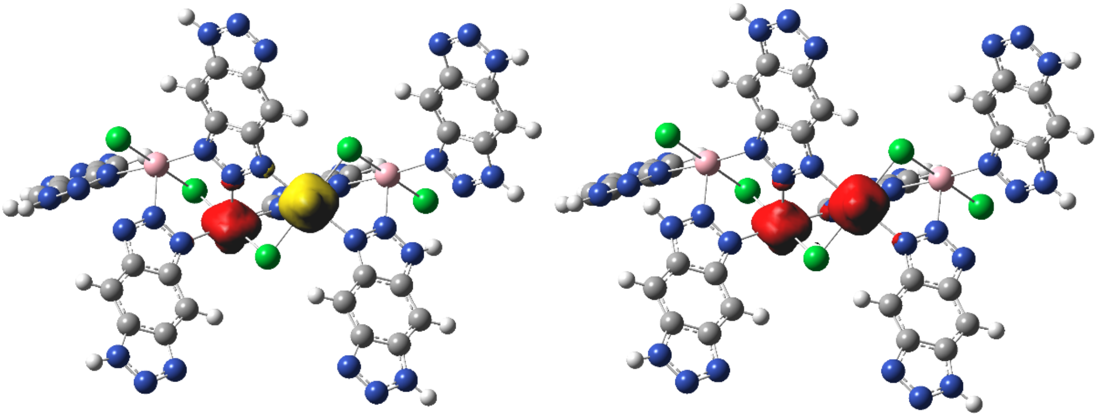

Supplementary Figure 70. Spin density on Co_2Mg_2Cl for S = 0 (left, antiferromagnetic configuration) and S = 3 (right, high-spin ferromagnetic configuration) as optimized at the UM06/def2-TZVP level. Red density corresponds to alpha electron excess, yellow density to beta electron excess (isosurface: 0.01 au). Atoms labeled according to the color code: Co (purple), Cl (green), Mg (pink), N (blue), C (gray), H (white).

Supplementary Table 23. Co_2Mg_2Cl Spin ladder obtained by DFT methods. Relative energy stability (kJ mol^{-1}) obtained at the unrestricted UM06, UPBE0, and UB3LYP/def2-TZVP level of theory for the **Co_2Mg_2Cl** cluster for increasing spin multiplicities (2S + 1).

2*S*+1	M06	PBE0	B3LYP
1	334.5	382.2	297.6
3	112.0	122.1	79.5
5	75.1	52.9	40.3
7	**0.0**	**0.0**	**0.0**
BS-1	-0.7	N/A	N/A

Multireference calculations were performed using a minimal active space of (6,6) (Supplementary Table 24) and an enlarged active space of (6,12) (Supplementary Table 25). The results obtained with the two active spaces are similar, indicating that a correct modeling of the multiconfigurational character of the system can be achieved with the minimal active space. The calculations indicate that the open shell $S = 0$ state is the ground state at both CASSCF and CASPT2 level, in agreement with UM06 (the broke-symmetry configuration BS-1 in Supplementary Table 23). The singlet wave function for $S = 0$ is a linear combination of fourteen different electronic configurations having similar configuration interaction (CI) coefficients. However, all of them consider the six active orbitals to be singly occupied with three α electrons and three β electrons without any preferential configuration.

Supplementary Table 24. CAS(6,6) Spin ladder for Co_2Mg_2Cl. Relative energy stability (kJ mol^{-1}) obtained at the CASSCF and CASPT2 level of theory using a (6,6) active space for the **Co_2Mg_2Cl** cluster for increasing spin multiplicities (2S + 1).

2*S*+1	CASSCF(6,6)	CASPT2
1	**0.00**	**0.00**
**3**	0.03	1.47
**5**	0.07	2.22
**7**	1.84	5.29

Supplementary Table 25. CAS(6,12) Spin ladder for Co_2Mg_2Cl. Relative energy stability (kJ mol^{-1}) obtained at the CASSCF and CASPT2 level of theory using a (6,12) active space for the **Co_2Mg_2Cl** cluster for increasing spin multiplicities (2S + 1).

2*S*+1	CASSCF(6,12)	CASPT2
**1**	0.00	0.00
**3**	0.03	1.14
**5**	0.09	1.50
**7**	0.17	-0.01

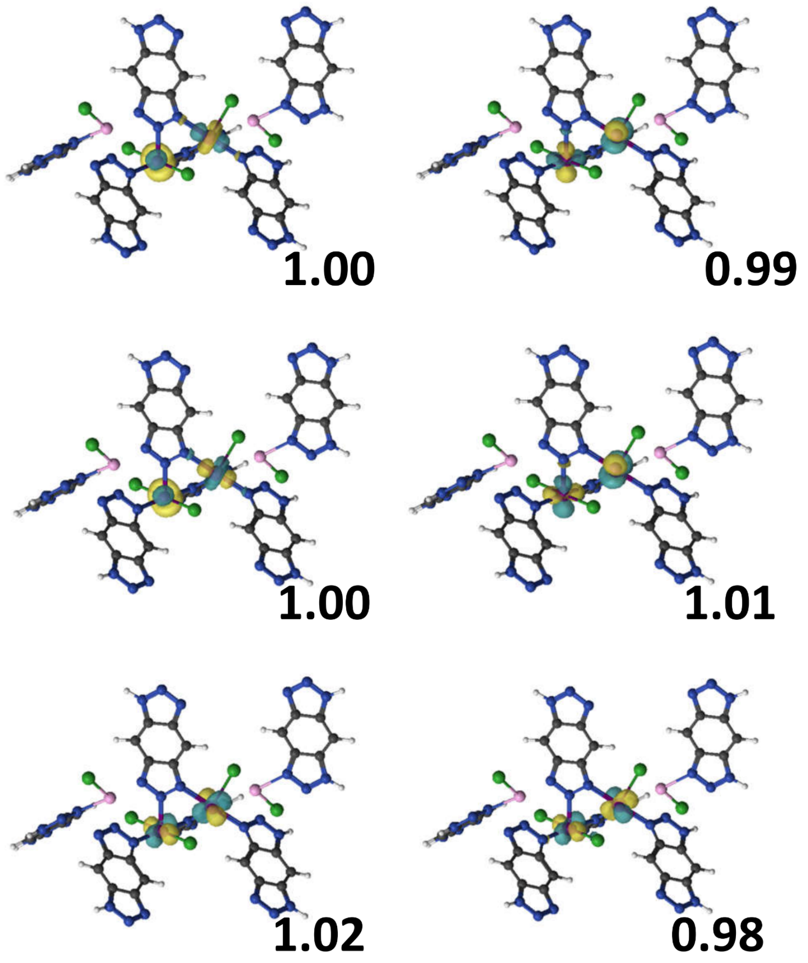

Supplementary Figure 71. Co 3d orbitals and natural orbital occupation numbers in Co_2Mg_2Cl computed with the (6,6) active space for the singlet state. Color code: Co (purple), Cl (green), C (grey), N (blue), O (red), H (white).

Co_4Cl Cluster Model

The highest spin state is predicted to be the ground state also in this case for all the hybrid Kohn Sham DFT methods used (see Supplementary Tables 26–29). The spin density plot for the high spin multiplicity $S = 13$ (Supplementary Figure 72) shows that also for this larger cluster the alpha unpaired electrons are mainly localized on the Co atoms, with a small fraction of the density sited on the coordinating nitrogen ligand atoms.

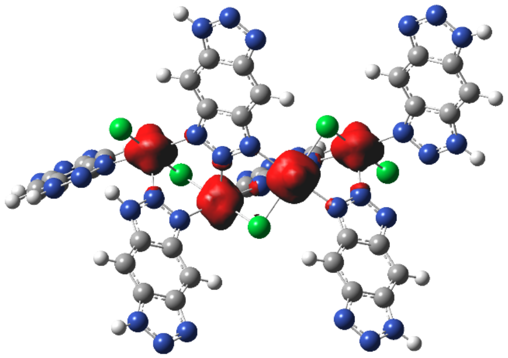

Supplementary Figure 72. Spin density plot on Co_4Cl for S = 6 as optimized at the UM06/def2-TZVP level and by freezing the position of Co, Cl and N to the values determined experimentally (this work). Red density corresponds to alpha electron excess, yellow density to beta electron excess (isosurface: 0.01 au). Atoms labeled according to the color code: Co (purple), Cl (green), N (blue), C (gray), H (white).

Three different calculations at the UM06-L level were performed by broken symmetry solution in order to allow the determination of three possible cobalt center configurations, denoted with a superscript in the notation below (high-spin, HS, and low-spin, LS, respectively). These configurations are BS-1-$\alpha^{HS}\beta^{HS}\alpha^{HS}\beta^{HS}$, BS-1-$\beta^{HS}\alpha^{HS}\alpha^{HS}\beta^{HS}$ and BS-1-$\alpha^{LS}\alpha^{HS}\beta^{HS}\beta^{LS}$. All of them are higher in energy with respect to the high spin configuration (S = 6). Nevertheless, the two configurations having all the cobalt atoms in high spin states are close in energy to the S = 6 energy by less than 10 kJ mol^{-1} (BS-1-$\alpha^{HS}\beta^{HS}\alpha^{HS}\beta^{HS}$ and BS-1-$\beta^{HS}\alpha^{HS}\alpha^{HS}\beta^{HS}$). On the other hand, the BS-1-$\alpha^{LS}\alpha^{HS}\beta^{HS}\beta^{LS}$ configuration is about 90 kJ mol^{-1} less stable. It is evident that the antiferromagnetic configurations with local high-spin cobalt are lower in energy than the configuration with local high and low spin cobalt mixed.

Supplementary Table 26. UM06 Spin ladder for Co_2Cl_2(bbta). Relative energy stability (ΔE, in kJ mol^{-1}), Hirshfeld spin density on Co (ρ_{Co}) and on all the other atoms ($\rho_{no\text{-}metal}$) as obtained at the **UM06**/def2-TZVP level for the **Co_4Cl** cluster at different spin multiplicities (2S + 1). The theoretical value for S^2 ($S(S+1)_{theo}$) along with those obtained in the calculations before ($S(S+1)_{before}$) and after ($S(S+1)_{after}$) the default correction of Gaussian 09 for the higher spin state is also reported.

**2*S*+1**	**$S(S+1)_{theo}$**	**$S(S+1)_{before}$**	**$S(S+1)_{after}$**	**ρ_{Co1}**	**ρ_{Co2}**	**ρ_{Co3}**	**ρ_{Co4}**	**$\rho_{no\text{-}metal}$**	**Δ*E***
1	0	0.00	0.00	0.00	0.00	0.00	0.00	0.00	648.3
3	2	5.06	9.27	0.99	2.66	-2.65	1.02	-0.01	108.6
5	6	-	-	-	-	-	-	-	-
7	12	-	-	-	-	-	-	-	-
9	20	20.28	20.01	0.99	2.66	0.97	2.65	0.73	95.5
11	30	30.16	30.00	2.64	2.67	0.98	2.66	1.04	72.3
**13**	**42**	**42.07**	**42.00**	**2.64**	**2.67**	**2.66**	**2.65**	**1.37**	**0.0**
BS-1-$\alpha^{HS}\beta^{HS}\alpha^{HS}\beta^{HS}$	0	6.06	18.77	2.64	-2.65	2.65	-2.65	0.01	9.4
BS-1-$\beta^{HS}\alpha^{HS}\alpha^{HS}\beta^{HS}$	0	6.06	18.76	-2.64	2.66	2.65	-2.66	-0.01	5.0
BS-1-$\alpha^{LS}\alpha^{HS}\beta^{HS}\beta^{LS}$	0	4.25	13.27	0.98	2.66	-2.65	-1.01	0.03	89.9

Supplementary Table 27. UPBE0 Spin ladder for Co_2Cl_2(bbta). Relative energy stability (ΔE, in kJ mol^{-1}), Hirshfeld spin density on Co (ρ_{Co}) and on all the other atoms ($\rho_{no\text{-}metal}$) as obtained at the **UPBE0**/def2-TZVP level for the **Co_4Cl** cluster at different spin multiplicities (2S + 1). The theoretical value for S^2 ($S(S+1)_{theo}$) along with those obtained in the calculations before ($S(S+1)_{before}$) and after ($S(S+1)_{after}$) the default correction of Gaussian 09 for the higher spin state is also reported.

**2*S*+1**	**$S(S+1)_{theo}$**	**$S(S+1)_{before}$**	**$S(S+1)_{after}$**	**ρ_{Co1}**	**ρ_{Co2}**	**ρ_{Co3}**	**ρ_{Co4}**	**$\rho_{no\text{-}metal}$**	**Δ*E***
1	0	0.00	0.00	0.00	0.00	0.00	0.00	0.00	787.0
3	2	3.05	2.10	1.00	0.99	-0.99	1.02	-0.01	266.4
5	6	6.05	6.00	1.00	0.99	0.99	1.02	0.01	266.9
7	12	12.04	12.00	1.00	0.99	0.99	2.68	0.34	204.0
9	20	20.04	20.00	2.68	0.99	0.99	2.68	0.66	132.3
11	30	30.04	30.00	2.68	0.99	2.68	2.69	0.96	70.3
**13**	**42**	**42.03**	**42.00**	**2.69**	**2.71**	**2.70**	**2.69**	**1.22**	**0.0**

Supplementary Table 28. UB3LYP Spin ladder for Co_2Cl_2(bbta). Relative energy stability (ΔE, in kJ mol^{-1}), Hirshfeld spin density on Co (ρ_{Co}) and on all the other atoms ($\rho_{no\text{-}metal}$) as obtained at the **UB3LYP**/def2-TZVP level for the **Co_4Cl** cluster at different spin multiplicities (2S + 1). The theoretical value for S^2 ($S(S+1)_{theo}$) along with those obtained in the calculations before ($S(S+1)_{before}$) and after ($S(S+1)_{after}$) the default correction of Gaussian 09 for the higher spin state is also reported.

**2*S*+1**	**$S(S+1)_{theo}$**	**$S(S+1)_{before}$**	**$S(S+1)_{after}$**	**ρ_{Co1}**	**ρ_{Co2}**	**ρ_{Co3}**	**ρ_{Co4}**	**$\rho_{no\text{-}metal}$**	**ΔE**
1	0	0.00	0.00	0.00	0.00	0.00	0.00	0.00	594.0
3	2	-	-	-	-	-	-	-	-
5	6	6.04	6.00	0.99	0.98	0.98	1.01	0.06	167.2
7	12	-	-	-	-	-	-	-	-
9	20	20.43	20.01	0.97	0.98	2.65	2.65	0.76	80.2
11	30	30.04	30.00	2.64	0.98	2.65	2.64	1.09	41.7
**13**	**42**	**42.04**	**42.00**	**2.64**	**2.66**	**2.65**	**2.65**	**1.40**	**0.0**

Supplementary Table 29. Relative energy stability (kJ mol^{-1}) obtained at the unrestricted UM06, UPBE0, and UB3LYP/def2-TZVP level of theory for the **Co_4Cl** cluster for increasing spin multiplicities (2S + 1).

**2*S*+1**	**M06**	**PBE0**	**B3LYP**
1	648.3	787.0	594.0
3	108.6	266.4	-
5	-	266.9	167.2
7	-	204.0	-
9	95.5	132.3	80.2
11	72.3	70.3	41.7
**13**	**0.0**	**0.0**	**0.0**
BS-1-$\alpha^{HS}\beta^{HS}\alpha^{HS}\beta^{HS}$	**9.4**	**-**	**-**

Multireference calculations suggest the singlet as the ground spin state for **Co_4Cl** at both the CASSCF and CASPT2 level (Supplementary Table 30). The singlet state is characterized by an antiferromagnetic configuration of the high spin Co, nearly degenerate with the corresponding ferromagnetic one. In this case, more than 2000 electronic configurations contribute to the singlet wave function for $S = 0$, all of them having a weight < 0.2% and 60 with a weight > 0.1%.

Supplementary Table 30. CAS(12,12) relative energy stability (kJ mol^{-1}) obtained at the CASSCF and CASPT2 level of theory using a (12,12) active space for the **Co_4Cl** cluster for increasing spin multiplicities (2S + 1).

2*S*+1	CASSCF(12,12)	CASPT2
**1**	**0.00**	**0.00**
3	0.15	0.20
5	0.10	1.62
7	0.05	1.08
9	0.05	1.64
11	0.05	3.19
13	0.07	3.77

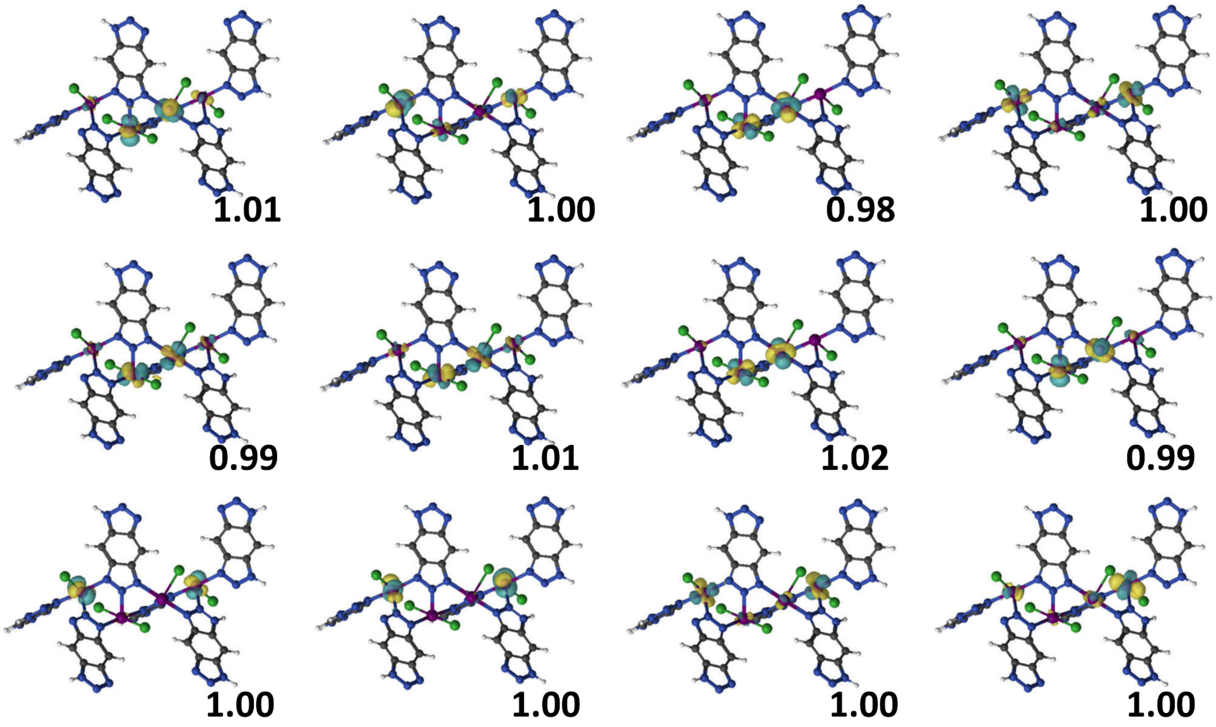

Supplementary Figure 73. Co 3d orbitals in Co_4Cl and the natural orbital occupation numbers computed with the (12,12) active space for the singlet state. Color code: Co (purple), Cl (green), C (grey), N (blue), O (red), H (white).

Spin ladder for $Co_2(OH)_2$(bbta)

Co_2Mg_2OH Cluster Model

The three DFT sets of calculations predict different relative stabilities of the spin states. Although all of them agree that the singlet is the least stable state, UM06 predicts the highest spin state to be the most stable one as already observed for $Co_2(OH)_2$(bbta), while UPBE0 and UB3LYP predict the quintet as the most stable state. The spin density map for the highest spin state (Supplementary

Figure 75) shows that the majority of the alpha density is located on the Co atoms, and the remaining part on the equatorial N and on the O atoms.

Supplementary Table 31. M06 Spin ladder for $Co_2(OH)_2$(bbta). Relative energy stability (ΔE, in kJ mol^{-1}), Hirshfeld spin density on Co (ρ_{Co}) and on all the other atoms ($\rho_{no\text{-}metal}$) as obtained at the **UM06**/def2-TZVP level for the **Co_2Mg_2OH** cluster for different spin multiplicities (2S + 1). The theoretical value for S^2 ($S(S+1)_{theo}$) along with those obtained in the calculations before ($S(S+1)_{before}$) and after ($S(S+1)_{after}$) the default correction of Gaussian 09 for the higher spin state is also reported.

2*S*+1	$S(S+1)_{theo}$	$S(S+1)_{before}$	$S(S+1)_{after}$	ρ_{Co1}	ρ_{Co2}	$\rho_{no\text{-}metal}$	Δ*E*
1	0	0.00	0.00	0.00	0.00	0.00	270.3
3	2	2.12	2.01	0.98	0.97	0.05	55.8
5	6	6.07	6.00	2.68	0.98	0.34	32.1
**7**	**12**	**12.03**	**12.00**	**2.68**	**2.68**	**0.64**	**0.0**

Supplementary Table 32. UPBE0 Spin ladder for $Co_2(OH)_2$(bbta). Spin ladder for $Co_2(OH)_2$(bbta). Relative energy stability (ΔE, in kJ mol^{-1}), Hirshfeld spin density on Co (ρ_{Co}) and on all the other atoms ($\rho_{no\text{-}metal}$) as obtained at the **UPBE0**/def2-TZVP level for the **Co_2Mg_2OH** cluster for different spin multiplicities (2S + 1). The theoretical value for S^2 ($S(S+1)_{theo}$) along with those obtained in the calculations before ($S(S+1)_{before}$) and after ($S(S+1)_{after}$) the default correction of Gaussian 09 for the higher spin state is also reported.

2*S*+1	$S(S+1)_{theo}$	$S(S+1)_{before}$	$S(S+1)_{after}$	ρ_{Co1}	ρ_{Co2}	$\rho_{no\text{-}metal}$	Δ*E*
1	0	0.00	0.00	0.00	0.00	0.00	325.9
3	2	2.02	2.00	0.98	0.98	0.04	73.7
**5**	**6**	**6.51**	**6.01**	**2.70**	**0.96**	**0.33**	**0.0**
7	12	12.02	12.00	2.70	2.70	0.59	13.1

Supplementary Table 33. **UB3LYP Spin ladder for $Co_2(OH)_2$(bbta). Spin ladder for $Co_2(OH)_2$(bbta).** Relative energy stability (ΔE, in kJ mol^{-1}), Hirshfeld spin density on Co (ρ_{Co}) and on all the other atoms ($\rho_{no\text{-}metal}$) as obtained at the **UB3LYP**/def2-TZVP level for the **Co_2Mg_2OH** cluster for different spin multiplicities (2S + 1). The theoretical value for S^2 ($S(S+1)_{theo}$) along with those obtained in the calculations before ($S(S+1)_{before}$) and after ($S(S+1)_{after}$) the default correction of Gaussian 09 for the higher spin state is also reported.

2*S*+1	$S(S+1)_{theo}$	$S(S+1)_{before}$	$S(S+1)_{after}$	ρ_{Co1}	ρ_{Co2}	$\rho_{no\text{-}metal}$	Δ*E*
1	0	0.00	0.00	0.00	0.00	0.00	245.2
3	2	2.02	2.00	0.97	0.97	0.06	39.2
**5**	**6**	**6.29**	**6.00**	**2.67**	**0.97**	**0.37**	**0.0**
7	12	12.02	12.00	2.66	2.67	0.67	30.1

Supplementary Table 34. Relative energy stability (kJ mol^{-1}) obtained at the unrestricted UM06, UPBE0, and UB3LYP/def2-TZVP level of theory for the $\mathbf{Co_2Mg_2OH}$ cluster for increasing spin multiplicities (2S + 1).

**2*S*+1**	**M06**	**PBE0**	**B3LYP**
**1**	270.3	325.9	245.2
**3**	55.8	73.7	39.2
**5**	32.1	**0.0**	**0.0**
7	**0.0**	13.1	30.1

CASSCF and CASPT2 calculations were performed for Co_2Mg_2OH using a (6,6) (see Supplementary Table 35) and a (6,12) active space (see Supplementary Table 36). The descriptions obtained using each active space are equivalent, indicating the reliability of the results obtained also using a minimal active space. This result is important in view of the calculations for the tetracobalt cluster, where only the minimal active space has an affordable computational cost. Both CASSCF and CASPT2 predict the singlet to be the most stable spin state with all the other states within a few kJ mol^{-1} and an energy that is two orders of magnitude lower than predicted by DFT. The singlet wave function is a linear combination of sixteen different electronic configurations having very close CI contributions.

Supplementary Table 35. CAS(6,6) relative energy stability (kJ mol^{-1}) obtained at the CASSCF and CASPT2 level of theory using a (6,6) active space for the $\mathbf{Co_2Mg_2OH}$ cluster for increasing spin multiplicities (2S + 1).

**2*S*+1**	**CASSCF(6,6)**	**CASPT2**
**1**	**0.0**	**0.0**
3	0.0	1.6
5	0.1	2.7
7	0.1	3.4

Supplementary Table 36. CAS(6,12) relative energy stability (kJ mol^{-1}) obtained at the CASSCF and CASPT2 level of theory using a (6,12) active space for the **Co_2Mg_2OH** cluster for increasing spin multiplicities (2S + 1).

2*S*+1	CASSCF(6,12)	CASPT2
**1**	**0.0**	**0.0**
3	0.0	1.4
5	0.1	2.2
7	0.2	1.4

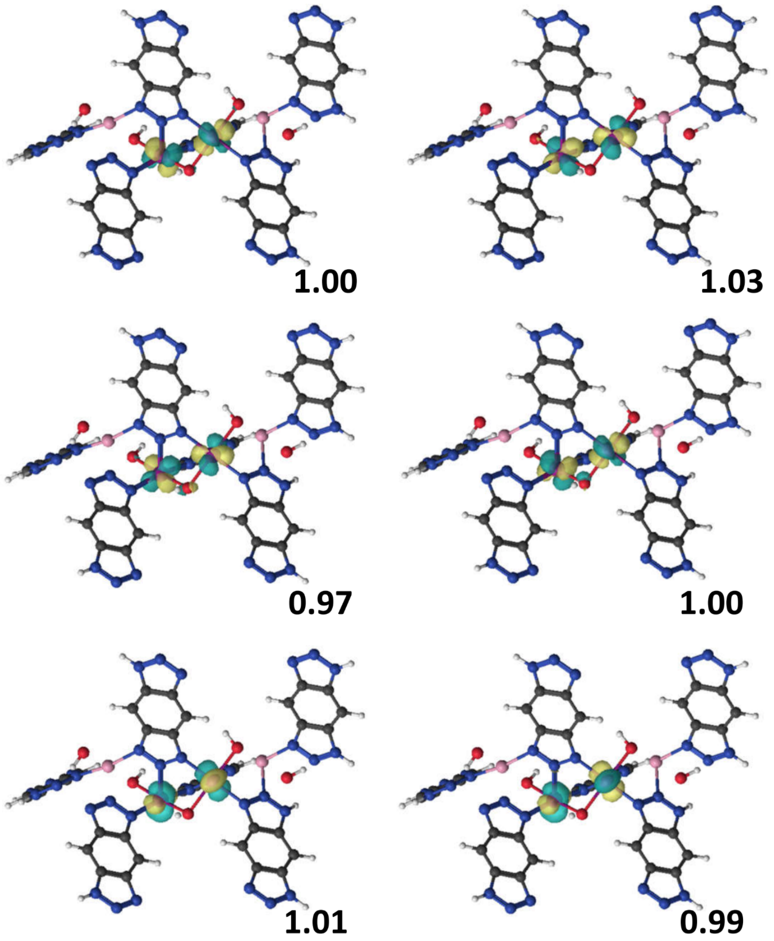

Supplementary Figure 74. Co 3d orbitals in Co_2Mg_2OH computed with the (6,6) active space for the singlet state along with their natural orbital occupation numbers. Color code: Co (purple), C (grey), N (blue), O (red), H (white).

Co_4OH Cluster Model

For the Co_4OH cluster, the singlet spin state is predicted by DFT functionals to be the highest in energy. With UM06, three broke-symmetry solutions were obtained for the singlet state, which are higher than the S = 6 state. The formation of antiferromagnetic Co coupling causes a significant stabilization of the system with respect of the pure singlet state (about 500 kJ mol^{-1}). Nevertheless, broken-symmetry solutions where all cobalt atoms have three unpaired electrons could not be obtained.

Supplementary Table 37. M06 Spin ladder for $Co_2(OH)_2$(bbta). Relative energy stability (ΔE, in kJ mol^{-1}), Hirshfeld spin density on Co (ρ_{Co}) and on all the other atoms ($\rho_{no\text{-}metal}$) as obtained at the **UM06**/def2-TZVP level for the **Co_4OH** cluster at different spin multiplicities (2S + 1). The theoretical value for S^2 ($S(S+1)_{theo}$) along with those obtained in the calculations before ($S(S+1)_{before}$) and after ($S(S+1)_{after}$) the default correction of Gaussian 09 for the higher spin state is also reported.

**2*S*+1**	**$S(S+1)_{theo}$**	**$S(S+1)_{before}$**	**$S(S+1)_{after}$**	**ρ_{Co1}**	**ρ_{Co2}**	**ρ_{Co3}**	**ρ_{Co4}**	**$\rho_{no\text{-}metal}$**	**Δ*E***
1	0	0.00	0.00	0.00	0.00	0.00	0.00	0.00	573.9
3	2	3.23	2.58	1.02	-0.98	0.97	1.07	-0.07	142.2
5	6	6.25	6.02	1.02	0.98	0.97	1.06	-0.03	142.8
7	12	12.16	12.01	1.02	0.98	2.68	1.06	0.25	110.1
9	20	20.25	20.01	2.67	0.98	0.98	2.61	0.76	63.7
11	30	30.16	30.00	2.68	2.68	0.98	2.63	1.04	24.9
**13**	**42**	**42.09**	**42.00**	**2.67**	**2.68**	**2.69**	**2.63**	**1.32**	**0.0**
BS-1-$\alpha^{LS}\beta^{HS}\alpha^{HS}\beta^{LS}$	0	4.09	12.54	1.04	-2.73	2.75	-1.05	0.00	86.8
BS-1-$\beta^{LS}\alpha^{HS}\alpha^{LS}\beta^{HS}$	0	4.17	12.91	-1.02	2.69	0.96	-2.63	-0.01	56.8
BS-1-$\alpha^{HS}\alpha^{LS}\beta^{HS}\beta^{LS}$	0	4.22	13.17	2.72	0.97	-2.75	-1.05	2.83	65.2

Supplementary Table 38. PBE0 Spin ladder for $Co_2(OH)_2$(bbta). Relative energy stability (ΔE, in kJ mol^{-1}), Hirshfeld spin density on Co (ρ_{Co}) and on all the other atoms ($\rho_{no\text{-}metal}$) as obtained at the **UPBE0**/def2-TZVP level for the **Co_4OH** cluster at different spin multiplicities (2S + 1). The theoretical value for S^2 ($S(S+1)_{theo}$) along with those obtained in the calculations before ($S(S+1)_{before}$) and after ($S(S+1)_{after}$) the default correction of Gaussian 09 for the higher spin state is also reported.

**2*S*+1**	**$S(S+1)_{theo}$**	**$S(S+1)_{before}$**	**$S(S+1)_{after}$**	**ρ_{Co1}**	**ρ_{Co2}**	**ρ_{Co3}**	**ρ_{Co4}**	**$\rho_{no\text{-}metal}$**	**Δ*E***
1	0	0.00	0.00	0.00	0.00	0.00	0.00	0.00	708.8
3	2	-	-	-	-	-	-	-	-
5	6	6.08	6.00	1.02	0.98	0.98	1.06	-0.05	203.9
7	12	-	-	-	-	-	-	-	-
9	20	20.05	20.00	1.02	0.99	2.70	2.66	0.63	113.5
11	30	30.04	30.00	2.70	0.98	2.71	2.66	0.94	30.8
**13**	**42**	**42.04**	**42.00**	**2.70**	**2.70**	**2.71**	**2.66**	**1.22**	**0.0**

Supplementary Table 39. B3LYP Spin ladder for $Co_2(OH)_2$(bbta). Relative energy stability (ΔE, in kJ mol^{-1}), Hirshfeld spin density on Co (ρ_{Co}) and on all the other atoms ($\rho_{no\text{-}metal}$) as obtained at the **UB3LYP**/def2-TZVP level for the **Co_4OH** cluster at different spin multiplicities (2S + 1). The theoretical value for S^2 ($S(S+1)_{theo}$) along with those obtained in the calculations before ($S(S+1)_{before}$) and after ($S(S+1)_{after}$) the default correction of Gaussian 09 for the higher spin state is also reported.

**2*S*+1**	***S*(*S*+1)$_{theo}$**	***S*(*S*+1)$_{before}$**	***S*(*S*+1)$_{after}$**	**ρ_{Co1}**	**ρ_{Co2}**	**ρ_{Co3}**	**ρ_{Co4}**	**$\rho_{no\text{-}metal}$**	**Δ*E***
1	0	0.00	0.00	0.00	0.00	0.00	0.00	0.00	515.2
3	2	5.49	12.17	1.02	0.97	2.66	-2.60	-0.06	28.7
5	6	7.79	7.36	1.02	0.97	2.67	-0.99	0.33	59.4
7	12	12.81	12.16	1.02	0.97	2.67	1.05	0.29	58.0
9	20	20.66	20.06	2.66	2.67	0.95	1.04	0.68	30.0
11	30	30.35	30.00	2.66	2.68	2.68	1.05	0.94	0.4
**13**	**42**	**42.05**	**42.00**	**2.66**	**2.67**	**2.68**	**2.61**	**1.38**	**0.0**

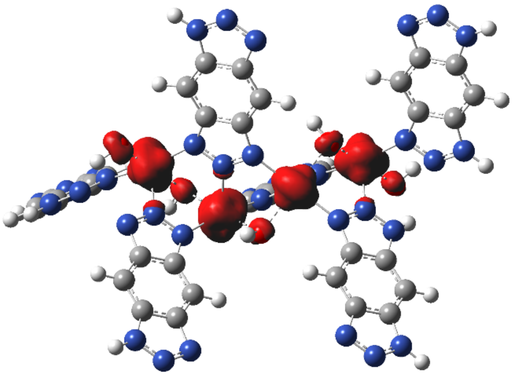

Supplementary Figure 75. Spin density on Co_4OH for S = 6 as optimized at the UM06/def2-TZVP level and freezing the position of Co, O and N to the values determined here from structural studies. Red density correspond to alpha electron excess (isosurface: 0.01 au). Atoms labels: Co (purple), O (red), Mg (pink), N (blue), C (gray), H (white).

Supplementary Table 40. Relative energy stability (kJ mol^{-1}) obtained at the unrestricted UM06, UPBE0, and UB3LYP/def2-TZVP level of theory for the **Co_4OH** cluster at increasing spin multiplicities (2S + 1).

2*S*+1	M06	PBE0	B3LYP
1	573.9	708.8	515.2
3	142.2	-	28.7
5	142.8	203.9	59.4
7	110.1	-	58.0
9	63.7	113.5	30.0
11	24.9	30.8	0.4
**13**	**0.0**	**0.0**	**0.0**
BS-1-$\alpha^{LS}\beta^{HS}\alpha^{HS}\beta^{LS}$	86.8	-	-
BS-1-$\beta^{LS}\alpha^{HS}\alpha^{LS}\beta^{HS}$	56.8	-	-
BS-1-$\alpha^{HS}\alpha^{LS}\beta^{HS}\beta^{LS}$	65.2	-	-

CASPT2 calculations conducted using a (12,12) active space indicated the singlet state as the ground spin state also for **Co_4OH**. All the Co atoms are in the high spin state, in agreement with the magnetic measurements of the activated material (Supplementary Figure 63). All the other spin states of the system (corresponding to high spin Co in a ferromagnetic configuration) are almost isoenergetic, being less than 4.3 kJ mol^{-1} higher in energy.

Supplementary Table 41. CAS(12,12) relative energy stability (kJ mol^{-1}) obtained at the CASSCF and CASPT2 level of theory using a (12,12) active space for the **Co_4OH** cluster for increasing spin multiplicities (2S + 1).

2S+1	CASSCF(12,12)	CASPT2
**1**	0.1	**0.0**
**3**	0.1	1.0
**5**	0.1	1.7
**7**	0.1	2.4
**9**	0.0	2.0
**11**	0.1	3.2
**13**	0.1	4.3

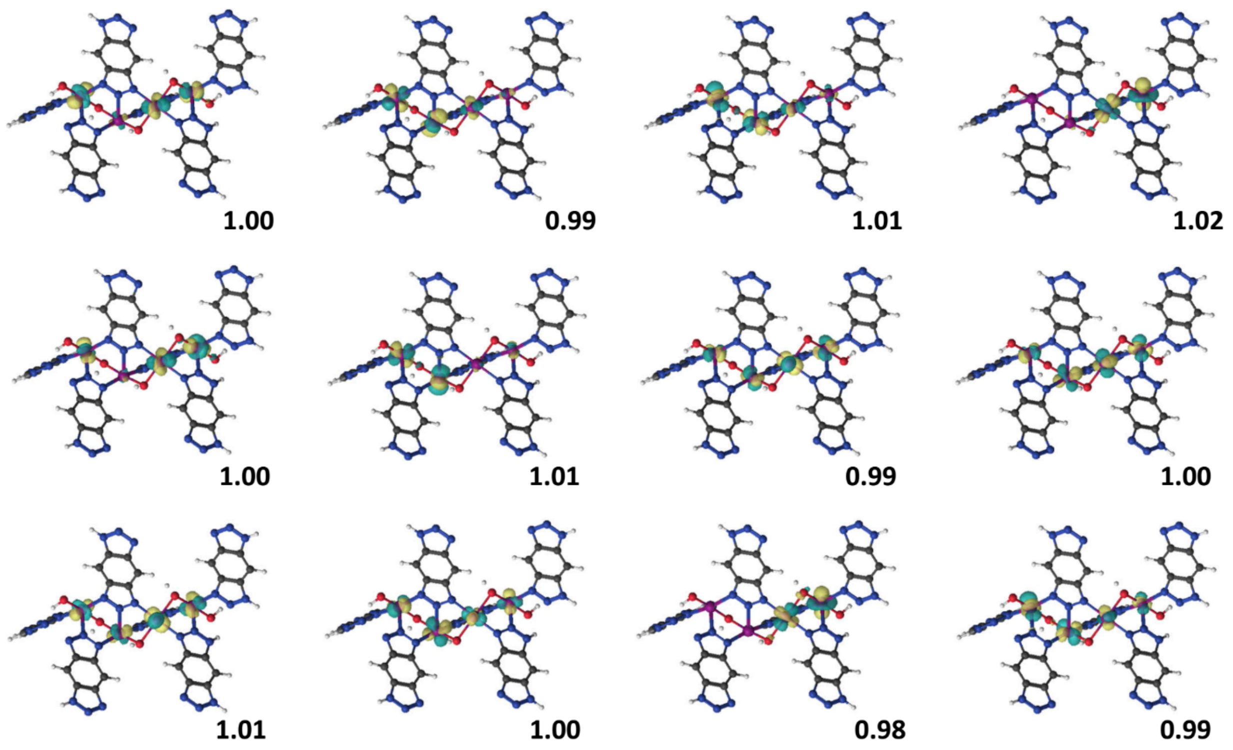

Supplementary Figure 76. Co 3d orbitals in Co_4OH computed with the (12,12) active space for the singlet state along with their natural orbital occupation numbers. Color code: Co (purple), C (grey), N (blue), O (red), H (white).

16. Supplementary Methods: Periodic models for O_2 adsorbed on Co_2Cl_2(bbta) and $Co_2(OH)_2$(bbta)

Isolated MOFs

The structures of the activated MOFs were optimized at the PBE-3D level, considering a nine unit formula cell (18 Co atoms). Two possible electronic configurations were considered: (a) the Co centers in a high spin configuration ("HS," three unpaired α electrons for Co) and (b) all Co centers in a low spin configuration ("LS," one unpaired α electrons per Co). In both cases, the unpaired α electrons were considered to be all ferromagnetically coupling, for a total of 54 unpaired α electrons (n_α) for the HS state and 18 for the LS state. Cluster calculations reported in Supplementary Information Section 14 evidenced that for the same structure, the antiferromagnetic and ferromagnetic configurations are essentially degenerate. The optimized wave function obtained by fixing the total n_α to 0 at this level of theory corresponded to the antiferromagnetic arrangement of LS Co atoms. The structures optimized with $n_\alpha = 0$ were fully equivalent to the $n_\alpha = 18$ ones on the geometrical and energetic point of view, confirming what expected from the cluster calculations and validating the decision to optimize the systems imposing a ferromagnetic arrangement of the unpaired α electrons of the Co atoms. The $n_\alpha = 0$ structures will not be discussed in the following.

Supplementary Figures 77a and 78a show the optimized structures for $Co_2(OH)_2$(bbta)-HS and Co_2Cl_2(bbta)-HS, respectively, while the optimized coordinated for all the structures are reported in Supplementary Information Section 17.

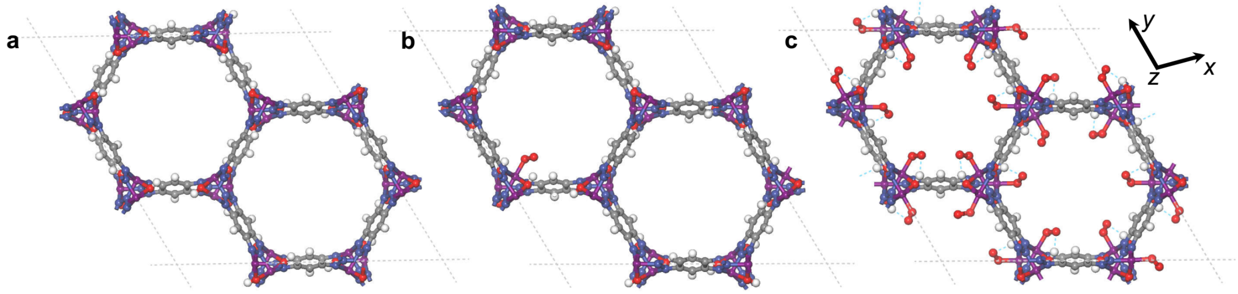

Supplementary Figure 77. $Co_2(OH)_2$(bbta) optimized periodic structures at PBE level (520 eV energy cutoff) for **a**, the activated MOF $Co_2(OH)_2$(bbta)-HS, and considering **b**, 1 O_2 molecule per cell, $Co_2(OH)_2$(bbta)-1O_2-2OH, and **c**, 18 O_2 molecule per cell, $Co_2(OH)_2$(bbta)-18O_2.The C atoms are reported in gray, H atoms in white, O in red, Co in purple and N in blue.

Supplementary Table 42. Lattice parameters and cell volumes for $Co_2(OH)_2$(bbta) and Co_2Cl_2(bbta) as optimized using the PBE-D3 functional considering all cobalt ions in high (HS, $n_\alpha = 54$) or in low spin configurations (LS, $n_\alpha = 18$). Experimental values ("Expt") are also reported for comparisons. The relative stability of the two spin configurations (ΔE) as obtained at the PBE-D3 level and by single point calculations with PBE0-D3 are also reported. Axial distances are in Å, volumes in Å^3, and energies in kJ mol^{-1} mol$_{Co}^{-1}$.

	**$Co_2(OH)_2$(bbta)**			**Co_2Cl_2(bbta)**		
	**Expt**	**HS**	**LS**	**Expt**	**HS**	**LS**
A	25.31	25.29	24.74	24.92	24.85	24.33
B	25.31	25.31	24.96	24.92	24.86	24.32
C	7.99	7.90	7.66	8.15	8.04	7.73
V	4427	4363	4028	4386	4289	3976
$\Delta E_{PBE\text{-}D3}$	-	0.0	-32.5	-	0.0	-23.8
$\Delta E_{PBE0\text{-}D3}$	-	0.0	27.6	-	0.0	35.6

The HS and LS structures are remarkably different. In fact, a unit cell contraction of about 10% is observed going from HS to LS (compare *V* in the HS and LS columns in Suppl3mentary Table 42) due mainly to the shrinking of Co-N bonds (see Supplementary Figure 79). Moreover, a large cell contraction is expected upon adsorption of any adsorbate able to cause a high to low spin transition of the Co centers. SQUID measurements and multireference wave function based calculations indicate that the Co atoms are in HS in their ground state (see Supplementary Information Section 14). In addition, the calculated lattice parameters for the HS state are closer than the LS ones to the experimentally derived lattice parameters. In addition, the coordination environment around the cobalt centers are very similar between both the calculated HS state and the experimentally derived structure of $Co_2(OH)_2$(bbta).

Although able to correctly model the MOF structure, PBE-D3 is not able to describe the relative stability of the HS and LS spin states. In fact, the LS state is predicted to be more stable than the HS state, for both $Co_2(OH)_2$(bbta) and Co_2Cl_2(bbta) (see Supplementary Table 42). This is a common problem in DFT methods, in particular local functionals, that requires assessment using experimental results and multireference results as benchmarks.[12–14] In Supplementary Information Section 14, it was shown that the three hybrid DFT functionals considered (M06, PBE0 and B3LYP) are able to correctly predict the ground spin state of the Co atoms in these MOFs. Single point calculations using PBE0-D3 were then performed on the PBE-D3 optimized geometries, confirming the HS structures as the most stable (see $\Delta E_{\mathrm{PBE0\text{-}D3}}$ values in Supplementary Table 42).

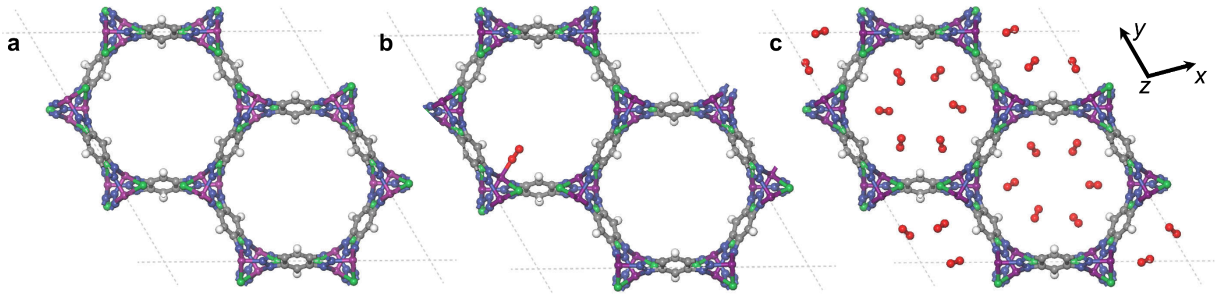

Supplementary Figure 78. Co_2Cl_2(bbta) optimized structures at PBE level (520 eV energy cutoff) for **a**, the activated MOF Co_2Cl_2(bbta) ($n_\alpha = 54$), and the MOF interacting with **b**, 1 O_2 molecule Co_2Cl_2(bbta)-1O_2 ($n_\alpha = 56$), and **c**, 18 O_2 molecules Co_2Cl_2(bbta)-18O_2 ($n_\alpha = 90$). The C atoms are reported in gray, H atoms in white, O in red, Co in purple, Cl in green, and N in blue.

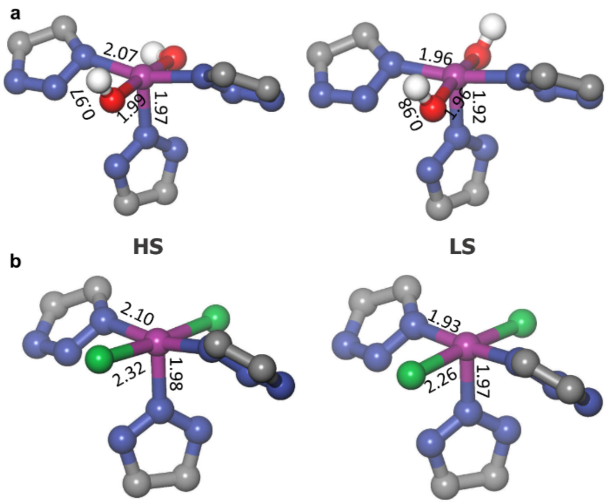

Supplementary Figure 79. **Co geometry in the isolated MOFs.** First coordination sphere of Co in the **a**, (from left to right) $Co_2(OH)_2$(bbta)-HS, $Co_2(OH)_2$(bbta)-LS and **b**, (from left to right) Co_2Cl_2(bbta)-HS, Co_2Cl_2(bbta)-LS as obtained by optimization of the periodic models at the PBE-D3 level. The C atoms are reported in gray, H atoms in white, O in red, Cl in green, Co in purple, and N in blue. All the bond distances are in Å.

The computed structure for $Co_2(OH)_2$(bbta) allows us to locate the hydrogen atoms of the hydroxo groups, information that is missing from the experimental powder X-ray diffraction structures. This is valuable information because of the likely involvement of these groups in adsorption processes driven by the Co centers. Interestingly, the hydroxo groups in the material appear to be interconnected via weak hydrogen bonds with hydroxo groups in adjacent chains, situated at 4.1 Å (estimated to be 2 kJ mol^{-1} at the PBE-D3 level). However, the distance between the two hydroxo groups coordinated to the same Co atom (4.5 Å) is large enough to vanish any interaction between them. The *trans* and the *cis* are isoenergetic, differing by less than 1 kJ mol^{-1}.

O_2 adsorption on Co_2Cl_2(bbta)

Two different n_α were used in the calculations for the model considering the adsorption of the first O_2 molecule in Co_2Cl_2(bbta): 52 and 56. $n_\alpha = 52$ (1 n_α for the Co···O_2 complex) could correspond to two possible phenomena: (i) the oxidation of the Co(II) to Co(III) with the formation of superoxo species or (ii) the transition of the Co(II) from HS to LS. $n_\alpha = 56$ (5 n_α for the Co···O_2 complex) corresponds to the ferromagnetic coupling between the unpaired electrons on O_2 and those on Co. The structure optimized fixing n_α to 52 is reported in Supplementary Figure 80a. In this case, O_2 is coordinated to Co with a Co–O_2 distance of 1.9 Å, and the O_2 bond length is lengthened by 0.06 Å (Supplementary Figure 80b), suggesting the formation of a strong bond between the Co and O_2. This result, in evident contradiction with the experimental structural data, is due to the over stabilization of the LS state with respect to the HS state by PBE-D3. Single point calculations at PBE0-D3 provide a positive adsorption energy (ΔE_{ads} = 39 kJ mol^{-1}). The model optimized for n_α = 56 is slightly lower in energy and it is reported in Supplementary Figure 78b while the magnified view on the local coordination environment of the Co center is reported in Supplementary Figure 81a. In this case as well PBE0 predicts the process to be endothermic, while PBE is providing reasonable ΔE_{ads}. The experimental isosteric heat for O_2 on this material is only approximately 9 kJ mol^{-1} (see Supplementary Tables 3–4). A systematic underestimation of the adsorption energy by PBE0-D3 of about 20 kJ mol^{-1} is observed for all the systems here studied. However, even with this underestimation, the calculated PBE0 ΔE_{ads} is positive. This can be attributed to the fact that the PBE0 calculations are performed at the PBE geometries. The geometry of the Co-O_2 dimer calculated using PBE-D3 (Supplementary Figure 81b) is significantly different from that obtained for the corresponding cluster model (see Supplementary Figure 86, Supplementary Information Section 16) using a functional that correctly reproduce the spin ladder (M06). This is the only case where such a discrepancy among the periodic and the cluster geometries is found.

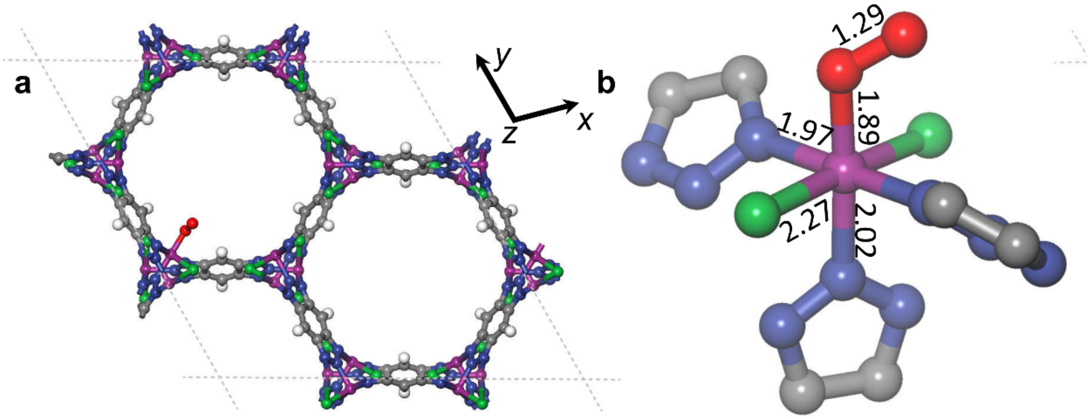

Supplementary Figure 80. Co_2Cl_2(bbta)-$1O_2$ optimized structure at PBE-D3 level (520 eV energy cutoff, $n_\alpha = 52$). **a**, Crystallographic cell. **b**, First coordination sphere of Co. The C atoms are reported in gray, H atoms in white, O in red, Cl in green, Co in purple and N in blue.

The structure optimized considering the Co:O_2 = 1 coverage is reported in Supplementary Figure 78c. It was obtained by considering $n_\alpha = 90$, on the basis of the results obtained for the adsorption of one O_2 molecule. In this case, all the O_2 molecules are localized in the central part of the channel in agreement with the experiment and with cluster calculations. Accordingly, PBE-D3 and PBE0-D3 adsorption energy are very close and indicate a very low interaction between the MOF and the O_2 molecules.

Supplementary Table 43. Electronic, geometrical and energetic parameters for O_2 adsorption in the periodic models of $Co_2(OH)_2$(bbta) and Co_2Cl_2(bbta) as calculated by single point PBE0-D3 calculations on the optimized PBE-D3 structures (n_α, number of unpaired α electrons per cell). V: cell volume (in Å^3), ρ(Co) and $\rho(O_2)$: spin density on Co and O_2 defined as the difference between the number of α and β electrons. $d(\text{Co-O}_{O2})$: distance between Co and the closest oxygen of the adsorbate (in Å), Δd(O-O): variation in the O_2 bond length (in Å) with respect to the gas phase (1.23 Å), $\Delta E_{\text{ads-PBE}}$ and ΔE_{ads}: average O_2 adsorption energy as calculated using the PBE and the PBE0 functional, respectively (in kJ mol^{-1}).

	n_α	V	ρ(Co)	$\rho(O_2)$	$d(\text{Co-O}_{O2})$	Δd(O-O)	$\overline{\Delta E_{\text{ads-PBE}}}$	$\overline{\Delta E_{\text{ads}}}$
$Co_2(OH)_2$(bbta)								
$0O_2$	54	4363	2.67					
$1O_2$-0OH	52	4322	−0.23	1.05	1.88	0.06	−136.4	1.9
$1O_2$-1OH	52	4326	−0.07	0.90	1.87	0.08	−159.3	−20.2
$1O_2$-2OH	52	4321	−0.05	0.89	1.86	0.08	−166.1	−27.8
$1O_2$-2OH-chiral	52	4323	−0.06	0.89	1.86	0.08	−160.6	−27.6
$2O_2$-dfdp	50	4280	−0.05/−0.05	0.89/0.89	1.86/1.86	0.08/0.08	−170.9	−33.0
$2O_2$-dfsp-a	50	4285	−0.06/−0.06	0.90/0.90	1.86/1.86	0.08/0.08	−166.6	−28.6
$2O_2$-dfsp-b	50	4284	−0.06/−0.06	0.89/0.89	1.86/1.86	0.08/0.08	−167.9	−28.3
$2O_2$-sfdp	50	4289	−0.05/−0.06	0.88/0.90	1.86/1.87	0.08/0.08	−171.1	−23.8
$1O_2$-2OH-$1O_2$-ontop	50/54	4317	−0.05/2.66	0.88/1.68	1.86/5.19	0.08/0.0	−87.6	−14.2
$18O_2$	18	3903	0.07	0.79	1.87	0.07	−161.9	−22.7
Co_2Cl_2(bbta)								
$0O_2$	54	4289	2.65					
$1O_2$	52	4262	−0.45	1.21	1.89	0.06	−106.8	38.7
$1O_2$	56	4294	2.74	1.69	1.98	0.04	−22.0	32.8
$18O_2$	90	4242	2.65	1.69	3.14	0.00	−13.2	−5.5

O_2 adsorption on $Co_2(OH)_2$(bbta)

Adsorption of O_2 on $Co_2(OH)_2$(bbta) is expected to be a more complex interaction than adsorption in $Co_2(Cl)_2$(bbta) because of the presence of the hydroxo groups in the first coordination shell of the Co centers. Three different geometries have been considered for the adsorption of 1 O_2 per cell on $Co_2(OH)_2$(bbta), differing in the number of hydroxo groups interacting with O_2: 0 ($Co_2(OH)_2$(bbta)-$1O_2$-0OH), 1 ($Co_2(OH)_2$(bbta)-$1O_2$-1OH) and 2 ($Co_2(OH)_2$(bbta)-$1O_2$-2OH). The corresponding Co sites are labeled as Co-0OH, Co-1OH and Co-2OH, respectively. These structures have been optimized by fixing n_α = 52. In all the three cases, we obtained a structure in good agreement with the experimental structure (see Supplementary Table 44 and Supplementary Figure 81b-d) as well as the structure optimized at the M06 level in cluster calculations (see Supplementary Information Section 16). The spin densities on the Co and O_2 obtained both at PBE-D3 and PBE0-D3 levels indicate that in all cases Co(II) is oxidized to Co(III) and O_2 is reduced to a superoxo species (O_2^-). $Co_2(OH)_2$(bbta)-$1O_2$-0OH, -1OH, and -2OH geometries,

although very similar, have different ΔE_{ads}. This is likely due to a significant increase in the complex stability upon increasing the number of hydroxo groups involved in the interaction with O_2. The largest stability gain is observed upon introducing one hydroxo group interaction—i.e., upon going from Co-0OH to Co-1OH. This initial –OH group engages in a short hydrogen bond with O_2 (~1.9 Å), resulting in a lowering of the adsorption energy by ~22 kJ mol^{-1} relative to the structure with no hydroxo group interaction (22.9 and 22.1 kJ mol^{-1} using PBE-D3 and PBE0-D3, respectively; see Supplementary Table 43). The intervention of the second –OH would cause a further decrease in the adsorption energy of ~7 kJ mol^{-1} (6.8 and 7.6 kJ mol^{-1} using PBE-D3 and PBE0-D3, respectively; see Supplementary Figure 83f) due to the electrostatic interaction between the positively charged hydrogen H atom of the –OH group and the negative lobes of the adsorbed O_2 electrostatic potential. In contrast, ΔE_{ads} calculated for Co-0OH is lower than that obtained for Co_2Cl_2(bbta) by ~30 kJ/mol (29.6 and 36.8 kJ/mol for PBE-D3 and PBE0-D3, respectively). As a result, the larger affinity for O_2 observed experimentally and computationally for $Co_2(OH)_2$(bbta) in comparison to Co_2Cl_2(bbta) can be attributed in part to the formation of the hydrogen bond to adsorbed O_2, in addition to the differences in coordination environment between the two materials.

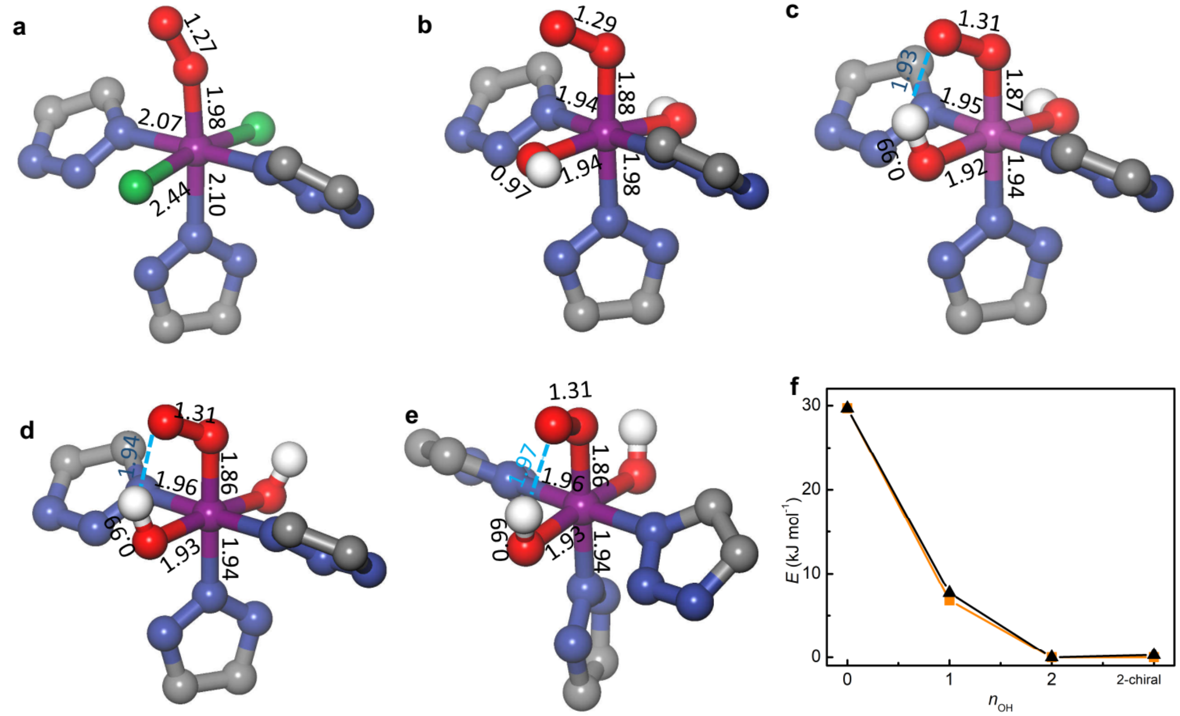

Supplementary Figure 81. First coordination sphere of Co in **a**, Co_2Cl_2(bbta)-1O_2 ($n_\alpha = 56$), **b**, $Co_2(OH)_2$(bbta)-1O_2-0OH **c**, $Co_2(OH)_2$(bbta)-1O_2-1OH, **d**, $Co_2(OH)_2$(bbta)-1O_2-2OH, **e**, $Co_2(OH)_2$(bbta)-1O_2-2OH-chiral as obtained by optimization of the periodic models at the PBE-D3 level. The C atoms are reported in gray, H atoms in white, O in red, Cl in dark green, N in blue and Co in purple. All the bond distances are in Å. **f**, Relative energy of the $Co_2(OH)_2$(bbta)-1O_2 complex as function of the number of hydroxo groups contributing to the interaction (orange squares: PBE-D3; black triangles: PBE0-D3).

Experimental IR spectra show a significant perturbation of the bands associated to the –OH stretching and bending after O_2 adsorption. In particular the formation of a broad signal at about 3400 cm^{-1} associated to the formation of a H-bond is observed, supporting the supporting the cluster calculations reported in Supplementary Information Section 16. An additional model

considering O_2 adsorption on the chiral equivalent of the Co site in Supplementary Figure 83b ($Co_2(OH)_2$(bbta)-1O_2-2OH-chiral, Supplementary Figure 83d), evidenced that there is no effect of the chirality of the Co centers on the O_2 complex properties, as expected.

Supplementary Table 44. Relevant geometrical parameters of $Co_2(OH)_2$(bbta) as calculated with PBE-D3 for the interaction with 1 O_2 molecule (1O_2-2OH) compared with the corresponding experimental values. Distances in Å, angle in degrees.

	**Expt.**	**PBE-D3**
d(O-O)	1.311	1.31
d(Co-O_{O2})	1.901	1.86
d(Co-O_{OH})	1.952	1.93
d(Co-N1)	1.920	1.95
d(Co-N2)	1.994	1.96
∠Co-O-O	120.8	116

Another model was considered having a Co:O_2 = 1 ($Co_2(OH)_2$(bbta)-18O_2, $n_\alpha = 18$). The optimized structure is reported in Supplementary Figure 77c, while relevant electronic, geometrical and energetic parameters are reported in Supplementary Table 43. The calculated cell volume was compared with the experimental value extrapolated from the plot in Supplementary Figure 83. Good agreement was obtained between this extrapolated value and the computed volume (3919 and 3903 Å^3, respectively). Gas adsorption in MOFs having linkers with similar dimensions of the bbta^{2-} linker typically undergo contraction of only a few percent of the volume under similar gas loadings,[29] so the contraction predicted here of approximately 10.5% is quite unusual. By comparison with the values in Supplementary Table 42, the large reduction of $Co_2(OH)_2$(bbta) volume upon O_2 adsorption can be mainly associated with the oxidation of the Co centers, causing a shortening of the Co–N bonds that is equally distributed along the three Cartesian axes. The average adsorption energy calculated for the $Co_2(OH)_2$(bbta)-18O_2 situation is less exothermic than that calculated for the $Co_2(OH)_2$(bbta)-1O_2-2OH model and is close to that calculated for $Co_2(OH)_2$(bbta)-1O_2-1OH (−22.7 kJ mol^{-1} vs. −20.2 kJ mol^{-1} for $Co_2(OH)_2$(bbta)-1O_2-1OH using PBE0-D3). In this calculation, the most stable structure is one in which all 18 O_2 molecules interact with a Co-1OH site.

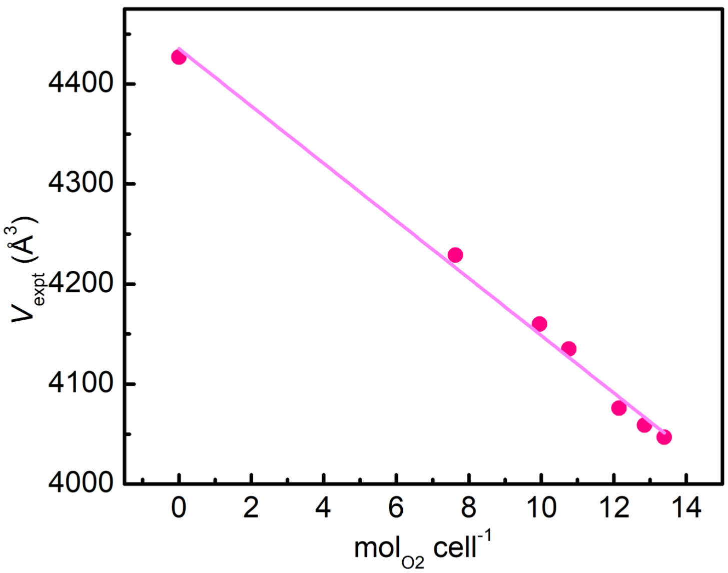

Supplementary Figure 82. Dependence of the experimental cell volume of $Co_2(OH)_2$(bbta) as function of the O_2 coverage over the metal centers (circles). The straight line resulting from the linear fitting of the data is also reported ($R^2 = 0.99$, V [Å^3] = 28.68 · mol$_{O2}$ cell^{-1} +4435.34).

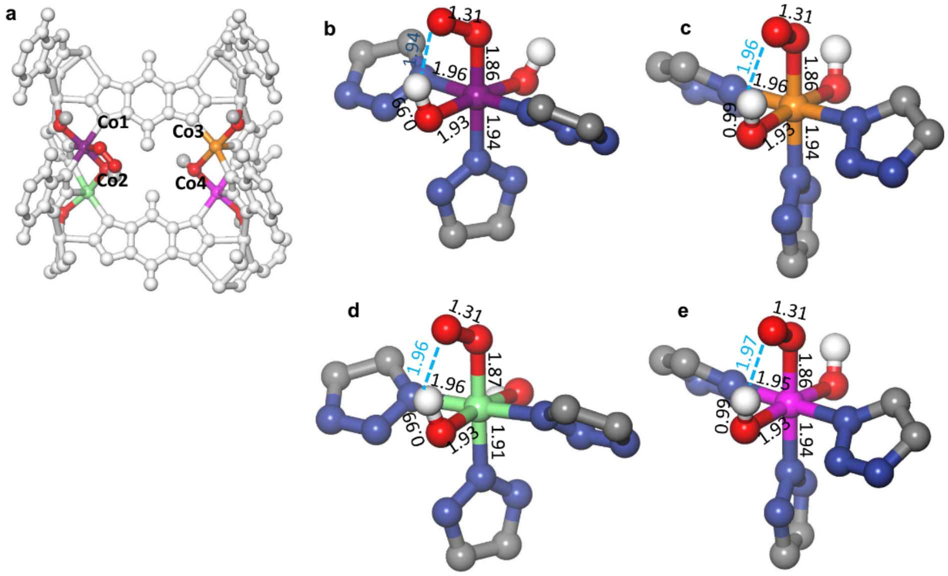

Supplementary Figure 83. Two oxygen adsorbed on $Co_2(OH)_2$(bbta). a, Position of Co1, Co2, Co3 and Co4. First coordination sphere of Co for the first adsorbed molecule on **b**, Co1, and the second oxygen molecule adsorbed on **c**, Co2, **c**, Co3, or **b**, Co4, as obtained by optimization of the periodic models at the PBE-D3 level. The bond length of O_2 in the gas phase is computed to be 1.23 Å. The C atoms are reported in gray, H atoms in white, O in red, Cl in dark green, N in blue and Co in the colors indicated in **a**. All the bond distances are in Å.

We also considered the adsorption of two O_2 molecules in the cell. While for the first molecule all the sites are the same, for the second molecule the Co sites close to that coordinating the first O_2 molecule (Co1) could be different. These sites are indicated in Supplementary Figure 83a as Co2,

Co3, and Co4. The corresponding optimized cells for the $Co_2(OH)_2$(bbta)-2O_2 complexes are reported in Supplementary Figure 84 whereas the enlarged view on the local environment of the Co sites is reported in Supplementary Figure 83b-d. ΔE_{ads} is similar for the adsorption on Co3 and Co4 (see Supplementary Figure 83a), and it amounts to that calculated for $Co_2(OH)_2$(bbta)-1O_2-2OH. For Co2, ΔE_{ads} is the same as that obtained for $Co_2(OH)_2$(bbta)-1O_2-1OH, because of the availability of only 1OH on Co2. This means that the adsorption of one O_2 molecule by a Co site hinders the adsorption on the closest Co sites on the same chain. The formation of $Co_2(OH)_2$(bbta)-1O_2-2OH complexes thus creates an heterogeneity that was not existing in the activated material. This induced heterogeneity could in part explain the observed evolution of the differential enthalpy of adsorption with the coverage. However, it can also have other origins, in particular changes in the reducing power of the cobalt ions upon initial oxidation.

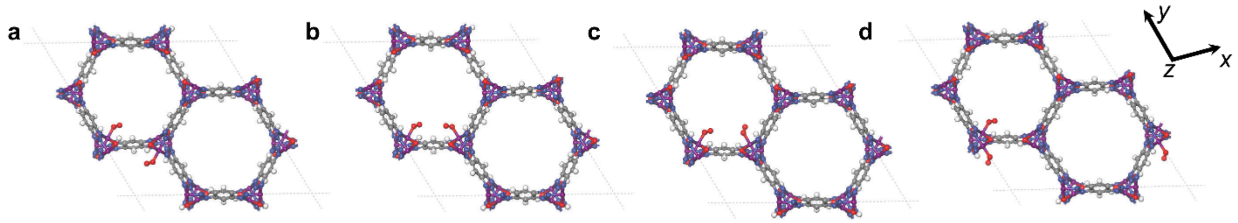

Supplementary Figure 84. $Co_2(OH)_2$(bbta)-2O_2 optimized structures at PBE level (520 eV energy cutoff) considering one O_2 molecule adsorbed on Co1 and the second molecule on **a**, Co4: $Co_2(OH)_2$(bbta)-2O_2-dfdp, **b**, Co3: $Co_2(OH)_2$(bbta)-2O_2-dfsp-a, **c**, Co3: $Co_2(OH)_2$(bbta)-2O_2-dfsp-b, and **d**, Co2: $Co_2(OH)_2$(bbta)-2O_2-sfdp complex. The C atoms are reported in gray, H atoms in white, O in red, Co in purple, and N in blue.

An additional model was considered for the 2 molecules/cell coverage where the second molecule is not coordinated to a Co center but it is adsorbed on the first one (1O_2-2OH-1O_2-ontop). In this case, the solution for n_α = 50 and 54 is isoenergetic and the adsorption energy for the second molecule is of −0.5 kJ mol^{-1}, isoenergetic to the 1O_2-0OH model.

17. Supplementary Methods: Co_4OH and Co_4Cl cluster models for O_2 adsorbed on Co_2Cl_2(bbta) and $Co_2(OH)_2$(bbta)

Vibrational properties of the MOFs at the lowest loading of O_2 were evaluated by using a new set of Co_4OH and Co_4Cl cluster models obtained from the calculated periodic structures reported in Supplementary Information Section 15. The position of the O_2 molecules, cobalt atoms and of the atoms involved in the interaction with O_2 were relaxed at UM06 level, while the other atoms were kept fixed in order to simulate the rigidity of the framework.

O_2 adsorption on Co_2Cl_2(bbta)

The optimized structure for the activated Co_2Cl_2(bbta) (**Co_4Cl** cluster) is reported in Supplementary Figure 85a, while that obtained for Co_2Cl_2(bbta)-1O_2 (**Co_4Cl-1O_2**) is reported in Supplementary Figure 86b. For Co_2Cl_2(bbta)-1O_2 two different models were obtained, carved from the structures optimized for n_α = 52 and n_α = 56. The cluster obtained from the n_α = 56 one was significantly lower in energy than that obtained for n_α = 52 and for this reason it is not discussed in the following text.

Supplementary Table 45. UM06 Spin ladder for Co_4Cl. Relative energy stability (ΔE, in kJ mol^{-1}) as obtained at the **UM06**/def2-TZVP level for the **Co_4Cl** cluster at different spin multiplicities (2S + 1). The theoretical value for S^2 ($S(S+1)_{theo}$) along with those obtained in the calculations before ($S(S+1)_{before}$) and after ($S(S+1)_{after}$) the default correction of Gaussian 09 for the higher spin state is also reported.

**2*S*+1**	***S*(*S*+1)$_{theo}$**	***S*(*S*+1)$_{before}$**	***S*(*S*+1)$_{after}$**	**Δ*E***
1	0	2.26	6.38	165.7
3	2	3.85	4.30	209.4
5	6	6.26	6.02	166.8
7	12	12.23	12.01	123.8
9	20	20.16	20.00	74.5
11	30	30.18	30.00	33.5
**13**	**42**	**42.09**	**42.00**	**0.0**

The ground spin state for **Co_4Cl** is confirmed to be the sextet (see Supplementary Table 45). After the coordination of $1O_2$, the Co atom prefers to change from a high to a low spin configuration (see Supplementary Tables 46 and 47). This transition has been also verified for the **Co_4Cl-$1O_2$** model obtained from the periodic structure optimized for n_α = 52.

Supplementary Table 46. UM06 Spin ladder for Co_4Cl-$1O_2$. Relative energy stability (ΔE, in kJ mol^{-1}), Hirshfeld spin density on Co (ρ_{Co}) and on all the other atoms ($\rho_{no\text{-}metal}$) as obtained at the **UM06**/def2-TZVP level for the **Co_4Cl** cluster at different spin multiplicities (2S + 1). The theoretical value for S^2 ($S(S+1)_{theo}$) along with those obtained in the calculations before ($S(S+1)_{before}$) and after ($S(S+1)_{after}$) the default correction of Gaussian 09 for the higher spin state is also reported.

**2*S*+1**	***S*(*S*+1)$_{theo}$**	***S*(*S*+1)$_{before}$**	***S*(*S*+1)$_{after}$**	**Δ*E***
1	0	3.8	12.0	239.1
3	2	4.6	7.6	169.9
5	6	6.4	6.0	113.4
7	12	12.4	12.0	71.4
9	20	20.8	20.1	171.7
**11**	**30**	**30.3**	**30.0**	**0.0**

The local environment of Co description obtained at the M06 level for the clusters is very close to that calculated using PBE-D3 for the periodic models. PBE-D3 predicts a larger perturbation of O_2, as expected because of the overestimation of the binding energy by this method. Also for the calculation of adsorption energy (ΔE_{ads}^{c} in Supplementary Table 47), the values obtained at the two different levels of calculations are very close and indicates that the adsorption of O_2 in the Co_2Cl_2(bbta) MOF will not be favored. For the clusters, we have evaluated the strain energy contribution, defined as the difference between the energy of the Co_4Cl cluster before and after the adsorption of O_2. The strain-corrected adsorption energy ($\Delta E^{c}_{strain\text{-}free}$) is about 45 kJ mol^{-1} lower than ΔE^{c}, where 70% of the difference is associated to the energy required for the transition of Co from the high to the low spin state, evaluated from the data in Supplementary Table 45.

Supplementary Table 47. Electronic, geometrical and energetic parameters for O_2 adsorption in the Co_4OH and Co_4Cl cluster models as optimized with UM06. ρ(Co) and $\rho(O_2)$: spin density on Co and O_2 defined as the difference between the number of α and β electrons. $d(Co–O_{O2})$: distance between Co and the closest oxygen of the adsorbate (in Å), Δd(O–O): variation in the O_2 bond length (in Å) with respect to the gas phase (1.23 Å), ΔE_{ads}: average O_2 adsorption energy (in kJ mol^{-1}), $\Delta E^{c}_{strain\text{-}free}$ strain-free average O_2 adsorption energy, $\Delta\tilde{\nu}_{O2}$ and $\Delta\tilde{\nu}_{OH}$, shift in the O_2 and in the O-H stretching frequency with respect to the unperturbed value (in cm^{-1}).

	2S+1	ρ(Co)^{b}	$\rho(O_2)$	$d(Co–O_{O2})$	Δd(O–O)	$\Delta E_{ads}{}^{c}$ (ΔE_{ads})	$\Delta E^{c}_{strain\text{-}free}$	$\Delta\tilde{\nu}_{O2}$	$\Delta\tilde{\nu}_{OH}$
Co_4OH									
$0O_2$	13	2.68							
$1O_2$-0OH	11	−0.50	1.50	1.988	0.039	35.1 (29.9)	−54.1	−344	−20/−32
$1O_2$-1OH	11	−0.17	1.17	1.893	0.071	−2.0 (−7.5)	−56.5	−417/-421	−20/−130
$1O_2$-2OH	11	−0.11	1.12	1.884	0.076	2.1 (−3.6)	−68.4	−406/-414	−21/−38/−125
$2O_2$-sfdp	9	−0.16/−0.11^{c}	1.11/1.15^{c}	1.887/1.883^{c}	0.073/0.077^{c}	−7.0 (−12.7)	−68.6	−405/−411/−425	−43/−119/−141
$1O_2$-2OH-1-ontop	11	−0.12/0.98^{c}	1.11/1.99^{c}	1.882/5.018^{c}	0.000/0.077^{c}	11.9 (8.5)	−31.8	+1^{a}/−410/−417	−37/−38/−135
Co_4Cl									
$0O_2$	13	2.64							
$1O_2$	11	−0.79	1.79	2.21	0.012	22.0 (18.0)	−25.8	−145	-

[a]IR inactive mode. [b]We report only the Co spin densities differing from the value in the $0O_2$ cluster. [c]For the systems where two O_2 molecules are present, two values are reported, one for each O_2 molecule.

O_2 adsorption on $Co_2(OH)_2$(bbta)

The optimized structure for the activated $Co_2(OH)_2$(bbta) (**Co_4OH** cluster) is reported in Supplementary Figure 85b. For the interaction with one oxygen molecule (**Co_4OH-$1O_2$**), three different clusters have been considered with an increasing number of –OH interacting with O_2: 0, 1 and 2, in analogy to the periodic case. The optimized structures are reported in Supplementary Figure 86b-d. The adsorption of two O_2 molecules on two vicinal Co sites has been considered with the **Co_4OH-$2O_2$-sfdp** model (Supplementary Figure 86e). The effect of the interaction of a molecule in the gas phase with a coordinated O_2 in the **Co_4OH-$1O_2$-2OH** on its vibrational frequency (a situation possible at higher pressures) has been accounted with the **Co_4OH-$1O_2$-2OH-1-on** cluster.

Supplementary Table 48. UM06 Spin ladder for Co_4OH. Relative energy stability (ΔE, in kJ mol^{-1}) as obtained at the **UM06**/def2-TZVP level for the **Co_4OH** cluster at different spin multiplicities (2S + 1). The theoretical value for S^2 ($S(S+1)_{theo}$) along with those obtained in the calculations before ($S(S+1)_{before}$) and after ($S(S+1)_{after}$) the default correction of Gaussian 09 for the higher spin state is also reported.

**2*S*+1**	**$S(S+1)_{theo}$**	**$S(S+1)_{before}$**	**$S(S+1)_{after}$**	**Δ*E***
1	0	2.08	4.86	147.6
3	2	3.08	2.17	148.0
5	6	7.08	6.10	103.1
7	12	12.08	12.00	103.0
9	20	20.09	20.00	58.9
11	30	30.11	30.00	31.8
13	**42**	**42.11**	**42.00**	**0.0**

Supplementary Table 49. UM06 Spin ladder for Co_4OH-1O_2-2OH. Relative energy stability (ΔE, in kJ mol^{-1}) as obtained at the **UM06**/def2-TZVP level for the **Co_4OH-1O_2-2OH** cluster at different spin multiplicities (2S+1). The theoretical value for S^2 ($S(S+1)_{theo}$) along with those obtained in the calculations before ($S(S+1)_{before}$) and after ($S(S+1)_{after}$) the default correction of Gaussian 09 for the higher spin state is also reported.

**2*S*+1**	**$S(S+1)_{theo}$**	**$S(S+1)_{before}$**	**$S(S+1)_{after}$**	**Δ*E***
1	0	2.09	4.97	111.6
3	2	4.31	5.91	186.5
5	6	7.11	6.13	77.6
7	12	12.11	12.00	77.6
9	20	20.12	20.00	37.8
11	**30**	**30.13**	**30.00**	**0.0**

The geometries optimized in the cluster models are close to that obtained in the periodic structures, while the binding energies are underestimated with respect to the periodic values of about 20-25 kJ mol^{-1}. This difference could be accounted for by the dispersive interaction of the pore with O_2, a contribution that is missing in the cluster models. As in the periodic models, in all the cases, the Co···O_2 complex is described as a redox couple where Co(II) is oxidized to Co(III) and O_2 has been reduced to a superoxo species (O_2^-) (see spin density values reported in Supplementary Table 47). Cluster calculations confirm the sheer increase in the binding energy upon the formation of the hydrogen bond between the –OH group and the O_2 molecule. The O–O bond length is significantly lengthened after adsorption, with Δ*d*(O–O) increasing with the number of interacting –OH species. Accordingly, the calculations suggest that the formation of complexes with or without H-bonding can be detected experimentally by looking at the O_2 stretching region of the IR spectra, since the shift of the O_2 stretching frequency $\Delta\tilde{\nu}_{O2}$ is significantly different for a Co-0OH and for a Co-1OH/2OH site (see Supplementary Table 47). $\Delta\tilde{\nu}_{O2}$ is computed to increase of about 70 cm^{-1} after the formation of the hydrogen bond. In Supplementary Table 47, two values for $\Delta\tilde{\nu}_{O2}$ are reported for the complexes with the Co-2OH and Co-1OH sites. In these cases, the $\tilde{\nu}_{O2}$ mode is always combined with the N-N stretching of the triazolate rings. The change with the pressure of this mode in terms of intensity and band width is expected to be a complex result of the

combination of the changes in the O–O modes with those associated to the changes in the combined framework mode.

The formation of the complex is expected to cause a significant change in several framework modes, especially those involving the hydroxo species. In Supplementary Figure 87 the –OH δ bending modes are reported before and after O_2 coordination: these modes shift about 200 cm^{-1} after O_2 coordination. A band is observed in the experimental spectra for $^{16}O_2$ at about 2000 cm^{-1} that can be assigned to a combination mode between the O_2 stretching frequency and the perturbed δ_{OH} mode. An even larger change is expected in the OH stretching frequency region ($\tilde{\nu}_{OH}$). Interestingly, the value of $\Delta\tilde{\nu}_{OH}$ and the number of bands predicted to form after formation of the complex depends on the number of –OH groups coordinating O_2. The cluster calculations predict that after the formation of the O_2 adduct, three bands can be formed in the hydroxo stretching region of the IR spectrum, shifted by –20, –40 and –110 cm^{-1} with respect to the corresponding peak position for the activated MOF (see Supplementary Table 47). A shift of ~ –20 cm^{-1} is computed both for Co(III)-OH species not interacting with O_2 (like in $1O_2$-1OH) and for (Co^{3+}-OH-Co^{2+})-OH species. The band at $\Delta\tilde{\nu}_{OH}$ ~ –110 cm^{-1} is assigned to Co(III)-OH in hydrogen bonding with the O_2 molecule and is obtained for both $1O_2$-1OH and $1O_2$-2OH. The $\Delta\tilde{\nu}_{OH}$ ~ –40 cm^{-1} band is predicted for the $1O_2$-0OH (corresponding to the Co(III)–OH closer to O_2) and the $1O_2$-2OH model (corresponding to the Co(III)–OH weakly interacting with O_2) while it is lacking in the $1O_2$-1OH one. This band is also predicted to be formed for (Co^{3+}-OH-Co^{2+})-OH with a low spin Co(II).

The experimental spectra for O_2 pressures lower than 5 mbar supports the formation of hydrogen bonds with adsorbed O_2. The signal that appears in the O–O stretching frequency region is redshifted with respect to the Raman gas phase value of an amount very close to that calculated for O_2 in hydrogen interaction with a –OH group. Moreover, the $\tilde{\nu}_{OH}$ region shows the appearance of three new signals that are computed for adsorption on Co-1OH and Co-2OH sites. At higher pressures, the $\tilde{\nu}_{O2}$ band formed at lower pressure grows slightly in intensity (associated with the adsorption on Co-1OH and Co-2OH sites) while a new signal appear as a shoulder at higher wavenumber that might be associated with the adsorption on Co-0OH sites. It should be noted that the physisorption of O_2 molecules on $O_2\cdots$Co complexes may also be affecting the IR spectra.

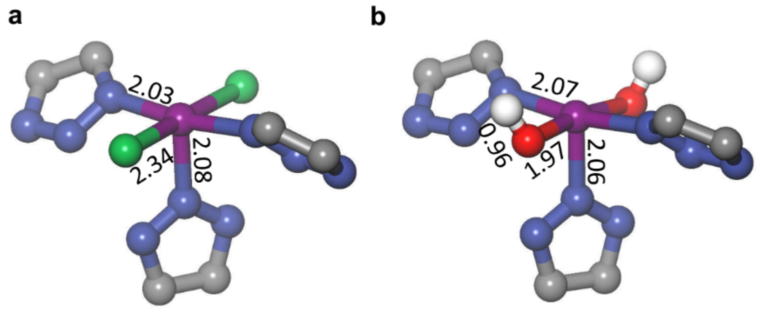

Supplementary Figure 85. Co geometry in the Co_4Cl and Co_4OH cluster models. First coordination sphere of Co in the **a**, Co_4Cl and **b**, Co_4OH, as obtained by optimization at the M06 level. The C atoms are reported in gray, H atoms in white, O in red, Cl in green, Co in purple and N in blue. All the bond distances are in Å.

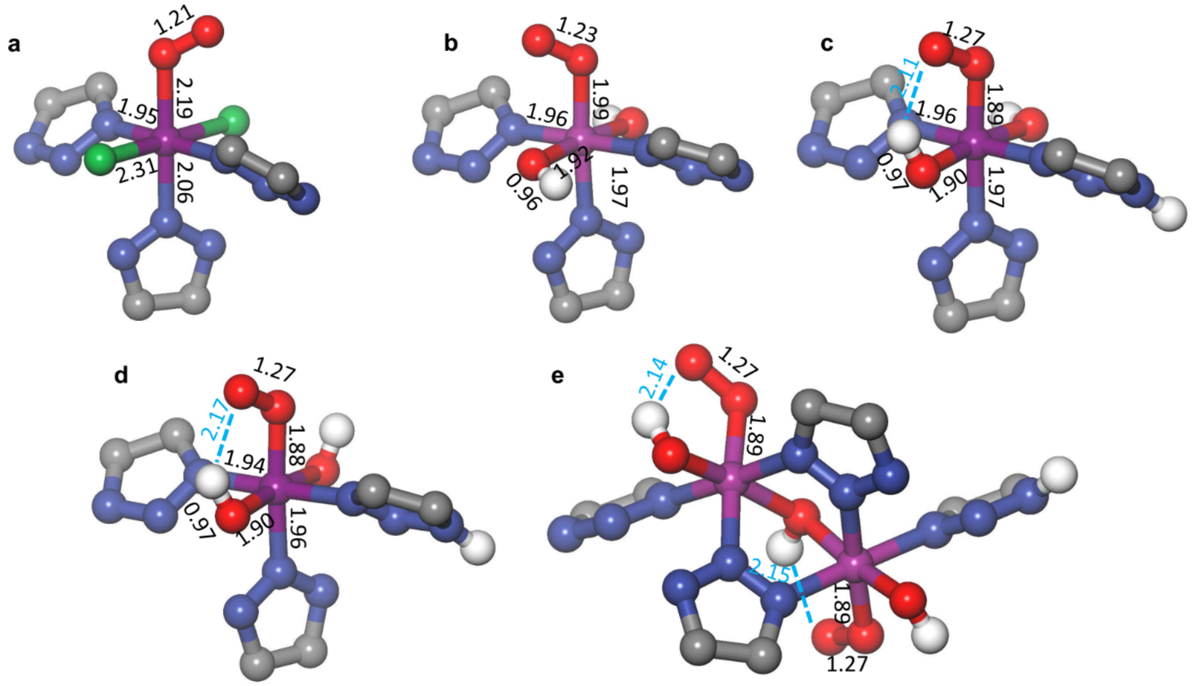

S

Supplementary Figure 86. Co geometry in the Co_4OH and Co_4Cl cluster models. First coordination sphere of Co in the **a**, Co_2Cl_2(bbta)-1O_2, **b**, $Co_2(OH)_2$(bbta)-1O_2-0OH, **c**, $Co_2(OH)_2$(bbta)-1O_2-1OH, **d**, $Co_2(OH)_2$(bbta)-1O_2-2OH, and **e**, $Co_2(OH)_2$(bbta)-2O_2-sfdp, as obtained by optimization at the M06 level. The C atoms are reported in gray, H atoms in white, O in red, Cl in green, Co in purple and N in blue. All the bond distances are in Å.

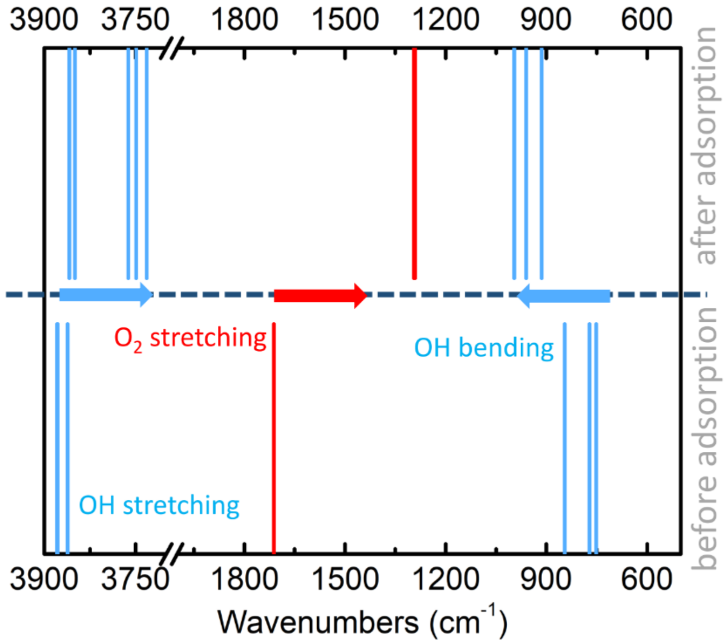

Supplementary Figure 87. FTIR spectra of $Co_2(OH)_2$(bbta). Computed IR bands for O_2 stretching (red lines) and for the OH stretching and bending $Co_2(OH)_2$(bbta) (light blue lines) before and after O_2 adsorption.

18. Supplementary Discussion: Summary of quantum chemical computations

Bicobalt and tetracobalt cluster models were adopted to study the electronic configuration of Co atoms in Co_2Cl_2(bbta) and $Co_2(OH)_2$(bbta). Multireference wave function based methods (CASSCF and CASPT2) and Kohn Sham DFT methods (M06, PBE0, and B3LYP) confirm that the cobalt atoms are in a high spin state configuration (see Supplementary Information Section 14). The antiferromagnetic coupling of the cobalt centers (S = 0) is computed to be the ground state for both systems, in agreement with the SQUID experiments. This configuration is more stable by only 4 kJ mol^{-1} than the corresponding ferromagnetic one (CASPT2 value, see Supplementary Table 30 and Supplementary Table 41). In the bicobalt clusters, the open-shell singlet wave function is a linear combination of about fifteen different electronic configurations having similar CI coefficients, indicating a large multiconfigurational character in these systems. The higher weight configurations consist on the six singly occupied orbitals (three α electrons and three β electrons distributed on the two cobalt atoms with equally weighted configurations, see Supplementary Figures 71 and 74.

Periodic and cluster models have been used to study the adsorption process of O_2 on Co_2Cl_2(bbta) and $Co_2(OH)_2$(bbta). All levels of calculations agree in indicating a higher affinity of O_2 for $Co_2(OH)_2$(bbta) than for Co_2Cl_2(bbta), as observed experimentally (see Supplementary Table 43 and Supplementary Table 47). Correspondingly, structural and electronic properties of the two O_2 complexes are significantly different. In $Co_2(OH)_2$(bbta), the adsorption process is a redox reaction where O_2 oxidizes Co^{2+} to Co^{3+} and is reduced to a superoxo species (O_2^-), according to all calculations. The spin density of Co and O_2 changes upon adsorption from 2.6 to 0.1 and from 1.8 to 1.1, respectively at the PBE0-D3 level (see Supplementary Table 43). The computed coordination distance d(Co–O_{O2}) is 1.87 Å and the d(O–O) distance is 1.31 Å, in agreement with the corresponding experimental values (see Supplementary Figure 81 and Supplementary Figure 87). The computed adsorption energy ΔE_{ads} for one O_2 molecule in the $Co_2(OH)_2$(bbta)-1O_2-2OH model amounts to −28 kJ mol^{-1} in the periodic calculations, underestimating the experimental value by about 20 kJ mol^{-1}.

For Co_2Cl_2(bbta), the PBE periodic structure agree with the M06 cluster models and indicate longer d(Co–O_{O2}) and a lower perturbation of the O_2 than in $Co_2(OH)_2$(bbta). According to the periodic calculations the two structures obtained by optimizing the complex considering the antiferromagnetic and the ferromagnetic coupling between the unpaired electrons of O_2 and of the cobalt(II) center are isoenergetic. The geometric parameters of these two structures are significantly different and they are reported in Supplementary Figure 80b and Supplementary Figure 81a. The cluster calculations support antiferromagnetic coupling in the ground state and predict a d(Co–O_{O2}) of 2.2 Å, 0.3 Å longer than that observed in the hydroxo framework, while d(O–O) is almost unchanged with respect to the O_2 gas phase value (see Supplementary Figure 86). $\Delta E_{\mathrm{ads}}^{\mathrm{c}}$ is computed to be repulsive for both the periodic and cluster models: O_2 will cause a high to low spin transition in the Co atoms, but it will not remain bound to it. This value is very close to the energetic cost computed for the high to low spin transition per Co in these MOFs (see Supplementary Information Section 16 and Supplementary Table 42).

The charge and the spin density calculated for the Co atoms in the two activated frameworks are equivalent (see Supplementary Tables 18, 19, and 43), and the different adsorption behavior of the two systems cannot be fully explained in terms of electrostatic basis. The change in coordination

environment does appear to contribute to the lowering of the binding energy of oxygen by 116.4 kJ mol^{-1} in PBE-D3 and 30.9 kJ mol^{-1} in PBE0-D3. However, the presence of hydroxo groups led us to investigate their effect on the adsorption properties in greater detail.

A closer inspection of the first coordination sphere around the Co center in the $Co_2(OH)_2$(bbta)-1O_2-2OH model (see Supplementary Figure 83) reveals that both hydroxo groups coordinated directly to Co are directly interacting with O_2. One of the hydroxo groups engages with O_2 in a very short hydrogen bond ($d(OH\cdots O_{O2})$ =1.95 Å) while the other has a weak interaction. Two additional models were also considered where O_2 is interacting with one (1O_2-1OH) or no hydroxo groups (1O_2-0OH). Correspondingly, three different Co sites can be identified, depending on the configuration of the –OH groups (and their availability to interact with adsorbate molecules): Co-2OH, Co-1OH, Co-0OH. These results are shown in Supplementary Figure 81f. Both PBE-D3 and PBE0-D3 indicate that the formation of the primary hydrogen bond stabilizes the complex by more than 20 kJ mol^{-1} (comparing the 1O_2-1OH model to the 1O2-0OH model). The coordination of the second hydroxo group (1O_2-2OH model) causes a further decrease of ΔE^{c}_{ads} by about 7 kJ mol^{-1}.

The comparison among the computed shifts in the hydroxo stretching frequency with those observed experimentally by FTIR spectroscopy at pressures lower than 5 mbar would support the coordination of at least one hydroxo group, and likely two, to the adsorbed O_2. The cluster calculations predict that after the formation of the O_2 adduct, three bands appear in the hydroxo stretching region of the IR spectrum, shifted by −20, −40 and −110 cm^{-1} with respect to the corresponding peak position for the activated framework (see Supplementary Table 47). A shift of ~ −20 cm^{-1} is computed both for Co^{3+}−OH species not interacting with O_2 (as in 1O_2-1OH) and for (Co^{3+}−OH−Co^{2+})−OH species. The band at $\Delta\tilde{\nu}_{OH}$ ~ −110 cm^{-1} is assigned to Co^{3+}−OH forming a hydrogen bond with the O_2 molecule and is present in both 1O_2-1OH and 1O_2-2OH. The $\Delta\tilde{\nu}_{OH}$ ~−40 cm^{-1} band is predicted for the 1O_2-0OH (corresponding to the Co^{3+}–OH closer to O_2) and the 1O_2-2OH models (corresponding to the Co^{3+}–OH weakly interacting with O_2) while it is lacking in the 1O_2-1OH model. This band occurs also in (Co^{3+}−OH−Co^{2+})−OH with a low spin Co(II). Experimentally, three signals are showed in the IR spectra at low loadings, though the shifts relative to the desolvated OH band do not directly correspond to the calculated shifts. In both the calculated and experimental spectra, the influence of O_2 is not just confined to the site of adsorption, but it also appears to change the vicinal Co sites, increasing the acidity of their –OH groups and shifting their –OH bands ((Co^{3+}−OH−Co^{2+})−OH). The adsorption of each O_2 molecule thus causes local differences in the surrounding environment. This phenomenon is well known in surface science as induced heterogeneity[30] and IR spectroscopy is a useful tool to follow it. Beside the –OH stretching mode, the –OH bending mode is computed to shift significantly upon O_2 adsorption ($\Delta\tilde{\delta}_{OH}$ ~+200 cm^{-1}), similar to what is observed experimentally, with a movement of the band from ~800 to 950 cm^{-1}. Another band observed at ~2000 cm^{-1}, which is absent in the computed spectra, is proposed to be a combination band of the perturbed $\tilde{\delta}_{OH}$ and $\tilde{\nu}_{O2}$.

These calculations indicate that the O_2 vibrational mode is significantly shifted upon the adsorption, as expected for a superoxo species ($\Delta\tilde{\nu}_{O2}$ ~ −410 cm^{-1}). The formation of the hydrogen bond would be responsible for about 17% of the shift, as can be appreciated comparing the values computed for 1O_2-0OH and 1O_2-1OH complexes in Supplementary Table 47, while it is essentially unchanged considering the interaction with the second –OH. For the 1O_2-1OH and 2OH models, two are the normal modes corresponding to the O–O stretching mode, equally combined with the

N–N stretching mode of the triazolate rings (see Supplementary Table 47). The intensity, position, and width of the O_2 band are the result of the perturbation of both the O–O stretching frequency and this ring mode. It should be noted that the appearance of a shoulder at higher wavenumber in the O–O stretching at higher pressures could be associated to the formation of complexes with Co-0OH sites at higher coverage.

For the $Co_2(OH)_2$(bbta), a model where all the Co atoms are coordinating a O_2 molecule was considered in order to simulate the high coverage situation ($Co_2(OH)_2$(bbta)-18O_2). This model has been used to verify if the calculations agree with the experiments indicating a decrease in the adsorption energy with the coverage. The $-\Delta E_{ads}$ is lower than that obtained for the $Co_2(OH)_2$(bbta)-1O_2-2OH model, though similar to that obtained for the $Co_2(OH)_2$(bbta)-1O_2-1OH model (see Supplementary Table 43). In the optimized $Co_2(OH)_2$(bbta)-18O_2 structure all adsorbed O_2 molecules are forming a 1O_2-1OH hydrogen-bonded complex. It should be noted that for Co:O_2 coverage larger than 0.5, hydroxo groups on cobalt atoms without adsorbed O_2 molecules may need to change their torsion angle (by 90°) in order to form a hydrogen bond to new adsorbed O_2 molecule. As a result, while the calculated structure of $Co_2(OH)_2$(bbta)-18O_2 corresponds to the global minimum of the system, experimentally the availability of the hydroxo species could be also influenced by their mobility. The difference in energy between a Co-2OH site and a Co-1OH site is of −0.7 and −8 kJ mol^{-1}, before and after O_2 coordination respectively. Considering a Boltzmann distribution at 195 K, less than 2% of the $Co_2(OH)_2$(bbta)-1O_2-2OH complex could convert to the Co-1OH one. O_2 adsorption on a Co-2OH site thus transforms the two vicinal Co sites in the less active Co-1OH or Co-0OH sites, creating heterogeneity in the material that was not present before. In the activated material, the population of the Co-2OH site can be roughly estimated as the 25% of the total on purely statistical basis. After the occupation of the Co-2OH site, the Co-1OH sites will be filled and then the Co-0OH ones. The difference in the isosteric heat of adsorption measured experimentally for Co:O_2 < 0.25 and Co:O_2 > 0.5 amounts to about 30 kJ mol^{-1}, which is close to the difference calculated between the 1O_2-2OH and the 1O_2-0OH models. The availability of hydroxo groups for hydrogen bonding could as a result help explain the evolution of the isosteric heat as a function of O_2 coverage. However, it could not be experimentally determined in this study to what degree this influences the O_2 adsorption properties, especially as the weakening in the ability of the cobalt centers to reduce O_2 upon partial oxidation is likely a factor, as well. Further studies will be necessary to help elucidate this problem.

19. Supplementary Note 1. Checkcif alerts and responses

For Co2Cl2bbta evacuated.cif:

_vrf_PLAT602_Co2Cl2bbta_evacuated
;
PROBLEM: VERY LARGE Solvent Accessible VOID(S) in Structure ! Info
RESPONSE: This amount of solvent accessible voids is common for desolvated and highly porous materials, of this framework is one.
;
_vrf_PLAT341_Co2Cl2bbta_evacuated
;

PROBLEM: Low Bond Precision on C-C Bonds .... 0.0125 Ang.
RESPONSE: This amount of precision is due to the nature of the measurement.
;

For Co2Cl2bbta O2.cif:

_vrf_PLAT430_Co2Cl2bbta_O2
;
PROBLEM: Short Inter D...A Contact O1 ..O2 . 1.12 Ang.
RESPONSE: These two atoms are of a physisorbed O2 adduct, which would have an unperturbed O-O bond length relative to free O2. There is some disorder, hence the bond length is slightly shorter then expected.
;
_vrf_PLAT601_Co2Cl2bbta_O2
;
PROBLEM: Structure Contains Solvent Accessible VOIDS of . 250 Ang**3
RESPONSE: This amount of solvent accessible voids is common for desolvated and highly porous materials, of this framework is one.
;
_vrf_PLAT241_Co2Cl2bbta_O2
;
PROBLEM: High 'MainMol' Ueq as Compared to Neighbors of Co1 Check
RESPONSE: This is due to the nature of the measurement.
;
_vrf_PLAT242_Co2Cl2bbta_O2
;
PROBLEM: Low 'MainMol' Ueq as Compared to Neighbors of N2 Check
RESPONSE: This is due to the nature of the measurement.
;
_vrf_PLAT341_Co2Cl2bbta_O2
;
PROBLEM: Low Bond Precision on C-C Bonds .... 0.0115 Ang.
RESPONSE: This amount of precision is due to the nature of the measurement.
;

For Co2OH2bbta desolvated.cif:

_vrf_PLAT602_Co2OH2bbta_desolvated
;
PROBLEM: VERY LARGE Solvent Accessible VOID(S) in Structure ! Info
RESPONSE: This amount of solvent accessible voids is common for desolvated and highly porous materials, of this framework is one.
;
_vrf_PLAT341_Co2OH2bbta_desolvated

;
PROBLEM: Low Bond Precision on C-C Bonds .... 0.0155 Ang.
RESPONSE: This amount of precision is due to the nature of the measurement.
;

For COBBTA-BARE-6K-final.cif:
_vrf_REFI015_COBBTA-BARE-WORKING2_publ
;
PROBLEM: _refine_ls_shift/su_max is missing
RESPONSE: This is not single crystal data.
;
_vrf_CELLK01_COBBTA-BARE-WORKING2_publ
;
PROBLEM: Check that the cell measurement temperature is in Kelvin.
RESPONSE: Temperature is indeed in Kelvin.
;
_vrf_CRYSC01_COBBTA-BARE-WORKING2_publ
;
PROBLEM: No recognised colour has been given for crystal colour.
RESPONSE: Material is light brown, but is powder not single crystal.
;
_vrf_PLAT602_COBBTA-BARE-WORKING2_publ
;
PROBLEM: VERY LARGE Solvent Accessible VOID(S) in Structure ! Info
RESPONSE: This is well known and expected for this structure.
;
_vrf_PLAT701_COBBTA-BARE-WORKING2_publ
;
PROBLEM: Bond Calc 30.733(6), Rep 1.327(4), Dev.. 4901.00 Sigma
RESPONSE: Program which calculated these messed up some.
;
_vrf_PLAT702_COBBTA-BARE-WORKING2_publ
;
PROBLEM: Angle Calc 124.1(2), Rep 114.1(4), Dev.. 50.00 Sigma
RESPONSE: Program which calculated these messed up some.
;
_vrf_PLAT041_COBBTA-BARE-WORKING2_publ
;
PROBLEM: Calc. and Reported SumFormula Strings Differ Please Check
RESPONSE: Difference appears to be 0.01 on molecular weight...
;

For CO2OH2bbta 1bar O2.cif:

_vrf_PLAT430_Co2OH2bbta_1barO2

;
PROBLEM: Short Inter D...A Contact O3   ..O3   .   2.27 Ang.
RESPONSE: The O3 atom is indeed hydrogen bonding with O1 (and is partially occupied and disordered.)
;
_vrf_PLAT601_Co2OH2bbta_1barO2
;
PROBLEM: Structure Contains Solvent Accessible VOIDS of .   315 Ang**3
RESPONSE: This amount of solvent accessible voids is common for desolvated and highly porous materials, of this framework is one.
;
_vrf_PLAT341_Co2OH2bbta_1barO2
;
PROBLEM: Low Bond Precision on C-C Bonds ....   0.0125 Ang.
RESPONSE: This amount of precision is due to the nature of the measurement.
;

For COBBTA-O2-8K-final.cif:
_vrf_REFI015_COBBTA-O2-DOSED_publ
;
PROBLEM: _refine_ls_shift/su_max is missing
RESPONSE: This is not single crystal data.
;
_vrf_CELLK01_COBBTA-O2-DOSED_publ
;
PROBLEM: Check that the cell measurement temperature is in Kelvin.
RESPONSE: Temperature is indeed in Kelvin.
;
_vrf_CRYSC01_COBBTA-O2-DOSED_publ
;
PROBLEM: No recognised colour has been given for crystal colour.
RESPONSE: Material is light brown, but is powder not single crystal.
;
_vrf_PLAT601_COBBTA-O2-DOSED_publ
;
PROBLEM: Structure Contains Solvent Accessible VOIDS of .   349 Ang**3
RESPONSE: This is well known and expected for this structures.
;
_vrf_PLAT701_COBBTA-O2-DOSED_publ
;
PROBLEM: Bond   Calc 27.674(13), Rep   2.380(11), Dev..   1945.69 Sigma
RESPONSE: Program which calculated these messed up some.
;
_vrf_PLAT702_COBBTA-O2-DOSED_publ
;

PROBLEM: Angle Calc 93.7(3), Rep 102.8(7), Dev.. 30.33 Sigma
RESPONSE: Program which calculated these messed up some.
;
_vrf_PLAT041_COBBTA-O2-DOSED_publ
;
PROBLEM: Calc. and Reported SumFormula Strings Differ Please Check
RESPONSE: Difference appears to be 0.02 on molecular weight...
;

For COBBTA-O2-150K-final.cif:
_vrf_REFI015_COBBTA-O2-DOSED-150K_publ
;
PROBLEM: _refine_ls_shift/su_max is missing
RESPONSE: This is not single crystal data.
;
_vrf_CRYSC01_COBBTA-O2-DOSED-150K_publ
;
PROBLEM: No recognised colour has been given for crystal colour.
RESPONSE: Material is light brown, but is powder not single crystal.
;
_vrf_PLAT430_COBBTA-O2-DOSED-150K_publ
;
PROBLEM: Short Inter D...A Contact O3 ..O3 . 1.86 Ang.
RESPONSE: These positions have 0.5 occupancy and are not both occupied at the same time.
;
_vrf_PLAT601_COBBTA-O2-DOSED-150K_publ
;
PROBLEM: Structure Contains Solvent Accessible VOIDS of . 324 Ang**3
RESPONSE: This is well known and expected for this structure.
;
_vrf_PLAT701_COBBTA-O2-DOSED-150K_publ
;
PROBLEM: Bond Calc 27.757(13), Rep 2.335(12), Dev.. 1955.54 Sigma
RESPONSE: Program which calculated these messed up some.
;
_vrf_PLAT702_COBBTA-O2-DOSED-150K_publ
;
PROBLEM: Angle Calc 93.5(4), Rep 98.8(7), Dev.. 13.25 Sigma
RESPONSE: Program which calculated these messed up some.
;
_vrf_PLAT041_COBBTA-O2-DOSED-150K_publ
;
PROBLEM: Calc. and Reported SumFormula Strings Differ Please Check
RESPONSE: Difference appears to be 0.02 on molecular weight...
;

20. Supplementary References

1. Walton, K. S. & Snurr, R. Q. Applicability of the BET Method for Determining Surface Areas of Microporous Metal−Organic Frameworks. *J. Am. Chem. Soc.* **129**, 8552–8556 (2007).
2. Lu, X. F. *et al.* An alkaline-stable, metal hydroxide mimicking metal–organic framework for efficient electrocatalytic oxygen evolution. *J. Am. Chem. Soc.* **138**, 8336–8339 (2016).
3. Lotmar, W. & Feitknecht, W. Über Änderungen der Ionenabstände in Hydroxyd - Schichtengittern. *Zeitschrift für Krist. - Cryst. Mater.* **93**, 368–378 (1936).
4. Momma, K. & Izumi, F. VESTA 3 for three-dimensional visualization of crystal, volumetric and morphology data. *J. Appl. Crystallogr.* **44**, 1272–1276 (2011).
5. Lacy, D. C., Park, Y. J., Ziller, J. W., Yano, J. & Borovik, A. S. Assembly and properties of heterobimetallic $Co^{II/III}/Ca^{II}$ complexes with aquo and hydroxo ligands. *J. Am. Chem. Soc.* **134**, 17526–17535 (2012).
6. Park, Y. J. *et al.* Heterobimetallic complexes with M^{III}-(μ-OH)-M^{II} cores (M^{III} = Fe, Mn, Ga; M^{II} = Ca, Sr, and Ba): structural, kinetic, and redox properties. *Chem. Sci.* **4**, 717–726 (2013).
7. MacBeth, C. E., Hammes, B. S., Young, V. G. & Borovik, A. S. Hydrogen-bonding cavities about metal ions: Synthesis, structure, and physical properties for a series of monomeric M-OH complexes derived from water. *Inorg. Chem.* **40**, 4733–4741 (2001).
8. Jones, J. R., Ziller, J. W. & Borovik, A. S. Modulating the primary and secondary coordination spheres within a series of Co^{II}-OH complexes. *Inorg. Chem.* **56**, 1112–1120 (2017).
9. Perdew, J. P., Burke, K. & Ernzerhof, M. Generalized gradient approximation made simple. *Phys. Rev. Lett.* **77**, 3865–3868 (1996).
10. Kresse, G. & Furthmüller, J. Efficiency of ab-initio total energy calculations for metals and semiconductors using a plane-wave basis set. *Comput. Mater. Sci.* **6**, 15–50 (1996).
11. Grimme, S., Antony, J., Ehrlich, S. & Krieg, H. A consistent and accurate ab initio parametrization of density functional dispersion correction (DFT-D) for the 94 elements H-Pu. *J. Chem. Phys.* **132**, 154104 (2010).
12. Ioannidis, E. I. & Kulik, H. J. Ligand-field-dependent behavior of meta-GGA exchange in transition-metal complex spin-state ordering. *J. Phys. Chem. A* **121**, 874–884 (2017).
13. Bowman, D. N. & Jakubikova, E. Low-spin versus high-spin ground state in pseudo-octahedral iron complexes. *Inorg. Chem.* **51**, 6011–6019 (2012).
14. Reiher, M. Theoretical study of the $Fe(phen)_2(NCS)_2$ spin-crossover complex with reparametrized density functionals. *Inorg. Chem.* **41**, 6928–6935 (2002).
15. Perdew, J. P., Ernzerhof, M. & Burke, K. Rationale for mixing exact exchange with density functional approximations. *J. Chem. Phys.* **105**, 9982–9985 (1996).
16. Xiao, D. J. *et al.* Selective, tunable O_2 binding in cobalt(II)–triazolate/pyrazolate metal–organic frameworks. *J. Am. Chem. Soc.* **138**, 7161–7170 (2016).
17. Zhao, Y. & Truhlar, D. G. The M06 suite of density functionals for main group thermochemistry, thermochemical kinetics, noncovalent interactions, excited states, and transition elements: Two new functionals and systematic testing of four M06-class functionals and 12 other function. *Theor. Chem. Acc.* **120**, 215–241 (2008).
18. Frisch, M. J. et al. Gaussian 09. (2009).
19. Verma, P., Maurice, R. & Truhlar, D. G. Adsorbate-induced changes in magnetic interactions in $Fe_2(dobdc)$ with adsorbed hydrocarbon molecules. *J. Phys. Chem. C* **120**,

9933–9948 (2016).
20. Valero, R., Costa, R., de P. R. Moreira, I., Truhlar, D. G. & Illas, F. Performance of the M06 family of exchange-correlation functionals for predicting magnetic coupling in organic and inorganic molecules. *J. Chem. Phys.* **128**, 114103 (2008).
21. Stauber, J. M. *et al.* Pushing single-oxygen-atom-bridged bimetallic systems to the right: A cryptand-encapsulated Co-O-Co unit. *J. Am. Chem. Soc.* **137**, 15354–15357 (2015).
22. Lee, C., Yang, W. & Parr, R. G. Development of the Colle-Salvetti correlation-energy formula into a functional of the electron density. *Phys. Rev. B* **37**, 785–789 (1988).
23. Becke, A. D. Density-functional exchange-energy approximation with correct asymptotic behavior. *Phys. Rev. A* **38**, 3098–3100 (1988).
24. Weigend, F. Accurate Coulomb-fitting basis sets for H to Rn. *Phys. Chem. Chem. Phys.* **8**, 1057–1065 (2006).
25. Weigend, F. & Ahlrichs, R. Balanced basis sets of split valence, triple zeta valence and quadruple zeta valence quality for H to Rn: Design and assessment of accuracy. *Phys. Chem. Chem. Phys.* **7**, 3297–3305 (2005).
26. Aquilante, F. *et al.* Molcas 8: New capabilities for multiconfigurational quantum chemical calculations across the periodic table. *J. Comput. Chem.* **37**, 506–541 (2016).
27. Marenich, A. V., Cramer, C. J. & Truhlar, D. G. Cm5pac. (2011).
28. Marenich, A. V., Jerome, S. V., Cramer, C. J. & Truhlar, D. G. Charge model 5: An extension of hirshfeld population analysis for the accurate description of molecular interactions in gaseous and condensed phases. *J. Chem. Theory Comput.* **8**, 527–541 (2012).
29. Vitillo, J. G. & Ricchiardi, G. Effect of pore size, solvation, and defectivity on the perturbation of adsorbates in MOFs: The paradigmatic Mg_2(dobpdc) case study. *J. Phys. Chem. C* **121**, 22762–22772 (2017).
30. Fubini, B., Bolis, V., Cavenago, A., Garrone, E. & Ugliengo, P. Structural and induced heterogeneity at the surface of some SiO_2 polymorphs from the enthalpy of adsorption of various molecules. *Langmuir* **9**, 2712–2720 (1993).
